# Bioentity2vec: Attribute- and behavior-driven representation for predicting multi-type relationships between bioentities

**DOI:** 10.1093/gigascience/giaa032

**Published:** 2020-06-13

**Authors:** Zhen-Hao Guo, Zhu-Hong You, Yan-Bin Wang, De-Shuang Huang, Hai-Cheng Yi, Zhan-Heng Chen

**Affiliations:** 1 XinJiang Laboratory of Minority Speech and Language Information Processing, Xinjiang Technical Institute of Physics and Chemistry, Chinese Academy of Sciences, Urumqi 830011, No. 40-1, Beijing South Road, Urumqi, Xinjiang, China; 2 University of Chinese Academy of Sciences, Beijing 100049, China; 3 School of Cyber Science and Technology, Zhejiang University, Hangzhou 310000, Zhejiang, China; 4 Computer Science Department, Tongji University, Shanghai 200000, China

**Keywords:** network biology, system biology, Bioentity2vec, multi-type relationship prediction

## Abstract

**Background:**

The explosive growth of genomic, chemical, and pathological data provides new opportunities and challenges for humans to thoroughly understand life activities in cells. However, there exist few computational models that aggregate various bioentities to comprehensively reveal the physical and functional landscape of biological systems.

**Results:**

We constructed a molecular association network, which contains 18 edges (relationships) between 8 nodes (bioentities). Based on this, we propose Bioentity2vec, a new method for representing bioentities, which integrates information about the attributes and behaviors of a bioentity. Applying the random forest classifier, we achieved promising performance on 18 relationships, with an area under the curve of 0.9608 and an area under the precision-recall curve of 0.9572.

**Conclusions:**

Our study shows that constructing a network with rich topological and biological information is important for systematic understanding of the biological landscape at the molecular level. Our results show that Bioentity2vec can effectively represent biological entities and provides easily distinguishable information about classification tasks. Our method is also able to simultaneously predict relationships between single types and multiple types, which will accelerate progress in biological experimental research and industrial product development.

## Introduction

In the post-genomic era, a key task is to systematically and comprehensively understand the relationships between bioentities in living cells [1]. The foundation for this mission is the rapid development of high-throughput technologies and the discovery of new transcripts or translations [2]. For example, the increasing evidence prove that the biomolecule networks such as protein-protein interaction network, ncRNA-disease association network, drug-target interaction network play important roles in protein synthesis [3], gene expression [4], RNA processing [5], and developmental regulation [6]. Consequently, research into the relationships between bioentities will not only provide novel insights into life processes but also facilitate disease prevention, diagnosis, treatment, and drug development.

Wet lab experiments to identify relationships between bioentities in large-scale datasets are labor-intensive and time-consuming and have limited real-world utility. Meanwhile, the extensive amount of accumulated experimental data causes information overload, which makes it prohibitively costly to acquire valuable knowledge. Hence, biological experiments can be effectively guided by data-based computer modeling methods to accelerate genomics and proteomics research progress [7].

The computational biology community has developed many computational methods, such as matrix factorization [8], machine learning [9], and network analysis [10] to detect previously unknown relationships between entities. Guo et al. proposed a computational model to predict potential associations between diseases and long noncoding RNA (lncRNA) by integrating evidence of known associations with disease semantic similarity [11]. Wang et al. adopted the logistic model tree methodology to integrate information from multiple sources to discover unknown associations between diseases and microRNA (miRNA) [12]. Li et al. used the position-specific scoring matrix to represent proteins, and then put these into an ensemble classifier to predict self-interacting and non–self-interacting proteins [13]. Wang et al. used rotation forest to discover unknown drug–target interactions by drug structure and protein sequence [14].

However, the incompleteness of the data constrains the credibility of predictions made by these methods, resulting in high false-positive and false-negative rates [15]. In recent years, the discovery of new types of bioentities and their relationships has provided novel insights to improve this situation to some extent. Additional bioentities may be considered as bridges to synergistically facilitate our knowledge of underlying biological principles and improve prediction. For example, Chen et al. were able to effectively improve the prediction of miRNA–disease associations by taking environmental factors into account [16]. Similarly, Cui et al. drew from gene expression data to make preliminary explorations into predicting drug–disease associations [17].

In the past few years, much molecular data have accumulated, but computational methods have failed to make significant breakthroughs because few people regard cells as being complete units. In fact, cells comprise nodes (bioentities) and edges (relationships), much like a network (graph), to maintain normal life activities and physiological functions. The ability to establish connections between internal or external factors and gene expression would be helpful for understanding biological systems. Here, we constructed a molecular association network (MAN), based on various online databases, such as NONCODE [18] and miRbase [19], to help systematically analyze the relationships between bioentities within human cells.

Faced with such a large-scale network, the most critical challenge is how to quickly and effectively describe the nodes. In general, each bioentity can be defined by its own attributes and behaviors [20, 21]. Attribute features can be represented by RNA sequences, drug chemical structures, etc. [22–24]. The semantic description of drug or disease can also be considered as a kind of representation, which is widely used in relationship prediction tasks, such as drug reposition [24]. On the other hand, network-based methods, especially the rapid development of graph-embedding (network representation) algorithms, has provided great hope for being able to clearly describe relationships between nodes [25–32].

Graph embedding, in which nodes are represented in a network as dense vector forms, is chosen to respond to this situation [33]. Although some existing bioinformatics models contain the idea of graph embedding, many still focus on traditional techniques, including principal component analysis [34], multidimensional scaling [35], Isomap [36], and local linear embeddings [37]. In general, these methods offer satisfactory performance for small networks. However, at least quadratic time complexity restricts the application of these methods to large-scale data. Recently, deep learning has attracted research attention. Here, the representation method DeepWalk is applied.

**Figure 1:**
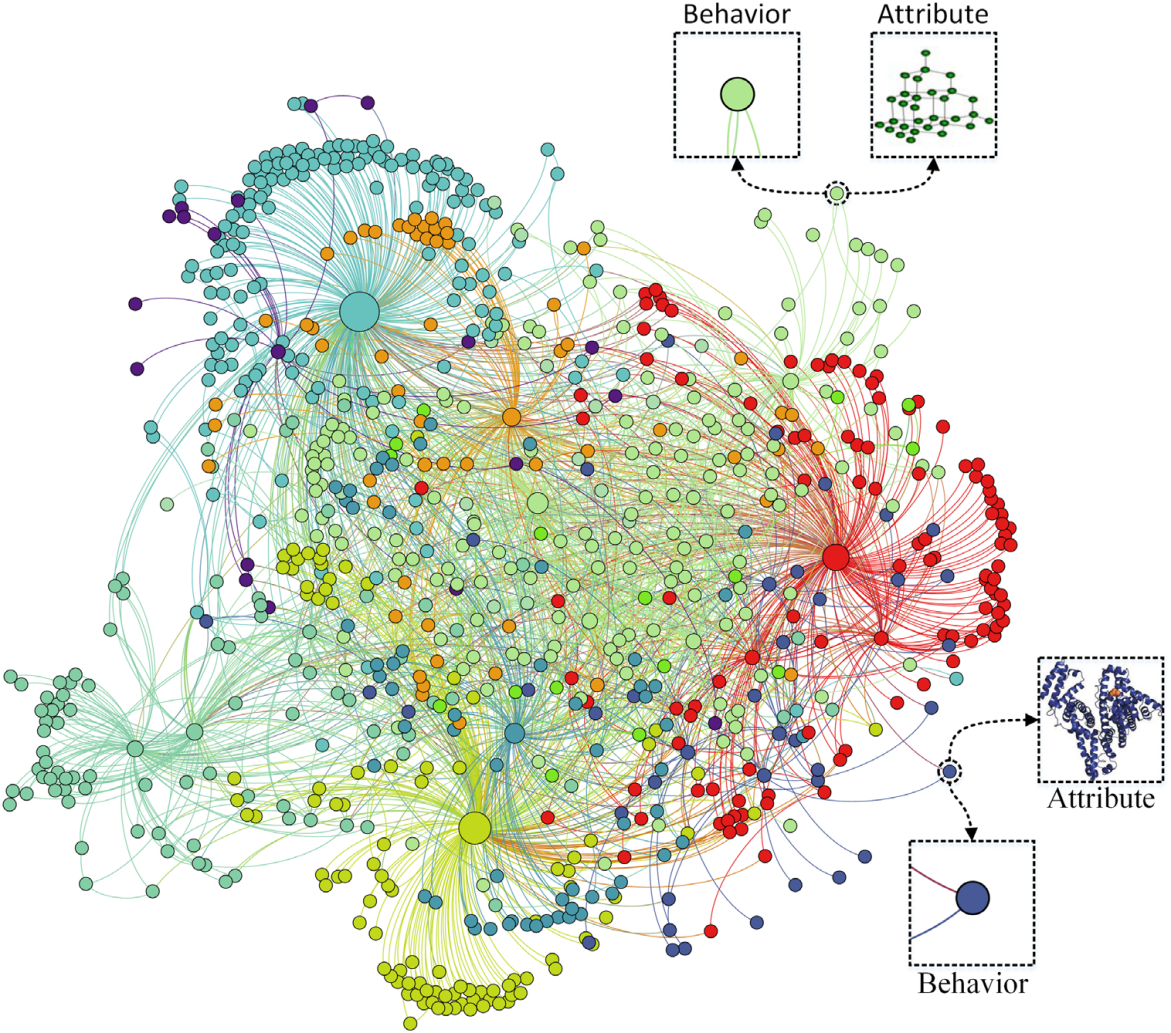
An example of visualization based on molecular association networks (MAN), in which different colors represent different types of bioentities. Each bioentity contains 2 kinds of information: node behavior (relationships with other nodes) and node attribute (sequences of protein or RNA, chemical structure of drug, and semantics of disease and microbe).

**Figure 2:**
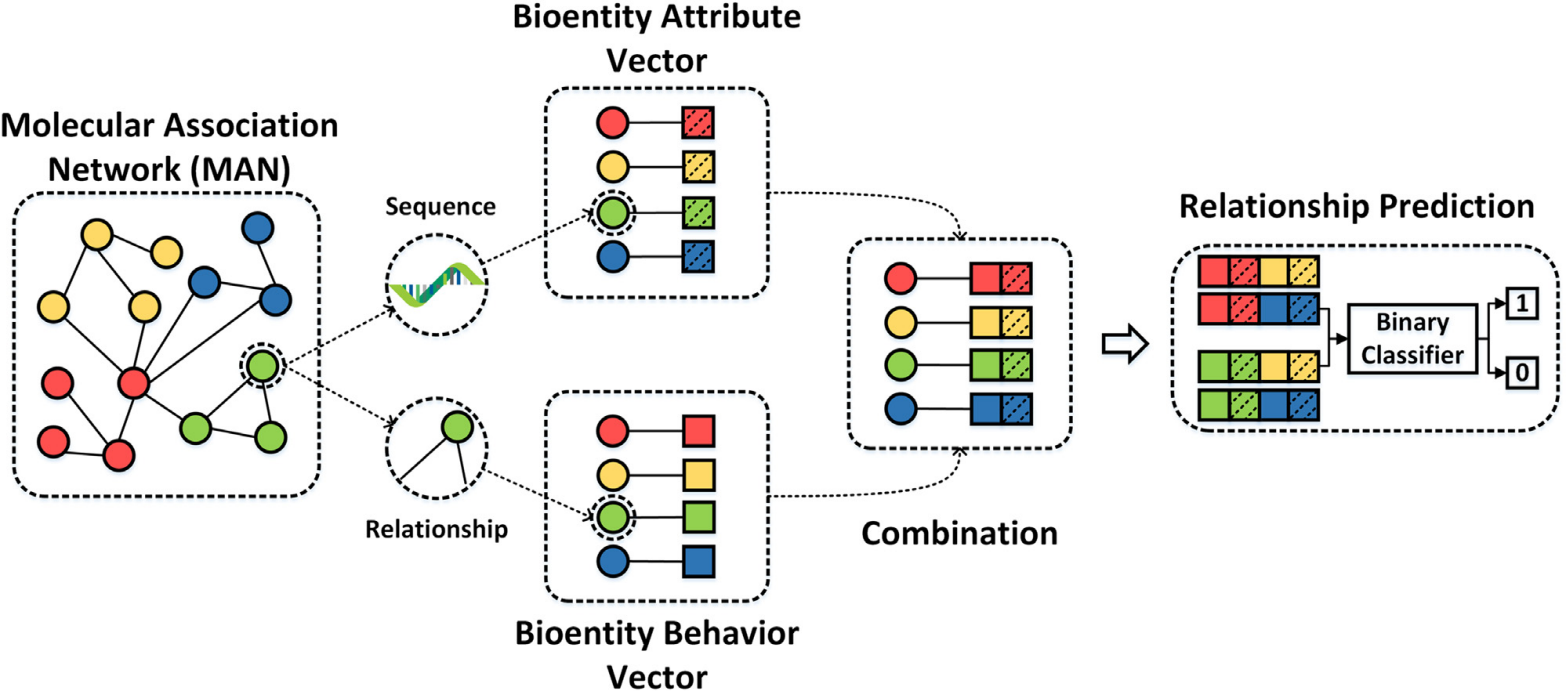
Flow chart of the proposed method. Each node in the network can be described in 2 ways: (i) by attribute feature, such as sequence and chemical structure, which can be learned as a 64-dimension vector by *k*-mer, etc., and (ii) by behavior feature, which can be represented as a 64-dimension vector through DeepWalk. Attribute and behavior feature are distinguished by dashed and unprocessed squares. After combining attribute and behavior information, each node can be represented as a 128-dimension vector. Positive samples are experimentally verified relationships, while negative samples are the same number of unlabeled relationships that are randomly selected in matrix *A*. Taking the low-dimensional dense vectors as input, random forest is used for prediction.

We constructed a MAN and propose a graph-embedding algorithm to represent each node as a vector (Fig. 1). Specifically, 18 kinds of associations or interactions between 8 kinds of biomolecules were collected from various databases to construct the network. The lower triangular part of the adjacency matrix, *A*, simplifies calculation and storage. Each bioentity can be represented as a vector by combining attribute and behavior features (see flow chart in Fig. 2). We used random forest to predict multi-type relationships across an entire network, obtaining an area under the receiver operating characteristic curve (AUC) of 0.9608, and an area under the precision-recall curve (AUPR) of 0.9572, using 5-fold cross-validation. Furthermore, we implemented 3 experiments to compare feature importance, embedding strategy, and proportions of training sets. Our results suggest the potential utility of MAN for revealing previously uncovered relationships. We hope that this work can provide assistance and guidance for wet experiments and be useful for researchers seeking to understand gene regulation and disease mechanisms and to discover new drugs at the molecular level.

## Materials and Methods

### Construction of the molecular association network

**Figure 3:**
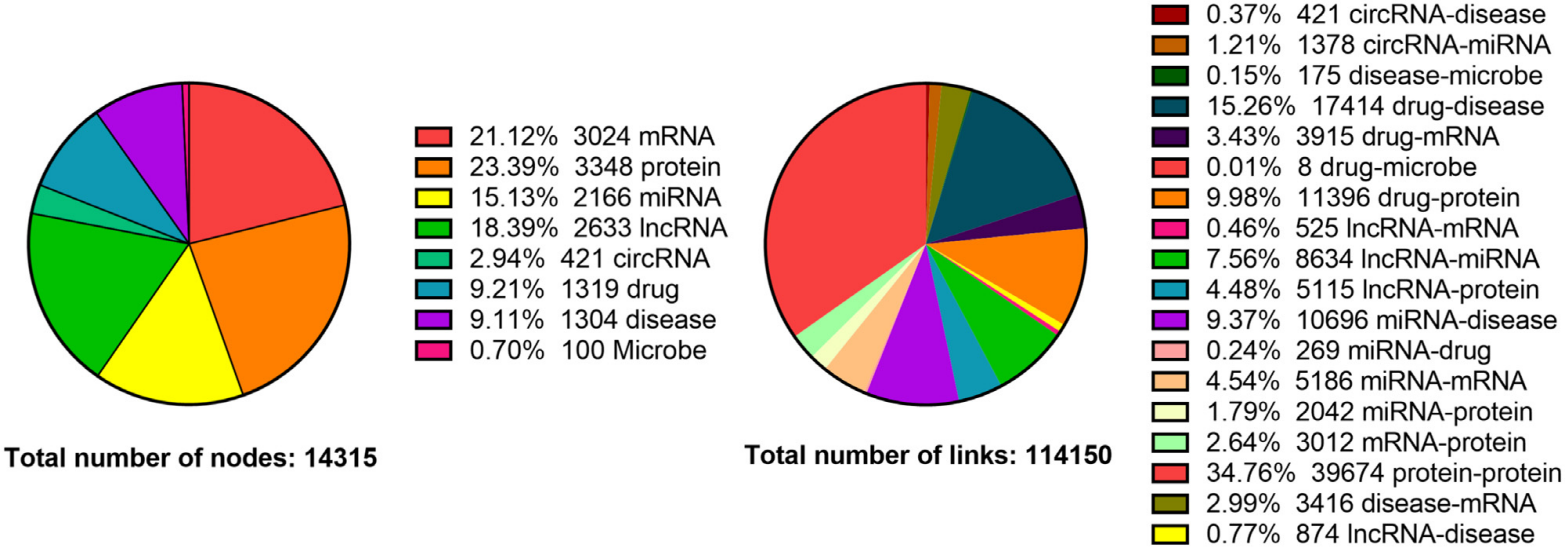
Details about the quantity and distribution of 8 kinds of biomolecules and 18 kinds of relationships.

To construct the MAN, 18 different experimentally verified associations or interactions were collected from various databases [38–57]. After unifying identifiers, we obtained 8 types of bioentity. All relationships and bioentities were then aggregated to form the MAN. The quantity and proportion of each type of bioentity or relationships is shown in Fig. 3.

### Node attribute representation: *k*-mer, semantics, and fingerprint

Protein and RNA sequences, disease and microbe semantics, and drug chemical structure are all essential features. We represented these as vectors using the following methods.

For protein, mRNA, miRNA, lncRNA, and circular RNA (circRNA), sequences were collected from STRING (STRING, RRID:SCR_005223) [56], NCBI (NCBI, RRID:SCR_006472) [58], miRBase (miRbase, RRID:SCR_003152) [19], NONCODE (NONCODE, RRID:SCR_007822) [18], and circBase [59], respectively. Proteins are composed of 20 different amino acids; using the method described by Shen et al. [60], we first classified these into 4 categories based on amino acid side chain polarity: (i) alanine, valine, leucine, isoleucine, methionine, phenylalanine, tryptophan, and proline; (ii) glycine, serine, threonine, cysteine, asparagine, glycine, and tyrosine; (iii) arginine, lysine, histidine; and (iv) aspartate and glutamic acid. RNA, including mRNA, miRNA, lncRNA, and circRNA, is composed of 4 nucleotides: adenine (A), guanine (G), cytosine (C), and uracil (U), with the same sequence composition, so we directly encode their original sequences without pre-treatment. Each RNA molecule or protein can be represented as a vector by *k*-mer, in which all dimensions represent the full permutation of *k* nucleotide (or amino acid) combinations, and the value of each dimension is the normalized frequency of the corresponding *k*-mer appearing in the sequence. In this article, *k* = 3, and each protein or RNA can be represented as a 64-dimension (4^3^ = 4 × 4 × 4) vector.

**Figure 4:**
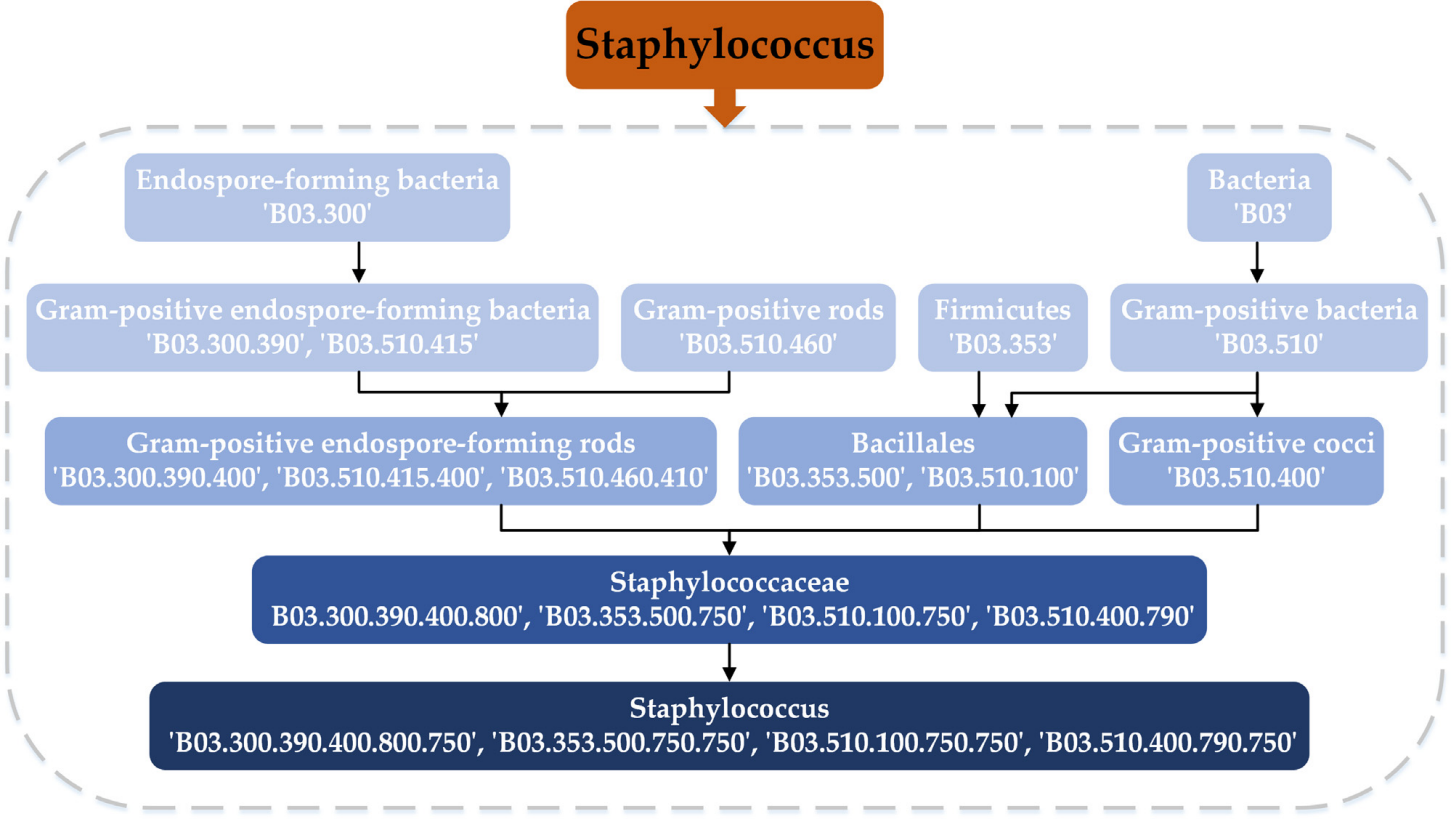
Construction of the directed acyclic graph (DAG) of *Staphylococcus*. The father node of the current microbe can be obtained by deleting the last 3 digits of the descriptor. For example, for *Bacillales* (B03.353.500, B03.510.100), we can remove the last 3 digits to get *Firmicutes* (B03.353) and gram-positive bacteria (B03.510).

Diseases and microbes were characterized using Medical Subject Headings (MeSH) descriptors. Top-level categories in the MeSH tree structure are anatomy [A], organisms [B], diseases [C], and so on. The categories corresponding to microbes and diseases are B and C, respectively. As done by Wang et al. [23], we construct a directed acyclic graph (DAG) of diseases and microbes (see Fig. 4) to represent them through their semantics. For example, a microbe *M* can be represented as a graph DAG*(M)*= (*M, N*(*M*)*, E*(*M*)), where *N*(*M*) is the set of all nodes in *M*’s DAG and *E*(*M*) is the set of all edges in *M*’s DAG. The semantic contribution of microbe *m*, which is in the node set *N*(*M*) to *M*, can be defined as:

(1) $$\begin{eqnarray*}
\left\{ {\begin{array}{@{}*{1}{c}@{}} {{V_M}\ \left( m \right) = \ 1\ \mathrm{if}\ m\ = \ M}\\ {{V_M}\ \left( m \right) = \max \left\{ {\Delta * V\left( {m^{\prime}} \right)|m^{\prime} \in \mathrm{children}\ \mathrm{of}\ m} \right\}\ \mathrm{if}\ m \ne M} \end{array}} \right.
\end{eqnarray*}$$

where Δ denotes an attenuation factor and is defined as 0.5, according to previous literature [23]. In the DAG generated by microbe *M, M*’s contribution to itself can be regarded as the maximum and is equal to 1; the remaining diseases will contribute less and less to *M* as the distance increases. Therefore, the sum of the contributions of microbes, which are in the set *N*(*M*) to *M*, can be calculated as follows:

(2) $$\begin{eqnarray*}
\mathrm{SV}\ \left( M \right) = {\Sigma _{m \in N\left( M \right)}}\ {V_M}\left( m \right)
\end{eqnarray*}$$

The similarity between microbes *i* and *j* can then be calculated as follows:

(3) $$\begin{eqnarray*}
\mathrm{Similarity}\ \left( {i,j} \right) = \frac{{\mathop \sum \nolimits_{m \in N\left( i \right)\mathop \cap \nolimits^ N\left( j \right)} \left[ {{V_i}\left( m \right) + {V_j}\left( m \right)} \right]}}{{\mathrm{SV}\left( i \right) + \mathrm{SV}\left( j \right)}}\
\end{eqnarray*}$$

The node attribute of microbe or disease can be represented by semantics similarity, which is converted into a 64-dimensional vector after feature extraction and transformation using the stack autoencoder. A DAG example of the microbe *Staphylococcus* is as follows: for drugs, we download their Simplified Molecular Input Line Entry Specification (SMILES) [61] from DrugBank (DrugBank, RRID:SCR_002700) [47]. Then, SMILES is transformed into corresponding Morgan molecular fingerprints [62] using the Python package RDKit (RDKit, RRID:SCR_014274) [63]. To unify dimensions and improve feature quality, stack autoencoder is used to convert each original molecular fingerprint into a 64-dimensional vector.

### Node behavior representation: DeepWalk

Using “guilt-by-association” assumptions, we use a more general behavioral feature in complex networks. Generally speaking, this involves embedding representations of known edges between nodes in a network. Despite this, a row or column of the adjacency matrix can directly be used as a representation vector for node behavior in a one-hot encoding method. However, there is no concept of similarity between each dimension of such high-dimensional, sparse vectors because it is represented as indices in a relationship. Meanwhile, the one-hot encoding method takes up a lot of storage space and is not conducive to the input of downstream tasks. Hence, how to extract information about behavior from nodes in complex networks such as a MAN is challenging.

Here, we use a network embedding method called DeepWalk [64]. The main idea is to obtain a certain length of the walk sequence through random walk, an ideal mathematical state of Brownian motion that can repeatedly access the visited nodes. After obtaining enough sequences, the vectors of the nodes can be learned by the SkipGram model. The direct analog is to estimate the likelihood of observing vertex ${v_i}$, given all the previous vertices visited so far in the random walk, i.e.,

(4) $$\begin{eqnarray*}
{P_r}\left( {\left. {{v_i}} \right|\left( {{v_1},{v_2}, \ldots ,{v_{i - 1}}} \right)} \right).
\end{eqnarray*}$$

The goal is to learn a latent representation, and the mapping function is:

(5) $$\begin{eqnarray*}
\Phi :v \in V \mapsto {R^{\left| V \right| \times d}}.
\end{eqnarray*}$$

The problem, then, is to estimate the likelihood:

(6) $$\begin{eqnarray*}
{P_r}\left( {\left. {{v_i}} \right|\left( {\Phi ({v_1}),\Phi \left( {{v_2}} \right), \ldots ,\Phi \left( {{v_{i - 1}}} \right)} \right)} \right).
\end{eqnarray*}$$

The recent relaxation in language modeling turns the prediction problem, and this yields the optimization problem:

(7) $$\begin{eqnarray*}
\mathop {\mathrm{minimize}}\limits_\Phi = \ - \mathrm{log}{P_r}(\left\{ {{v_{i - w}}, \ldots ,{v_{i + w}}} \right\}\backslash {v_i}|\Phi \left( {{v_i}} \right)).
\end{eqnarray*}$$

The main steps of the algorithm are as follows:

**Table:** 

Algorithm 1: DeepWalk (${\boldsymbol{G}},\ {\boldsymbol{w}},\ {\boldsymbol{d}},\ {\boldsymbol{\gamma }},\ {\boldsymbol{t}}$).
Input: graph ${\boldsymbol{G}}( {{\boldsymbol{V}},{\boldsymbol{E}}} )$
Window size ${\boldsymbol{w}}$
Embedding size ${\boldsymbol{d}}$
Walks per vertex ${\boldsymbol{\gamma }}$
Walk length ${\boldsymbol{t}}$
Output: matrix of vertex representations ${\boldsymbol{\Phi }} \in {{{\bf R}}^{| {{\bf V}} | \times {{\bf d}}}}$
1: Initialization: sample ${\boldsymbol{\Phi }}$ from ${{\boldsymbol{U}}^{| {\boldsymbol{V}} | \times {\boldsymbol{d}}}}$
2: Build a binary tree *T* from *V*
3: for *i* = 0 to ${\boldsymbol{\gamma }}$ do
4: ${\boldsymbol{O}}$ = Shuffle (${\boldsymbol{V}}$)
5: for each ${{\boldsymbol{v}}_{\boldsymbol{i}}} \in {\boldsymbol{O}}$ do
6: ${{\boldsymbol{W}}_{{{\boldsymbol{v}}_{\boldsymbol{i}}}}}{\boldsymbol{\ }}$= RandomWalk $( {{{\bf G}},\ {{{\bf v}}_{{\bf i}}},\ {{\bf t}}} )$
7: SkipGram $( {{\boldsymbol{\Phi }},\ {{\boldsymbol{W}}_{{{\boldsymbol{v}}_{\boldsymbol{i}}}}},{\boldsymbol{\ w}}} )$
8: end for
9: end for

The effects of parameters *w* and *t* on the results were not obvious. At the same time, smaller values can significantly reduce the experimental running time. Larger values of *w* and *t* may introduce additional noise and increase calculation burden. In fact, the structure of the MAN is totally different from those of previous benchmark datasets such as Facebook and Twitter. For traditional social networks, vertices with the same label are closely related. In the network of the present article, there are generally no edges between vertices of the same label, except in a protein–protein interaction network. The representation of vertices is mainly through the description of relationship with other types of vertices. To ensure as much experimental reproducibility as possible, we set the parameters *w* and *t* to the commonly used values 10 and 80. After generating the sequence of vertices, a Python package called gensim was applied to generate word-embedding representation.

The SkipGram algorithm is as follows:

**Table:** 

Algorithm 2: SkipGram $( {{\boldsymbol{\Phi }},\ {{\boldsymbol{W}}_{{{\boldsymbol{v}}_{\boldsymbol{i}}}}},{\boldsymbol{\ w}}} )$
1: for each ${{\boldsymbol{v}}_{\boldsymbol{j}}} \in {{\boldsymbol{W}}_{{{\boldsymbol{v}}_{\boldsymbol{i}}}}}$ do
2: for each ${{\boldsymbol{u}}_{\boldsymbol{k}}} \in {{\boldsymbol{W}}_{{{\boldsymbol{v}}_{\boldsymbol{i}}}}}[ {{\boldsymbol{j}} - {\boldsymbol{w}}:{\boldsymbol{j}} + {\boldsymbol{w}}} ]$ do
3: ${\boldsymbol{J\ }}( {\boldsymbol{\Phi }} ) = {\boldsymbol{\ }} - {\boldsymbol{\mathrm{log}Pr}}({{\boldsymbol{u}}_{\boldsymbol{k}}}|{\boldsymbol{\Phi }}( {{{\boldsymbol{v}}_{\boldsymbol{j}}}} ))$
4: ${\boldsymbol{\Phi }} = {\boldsymbol{\Phi }} - {{\bf \alpha }}*({\partial {{\bf J}}}/{\partial {\boldsymbol{\Phi }}})$
5: end for
6: end for

**Figure 5:**
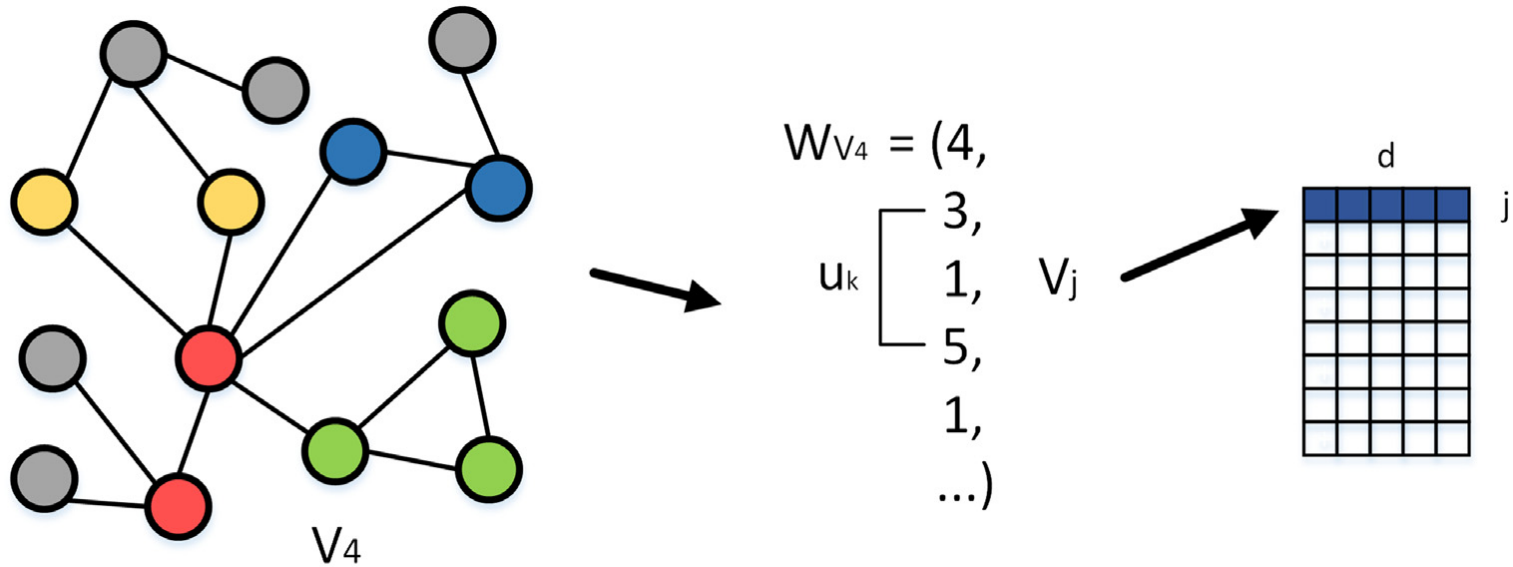
A visualization of DeepWalk. Vertex sequences can be obtained by random walks in the graph. Then, sequences are regarded as sentences, and vertexes as words. The SkipGram algorithm is used to obtain the embedding representation of the vertexes.

Note: whenever nodes are processed by DeepWalk, the test edges (relationships) in the network are stripped to ensure that the label information does not leak into the test set. A visualization of DeepWalk can be seen in Fig. 5.

### Stack autoencoder (SAE)

Attribute representation vectors of drugs and diseases comprise thousands of dimensions, and this is not helpful for classifier training. Stack autoencoder (SAE) is selected to map the vectors from the original space into low space, so as to reduce noise and feature dimensions. The autoencoder consists of 2 parts: the encoder, which maps the original input to the new space, and the decoder, which reconstructs the latent representation to the original input. For the original input *x*, the output *h*_1_ of the first hidden layer can be calculated by the following formula:

(8) $$\begin{eqnarray*}
{h_1} = {f_1}\ \left( {{W_1}x + {b_1}} \right),
\end{eqnarray*}$$

where *f*_1_ is the activation function, *W*_1_ is the weight matrix between the input layer and the first hidden layer, and *b*_1_ is the threshold of the first hidden layer neurons. Similarly, the output of each layer of the stack autoencoder can be calculated. The mean squared error between the output *y* and the original input *x* is:

(9) $$\begin{eqnarray*}
L\ = \left( {x,y} \right)\ = \mathop \sum \nolimits_i {\left( {{x_i} - {y_i}} \right)^2}\ .
\end{eqnarray*}$$

Then, the back-propagation algorithm is used to minimize the loss function to obtain the final model. We completed this task using the Python package Keras. The dimension of the hidden layer representation is 64, “MSE” is selected as the loss function, and the optimizer is “Adam.” The epochs and batch sizes are set to 10 and 128, respectively.

### Random forest classifier

Random forest is a classifier containing multiple decision trees whose output is determined by the mode of the output of each decision tree. It can efficiently process high-dimensional features, even in large data volumes. In addition, its high adaptability makes it possible to accept both discrete and continuous data. Here, we used the Python package sklearn to perform the random forest classifier, with default values.

## Results

### Relationship prediction based on the whole dataset under 5-fold cross-validation

Relationship prediction is common in both academia and industry. Here, some edges in the original graph are hidden as test sets and we construct the model based on the residual network. We evaluate the proposed method through 5-fold cross-validation. Under this strategy, the whole dataset is divided into 5 mutually exclusive subsets of roughly equal size. Each subset is used as the test set in turn to assess the effect of the classifier, and the remaining 4 subsets are used as a training set to construct the model. In each fold, areas under the receiver operating characteristic curves (ROC) and precision-recall curves (PR) are drawn to visualize the results, respectively. There are 114,150 valid experimental relationships in the whole network. In each fold cross-validation, 80% of the edges of the entire network are processed by Bioentity2vec and are treated as training samples; 20% of edges are considered test samples.

**Figure 6:**
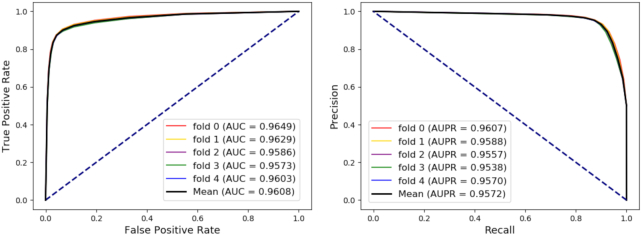
Performance obtained by the proposed method. Based on the whole network, the model achieved an area under the receiver operating characteristic curve (AUC) of 0.9608 and an area under the precision-recall curve (AUPR) of 0.9572 under 5-fold cross-validation.

Various evaluation criteria, including accuracy (Acc.), sensitivity (Sen.), specificity (Spec.), precision (Prec.), and Matthews correlation coefficient (MCC) are adopted to measure experimental results. Results are presented in Table 1 and Fig. 6 and show that our method can help to make stable and robust decisions and accurately discover potential associations.

**Table 1:** Results of accuracy (Acc.), sensitivity (Sen.), specificity (Spec.), precision (Prec.), and Matthews correlation coefficient (MCC) obtained under 5-fold cross-validation on the whole network

Fold	Acc. (%)	Sen. (%)	Spec. (%)	Prec. (%)	MCC (%)	AUC (%)
0	91.66	87.49	95.83	95.45	83.61	96.49
1	91.66	87.71	95.61	95.23	83.58	96.29
2	91.33	86.90	95.76	95.35	82.99	95.86
3	91.47	87.32	95.62	95.22	83.23	95.73
4	91.37	87.18	95.56	95.16	83.04	96.03
**Mean±SD**	**91.50 ± 0.16**	**87.32 ± 0.31**	**95.68 ± 0.11**	**95.28 ± 0.12**	**83.29 ± 0.29**	**96.08 ± 0.31**

### Feature importance comparison

**Figure 7:**
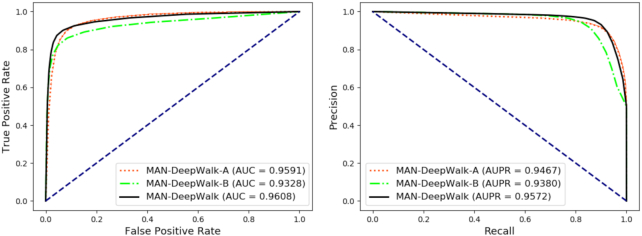
The receiver operating characteristic curves (ROCs), areas under the receiver operating characteristic curves (AUCs), precision-recall curves (PRs), and areas under the precision-recall curves (AUPRs) of the proposed method under 5-fold cross-validation. Representations of vectors combining attribute and behavior features are better than single types of information.

Nodes in a MAN can be represented as vectors by 2 types of information: node attribute and node behavior. To evaluate the effectiveness of these different kinds of feature, we compared the pure attribute-based method, pure behavior-based method, and a combination of these, based on various evaluation metrics: ROC, AUC, PR, and AUPR. Results are presented in Table 2 and Fig. 7 and show that the feature vector generated by combining the 2 kinds of information above provides more competitive performance.

**Table 2:** Results of accuracy (Acc.), sensitivity (Sen.), specificity (Spec.), precision (Prec.), and Matthews correlation coefficient (MCC) obtained by feature importance comparison experiments under 5-fold cross-validation on the whole network

Feature	Acc. (%)	Sen. (%)	Spec. (%)	Prec. (%)	MCC (%)	AUC (%)
Attribute	90.85 ± 0.09	89.79 ± 0.19	91.90 ± 0.11	91.73 ± 0.10	81.72 ± 0.17	95.91 ± 0.05
Behavior	88.67 ± 0.15	82.15 ± 0.24	95.19 ± 0.18	94.47 ± 0.19	78.00 ± 0.29	93.28 ± 0.13
**Both±SD**	**91.50 ± 0.16**	**87.32 ± 0.31**	**95.68 ± 0.11**	**95.28 ± 0.12**	**83.29 ± 0.29**	**96.08 ± 0.31**

Considering the “new sample” (cold start) problem in practical biological experiments, we do not guarantee that the degree of each node is >0. When only the sequences of the biological entities are known and their associations with other biomolecules are unknown, this strategy of constructing the vector by combining the node attribute and the node behavior can also predict potential relationships based on new sample and greatly improve the usability of the model.

### Comparison based on varying proportions of training sets

Data integrity is a top priority in achieving global relationship prediction. To explore the effects of missing data on the results, we separately learned the representation vectors of each node in the whole graph. We built models using varying proportions of edges and evaluated their performance.

Specifically, the dataset was divided into 4 parts: 20%, 40%, 60%, and 80% of the edges of the full graph as training samples. Correspondingly, the remaining edges of the graph, 80%, 60%, 40%, and 20%, were used as test samples. Here, each node is characterized only by its behavioral feature.

**Figure 8:**
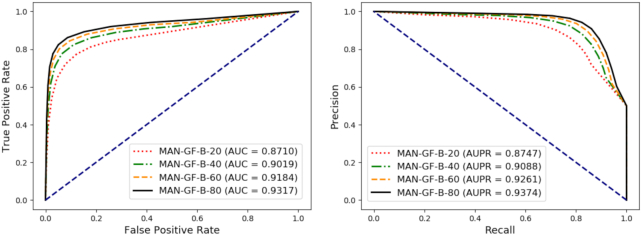
Performance comparison achieved by the proposed method, which was trained on different proportions of edges in the molecular association network.

It can be seen from Table 3 and Fig. 8 that, when only 20% of the edges of the entire network are used to generate node features and model construction, our method still achieves an AUC of 0.8710 and an AUPR of 0.8747. This demonstrates the excellent data-mining ability of this method.

**Table 3:** Results of accuracy (Acc.), sensitivity (Sen.), specificity (Spec.), precision (Prec.), and Matthews correlation coefficient (MCC) obtained trained and tested by different proportions of edges in the entire network

Proportion	Acc. (%)	Sen. (%)	Spec. (%)	Prec. (%)	MCC (%)	AUC (%)
20%	82.09	71.99	92.20	90.22	65.54	87.10
40%	85.54	77.48	93.61	92.38	72.03	90.19
60%	87.35	80.20	94.49	93.58	75.47	91.84
80%	88.64	82.35	94.92	94.19	77.89	93.17

### Additional experiment based on drug–disease association prediction

**Figure 9:**
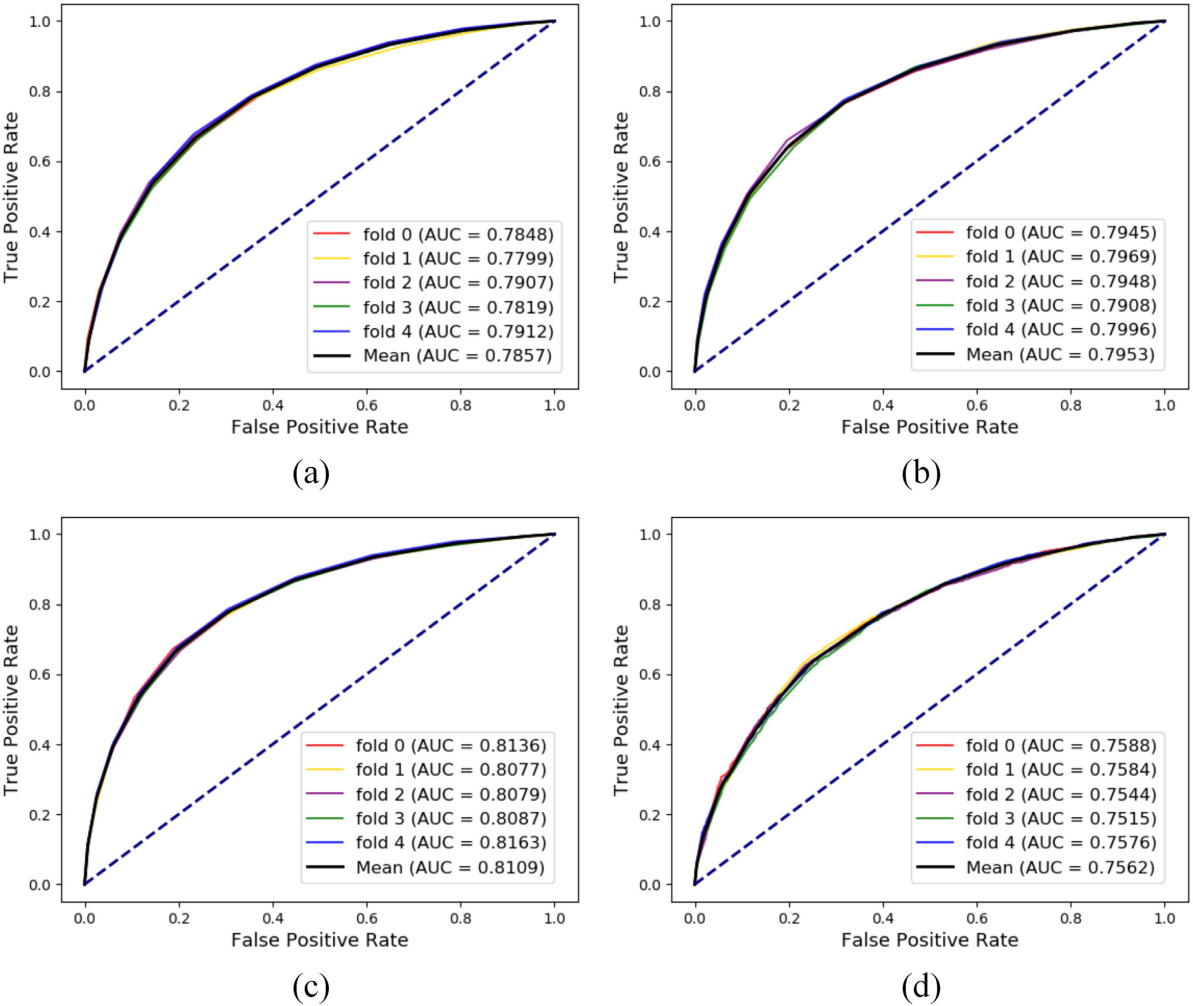
Comparison of receiver operating characteristic curves (ROCs), areas under the receiver operating characteristic curves (AUCs), precision-recall curves (PRs), and areas under the precision-recall curves (AUPRs) with 4 kinds of representation methods under 5-fold cross-validation based on a drug–disease association dataset.

Here, we take a specific example of drug–disease relationship prediction to carry out an additional experiment to evaluate the performance of our method, and compare it with the traditional single-function method. In total, 17,414 experimentally verified drug–disease associations were collected from the Comparative Toxicogenomics Database (CTD) [57]. Five-fold cross-validation was performed; ROCs and AUCs are shown in Fig. 9.

In Fig. 9a, the baseline for each node is represented as a 64-dimension vector by only its pure attributes, i.e., Morgan fingerprints or disease semantics.

For Fig. 9b, node behaviors are represented based on only drug–disease associations. Taking the idea of “guilt-by-association,” each node is abstracted into a 128-dimension vector by combining attributes and single-type associations. Compared to Fig. 9a, a slightly elevated AUC confirms the results of our feature importance comparison experiment and shows that measuring the local function of biomolecules improves prediction performance to some extent.

Figure 9c shows that we can consider the method proposed in this article as a kind of global embedding method. In each cross-validation, Bioentity2vec handles 80% drug–disease pairs with 17 kinds of relationships. Taking the 128-dimension vectors that integrate attributes and behaviors as inputs, the random forest classifier is chosen for training and testing. Compared with previous methods, the results that we obtained indicate that the extra edges serve as an intermediary to facilitate the prediction of associations when faced with specific problems.

For Fig. 9d, we carried out a special embedding strategy based on that described by Chen [65]. The remaining 17 types of relationship without drug–disease association pairs were learned by DeepWalk to obtain behavior representation vectors. This process does not depend on direct drug–disease associations. To eliminate the influence of the attribute feature on prediction performance, each node representation vector was constructed only by using behavior features under this special strategy. Nevertheless, the model still achieved a mean AUC of 0.7562 under 5-fold cross-validation, which implies that our MAN contains a wealth of biological information.

Note: to ensure the fairness of the experiment, negative samples of 4 experiments and each subset under 5-fold cross-validation were all consistent.

### A case study based on drug–disease association

A case study of ataxia was implemented to assess the performance of the proposed method in a real-world environment. As mentioned, we collected 17,414 drug–disease associations from CTD [57] and processed these as described by Zhang et al. [66]. To verify the prediction ability of the proposed model for new disease, we removed 61 association pairs related to ataxia. The remaining 17,353 drug–disease associations were used as a training set to generate features and construct the model. Ataxia is paired with each drug to form the test set. The top 10 results can be seen in Table 4. All association pairs were verified by CTD. Inference score and references were provided by CTD. The term “unconfirmed” refers to an association pair that we were not able to find in the CTD. We sorted all drugs by Direct Evidence Rank, and the top 10 results are presented in Table 4.

**Table 4:** The proposed method was applied to ataxia to predict potential disease-related drugs; 8 of the top 10 predicted drugs were confirmed in the CTD database.

No.	DrugBank ID	Evidence	CTD chemical name	Inference score	References	Direct evidence rank
1	db00313	CTD	Valproic acid	32.61	22	263
2	db00252	CTD	Phenytoin	3.04	32	50
3	db00635	CTD	Prednisone	null	1	178
4	db00563	CTD	Methotrexate	6.89	8	8
5	db00544	CTD	Fluorouracil	3.12	5	46
6	db00907	CTD	Cocaine	4.94	7	18
7	db00477	CTD	Chlorpromazine	3.79	2	31
8	db01577	CTD	Metamfetamine	Unconfirmed	Unconfirmed	Unconfirmed
9	db00661	CTD	Verapamil	Null	2	205
10	db00363	CTD	Unconfirmed	Unconfirmed	Unconfirmed	Unconfirmed

Such prediction results can be attributed to the following 2 points: (i) in an open environment, there are many problems associated with new samples (cold start). These samples can only be represented by attributes because there are not enough known relationships. (ii) CTD and DrugBank are 2 different databases, and their differences lead to insufficient relationships to generate expressive behavior representations of abiotic entities.

## Conclusion

Current biological entity relationship calculation methods only focus on a single type of relationship and cannot simultaneously detect complex multi-type relationships between bioentities. The model proposed here may solve this issue. Specifically, in developing a comprehensive molecular association network, we propose the use of Bioentity2vec to generate representation vectors for different bioentities. Combined with the random forest classifier, promising results have been demonstrated in single- and multi-type relationship prediction. Our research represents a preliminary exploration from isolated molecules to complex molecular association networks. The concepts expressed in our research may yield novel ideas for the development of new theoretical systems, expand research objects, and accelerate the integration of proteomics and genomics.

## Availability of Supporting Source Code and Requirements

Project name: Bioentity2vec

Project home page: https://github.com/CocoGzh/Bioentity2vec

Operating systems: Windows

Programming language: Python 3.7

Other requirements: Anaconda3, Open-NE

License: MIT

RRID :SCR_018179

## Availability of Supporting Data and Materials

All source code and supporting data are available in the *GigaScience* GigaDB database [67] and GitHub [68].

## Abbreviations

Acc: accuracy; AUC: area under receiver operating characteristic curve; AUPR: area under precision-recall curve; circRNA: circular RNA; CTD: Comparative Toxicogenomics Database; DAG: directed acyclic graph; lncRNA: long noncoding RNA; MAN: molecular association network; MCC: Matthews correlation coefficient; MeSH: Medical Subject Heading; mRNA: messenger RNA; miRNA: microRNA; NCBI: National Center for Biotechnology Information; ncRNA: noncoding RNA; PR: precision-recall curve; Prec: precision; ROC: receiver operating characteristic curve; SAE: stack autoencoder; Sen: sensitivity; SMILES: simplified molecular input line entry specification; Spec: specificity; SD: standard deviation.

## Competing Interests

The authors declare that they have no competing interests.

## Funding

This work was supported by a grant from the National Key R&D Program of China (grant No. 2018YFA0902600) and grants from the National Science Foundation of China (grant Nos. 61722212, 61861146002, 61732012, and 61902342).

## Authors’ Contributions

Z.-H.G. and Z.-H.Y. considered the algorithm, arranged the datasets, and performed the analyses. Y.-B.W., D.-S.H., H.-C.Y. and Z-H.C. wrote the manuscript. All authors read and approved the final manuscript.

## Supplementary Material

giaa032_GIGA-D-19-00385_Original_Submission

giaa032_GIGA-D-19-00385_Revision_1

giaa032_GIGA-D-19-00385_Revision_2

giaa032_GIGA-D-19-00385_Revision_3

giaa032_Response_to_Reviewer_Comments_Original_Submission

giaa032_Response_to_Reviewer_Comments_Revision_1

giaa032_Response_to_Reviewer_Comments_Revision_2

giaa032_Reviewer_1_Report_Original_Submission Emre Guney -- 12/15/2019 Reviewed

giaa032_Reviewer_1_Report_Revision_1 Emre Guney -- 1/24/2020 Reviewed

giaa032_Reviewer_2_Report_Original_Submission Alper Ozcan -- 12/31/2019 Reviewed

giaa032_Reviewer_2_Report_Revision_1 Alper Ozcan -- 1/24/2020 Reviewed
