# Supplementary material for: Bioentity2vec: Attribute- and behavior-driven representation for predicting multi-type relationships between bioentities
Source: Gigascience. 2020 Jun 13;9(6):giaa032. doi: 10.1093/gigascience/giaa032 (PMC7293023; doi:10.1093/gigascience/giaa032)
Supplement: giaa032_GIGA-D-19-00385_Revision_2 [file giaa032_giga-d-19-00385_revision_2.pdf]

GigaScience

Bioentity2vec: Attribute- and Behavior-driven Representation for Multi-type Relationship Prediction between Various Bioentities

--Manuscript Draft--

**Manuscript Number:**	GIGA-D-19-00385R2
**Full Title:**	Bioentity2vec: Attribute- and Behavior-driven Representation for Multi-type Relationship Prediction between Various Bioentities
**Article Type:**	Research
**Funding Information:**	NSFC Excellent Young Scholars Program (61722212) — Dr. zhuhong you<br>National Natural Science Foundation of China (61902342) — Mr. Yan-Bin Wang<br>International Cooperation and Exchange Programme (61861146002) — Dr. De-Shuang Huang<br>Key Programme (61732012) — Dr. De-Shuang Huang<br>Key Technologies Research and Development Program (2018YFA0902600) — Dr. De-Shuang Huang
**Abstract:**	The explosive growth of genomic, chemical and pathological data provides new opportunities and challenges for humans to reexamine life activities in cells. However, there exist few computational models that aggregate various bioentities to comprehensively reveal the physical and functional landscape of biological system. Here, we construct a graph called Molecular Association Network (MAN) and a representation method called Bioentity2vec. Specifically, MAN is a heterogeneous attribute network consists of 18 kinds of edges (relationships) among 8 kinds of nodes (bioentities). Bioentity2vec is an algorithm that represents the nodes as vectors by integrating bioentity attribute such as RNA sequence and bioentity behavior that is the relationship between bioentities. Then, random forest classifier is applied to carry out the relationship prediction task. The proposed approach achieved promising performance on 18 relationships, with AUC of 0.9608 and AUPR of 0.9572. The results strongly prove that MAN is a network with rich topological and biological information and Bioentity2vec can adequately characterize bioentities. Generally, our method can achieve simultaneous prediction of both single-type and multi-type relationships, which bring beneficial inspiration to relevant scholars and expand the medical research paradigm.
**Corresponding Author:**	zhuhong you<br><br>CHINA
**Corresponding Author Secondary Information:**	
**Corresponding Author's Institution:**	
**Corresponding Author's Secondary Institution:**	
**First Author:**	Zhen-Hao Guo
**First Author Secondary Information:**	
**Order of Authors:**	Zhen-Hao Guo<br>zhuhong you<br>De-Shuang Huang<br>Hai-Cheng Yi<br>Yan-Bin Wang

Powered by Editorial Manager® and ProduXion Manager® from Aries Systems Corporation

	Zhan-Heng Chen
**Order of Authors Secondary Information:**	
**Response to Reviewers:**	Authors' Response to Reviewers' Comments<br>Paper title:Biomarker2vec: Attribute- and Behavior-driven Representation for Multi-type Relationship Prediction between Various Biomarkers<br>Manuscript ID:GIGA-D-19-00385R1<br>Authors:Zhen-Hao Guo; Zhu-Hong You; De-Shuang Huang; Hai-Cheng Yi; Yan-Bin Wang; Zhan-Heng Chen<br><br>We are grateful to the editor and reviewers for putting in efforts to review the paper with the aim of improving the quality of our paper. We have addressed the concerns of the editor and reviewers in the revised manuscript. In particular, the following revisions have been made.<br><br>Reviewer 1<br>I thank authors for addressing my comments. Few additional points:<br>Response: Thank to the reviewer for this positive comment.<br>Comment 1:<br>1. Fig 2, entities are still referred as biomarker. Additionally, what the dashed borders / lines mean should be specified.<br>Response: Thanks for this useful comment.<br>According to the suggestions of the reviewer, we corrected the picture and added a description of the dashed borders / lines.<br><br>Comment 2:<br>2. Answer to my previous question #4 (optimization of parameters, usage of gensim) should be included/discussed in the text.<br>Response: Thanks for this useful comment.<br>The response has been discussed and highlighted in blue in the revised manuscript.<br><br>Comment 3:<br>3. To demonstrate the practical utility of the predictions, Table 4 should include the name and ranking of the drug as per the known drugs in CTD in addition to the inference score and number of references (as commented in #12, the http://ctdbase.org/detail.go?type=disease&acc=MESH%3aD001259&view=chem). It would also be beneficial, what is the accuracy of their method on predicting the top 10,20,50,100 drugs in CTD (as well as a relevant discussion on these results).<br>Response: Thanks for this useful comment.<br>We have converted the IDs of the drugs in DrugBank to the CTD chemical names and added them to Table 4. We sorted all drugs by Direct Evidence, and the results of top-10 were added to Table 4.<br>Considering that many top drugs in CTD do not appear in the network we construct, the prediction of these drugs actually uses only the attributes of the drug, that is, the molecular structure of the drug. Therefore, it is difficult to reflect the advantages of our proposed method. We add these discussions in the reviewed manuscript and highlight them in blue.<br><br>Again, we appreciate all of your insightful comments. We hope that we have addressed your concerns satisfactorily. Thank you for taking you time and energy to help us improve the paper.<br><br>Reviewer 2<br>The methods are appropriate to the aims of the study, they are well described, and necessary controls are included. The conclusions are adequately supported by the data shown. The quality of language in the manuscript is clear.<br>Response: Thank you very much for helpful comments.<br><br>Comment 1:<br>The statistical tests used in manuscript is not appropriate. t-test or any other related statistical test is used in order to compare the proposed model with baseline.<br>Response: Thanks for this useful suggestion.<br>As the reviewer said, t-test is an evaluation method widely used in bioinformatics. T-test or any other related statistical test is the more acceptable result. However, the

	proposed method is a deep learning-based representation algorithm and machine learning-based prediction model. Therefore, we prefer to use computer science evaluation methods. Our follow-up work will complement and improve it.<br><br>Again, we appreciate all of your insightful comments. We hope that we have addressed your concerns satisfactorily. Thank you for taking you time and energy to help us improve the paper.
**Additional Information:**	
**Question**	**Response**
Are you submitting this manuscript to a special series or article collection?	No
**Experimental design and statistics**<br><br>Full details of the experimental design and statistical methods used should be given in the Methods section, as detailed in our Minimum Standards Reporting Checklist. Information essential to interpreting the data presented should be made available in the figure legends.<br><br>Have you included all the information requested in your manuscript?	Yes
**Resources**<br><br>A description of all resources used, including antibodies, cell lines, animals and software tools, with enough information to allow them to be uniquely identified, should be included in the Methods section. Authors are strongly encouraged to cite Research Resource Identifiers (RRIDs) for antibodies, model organisms and tools, where possible.<br><br>Have you included the information requested as detailed in our Minimum Standards Reporting Checklist?	Yes
**Availability of data and materials**<br><br>All datasets and code on which the conclusions of the paper rely must be either included in your submission or deposited in publicly available repositories	Yes

(where available and ethically appropriate), referencing such data using a unique identifier in the references and in the “Availability of Data and Materials” section of your manuscript.<br><br>Have you have met the above requirement as detailed in our Minimum Standards Reporting Checklist?	

Powered by Editorial Manager® and ProduXion Manager® from Aries Systems Corporation

Manuscript

Click here to access/download;Manuscript;Revised_manuscript_GIGA-D-

Bioentity2vec: Attribute- and Behavior-driven Representation for Multi-type Relationship Prediction between Various Bioentities

Zhen-Hao Guo [1,2,†], Zhu-Hong You [1,2,†,*], De-Shuang Huang [3], Hai-Cheng Yi [1,2], Yan-Bin Wang [4], Zhan-Heng Chen [1,2]

1 The Xinjiang Technical Institute of Physics and Chemistry, Chinese Academy of Sciences, Urumqi 830011, China; guozhenhao17@mails.ucas.ac.cn (Z-H. G.); yihaicheng17@mails.ucas.ac.cn (H-C. Y.)
2 University of Chinese Academy of Sciences, Beijing 100049, China
3 Computer Science Department, Tongji University, Shanghai 200000, China; dshuang@tongji.edu.cn (D-S. H.)
4 School of Cyber Science and Technology, Zhejiang University, Hangzhou 310000, Zhejiang
† These authors contributed equally to this work.
* Correspondence: zhuhongyou@ms.xjb.ac.cn (Z-H. Y.), wangyanbin15@mails.ucas.ac.cn (Y.-B.W)

Abstract

The explosive growth of genomic, chemical and pathological data provides new opportunities and challenges for humans to reexamine life activities in cells. However, there exist few computational models that aggregate various bioentities to comprehensively reveal the physical and functional landscape of biological system. Here, we construct a graph called Molecular Association Network (MAN) and a representation method called Bioentity2vec. Specifically, MAN is a heterogeneous attribute network consists of 18 kinds of edges (relationships) among 8 kinds of nodes (bioentities). Bioentity2vec is an algorithm that represents the nodes as vectors by integrating bioentity attribute such as RNA sequence and bioentity behavior that is the relationship between bioentities. Then, random forest classifier is applied to carry out the relationship prediction task. The proposed approach achieved promising performance on 18 relationships, with AUC of 0.9608 and AUPR of 0.9572. The results strongly prove that MAN is a network with rich topological and biological information and Bioentity2vec can adequately characterize bioentities. Generally, our method can achieve simultaneous prediction of both single-type and multi-type relationships, which bring beneficial inspiration to relevant scholars and expand the medical research paradigm.

Keywords: Network biology; System biology; Bioentity2vec; Multi-type relationship prediction.

Source code and data are available at https://github.com/CocoGzh/Bioentity2vec.

Introduction

A key task in the post-genomic era is to systematically and comprehensively understand the relationships between bioentities in living cells [1]. Rapidly developing high-throughput technologies and the discoveries of new transcripts or translations provide foundation for this mission [2]. For example, the increasing evidence prove that the biomolecule networks such as protein-protein interaction network, ncRNA-disease association network, drug-target interaction network play significant roles in protein synthesis [3], gene expression [4], RNA processing [5] and developmental regulation [6], etc. Consequently, research on relationships between bioentities not only opens novel insights to understand life process, but also facilitates to disease prevention, diagnosis, treatment and drug development.

Identifying the relationships in large-scale data via wet experiments is labor-intensive, time-consuming and can only meet limited requirements in real-world demands. Meanwhile, the extensive accumulated experiment data lead to the information overload which results in excessing cost of acquiring valuable knowledge. Hence, it is urgent to design automatic computational tools to provide assistance and guidance for practice [7].

In fact, prediction models based on validated evidence to discover potential relationships have been widely developed and heavily applied. Guo *et al.* proposed a learning-based model to predict potential lncRNA-disease associations by integrating known association evidence, disease semantic similarity [8]. Wang *et al.* carry out the Logistic Model Tree to discover unknown miRNA-disease associations by integrating multi-source information [9]. Li *et al.* used the Position-Specific Scoring Matrix (PSSM) to represent proteins and then put them into an ensemble classifier to predict self-interacting and non-self-interacting proteins [10]. Wang *et al.* utilize the Rotation Forest as a classifier to uncover unknown drug-target interactions by drug structure and protein sequence [11].

Many attempts have been made to detect the uncovered relationships through various methods including matrix factorization [12], machine learning [13] and network analysis [14]. However, the incompleteness of the data constrains the credibility of the prediction results accompanied with higher False Positive Rate (FPR) and False Negative Rate (FNR) [15]. In recent years, the discovery of new types bioentities and relationships provides novel insights to improve this situation to some extent. Additional bioentities are attempted to be considered as bridges, and synergistically help the grasp of underlying biological principles to improve the prediction effect. Chen *et al.* regarded environmental factors as a medium and effectively improved the prediction effect of miRNA-disease association [16]. Cui *et al.* made a preliminary exploration in drug-disease association prediction which drawn support from gene expression data [17].

Tremendous advances have been made in molecular biology over the past few years, yet the development of computational models is still in infancy. The major limitation of all

above methods is that none of them regard a cell as a complete unit, even if these methods have their own unique advantages. In fact, cells are composed of nodes (Bioentities) and edges (relationships) like a network (graph) to maintain normal life activities and physiological functions. Ideally, establishing connections from internal or external factors to expression would be rewarding in understanding the landscape of biology system. In this paper, a network called Molecular Association Network (MAN) is constructed based on various online databases such as NONCODE [18] and miRbase [19] to provide a platform to help systematically analyze the relationships between Bioentities within human cells.

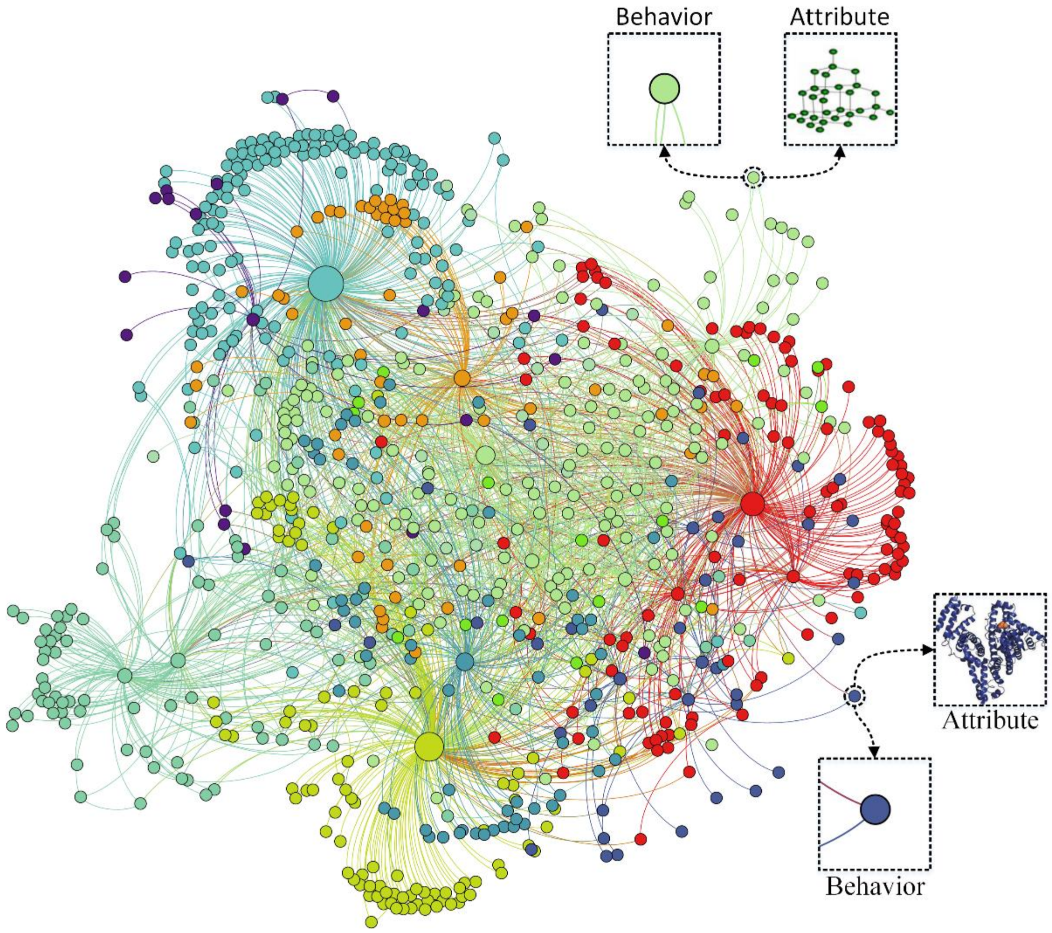

Figure 1. An example of visualization based on MAN, where different colors represent different types of bioentities. Each bioentity contains 2 kinds of information including node behavior (relationships with other nodes) and node attribute (sequences of protein or RNA, chemical structure of drug, and semantics of disease and microbe).

Faced with such a large-scale network, the most critical challenge is how to quickly and effectively describe the nodes in this network. In general, each bioentity can be defined by its own attributes and behavior [20]. Attribute feature can be represented by RNA sequence, drug structure and etc [21-23]. The human semantic description of drug or disease can also be treated as a kind of representation and widely used in relationship prediction tasks such as drug reposition [23]. On the other hand, network-based methods, especially the rapid development of graph embedding (network representation)

algorithms has shown us great hope for clearly describing the relationship between nodes [24-31].

Graph embedding which aims to represent nodes in the network as low-dimensional, dense vector forms is chosen to respond to this situation [32]. Although some existing models in bioinformatics contain the idea of graph embedding, many of them still focus on traditional techniques including Principal Component Analysis (PCA) [33], Multidimensional scaling (MDS) [34], Isomap [35] and Local Linear Embeddings (LLE) [36]. In general, these methods offer satisfactory performance on small networks. However, at least quadratic time complexity restricts the application of these methods to large-scale data. The recent remarkable performance of deep learning has attracted extensive research attention. Here, the representation method called DeepWalk is applied to conduct this task.

In this paper, a network called MAN is constructed and a graph embedding algorithm is proposed to represent each node as a vector. Then random forest is applied as the classifier to carry out the relationship prediction task. Specifically, 18 kinds of associations or interactions among 8 kinds of biomolecules are collected from various databases to construct the network. Then the lower triangular part of the adjacency matrix called A is kept to simplify calculation and storage. Each bioentity can be represented as a vector by combining attribute and behavior feature. The flowchart can be seen in Figure 2. The proposed method obtained AUC of 0.9608 and AUPR of 0.9572 under 5-fold cross validation on the multi-type relationship prediction task of whole network. Furthermore, we implemented 3 comparison experiments including feature importance comparison, embedding strategy comparison and proportions of training set comparison. The remarkable performance demonstrated that MAN with bright prospects of revealing uncovered relationships. We hope that this work can provide assistance and guidance for wet experiments, and be a useful inspiration for researchers to understand gene regulation, disease mechanism and discovery of new drugs at molecular level.

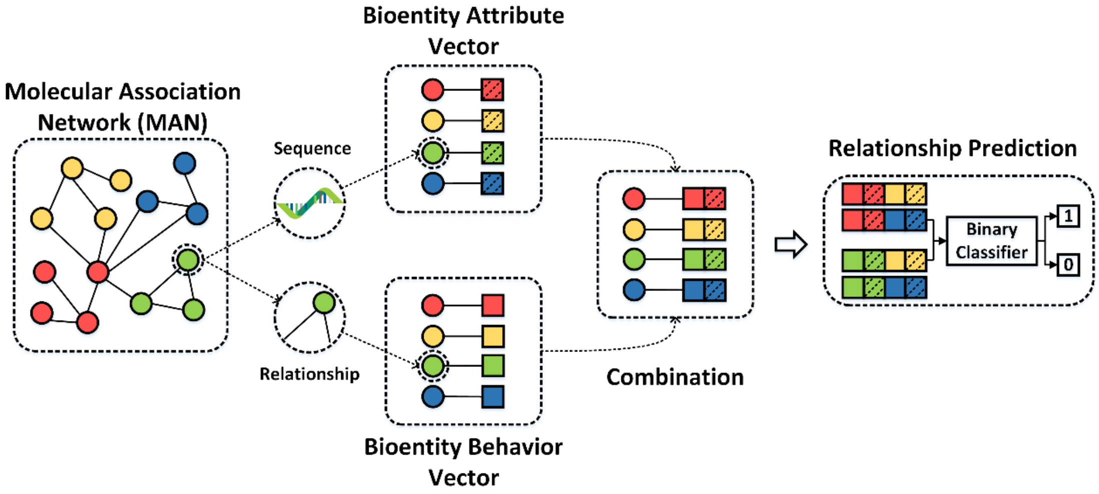

Figure 2. The flowchart of the proposed method. Each node in the network can be described from 2 perspectives, one is the attribute feature such as sequence and chemical structure that can be learned as a 64-dimension vector by k-mer and etc. methods, the other is the behavior feature which can be represented as a 64-dimension vector through DeepWalk. Attribute and behavior feature are distinguished by dashed and unprocessed

squares. After combining above 2 information, each node can be represented as a 128-dimension vector. The positive samples are experimentally verified relationships and the negative samples are the same number of unlabeled relationships which are randomly selected in matrix A. Taking the low-dimensional dense vectors as input, random forest is used to carry out the prediction task.

Materials and Methods

2.1 Construction of the MAN

To construct the MAN comprehensively, 18 different kinds of experimentally verified associations or interactions are collected from various databases [37-56]. After unifying identifier, we obtained a total of 8 diverse types of bioentities. Then, all relationships and bioentities are aggregated together to form the MAN. The specific quantity and proportion of each type of bioentities or relationships are shown in the Figure 3.

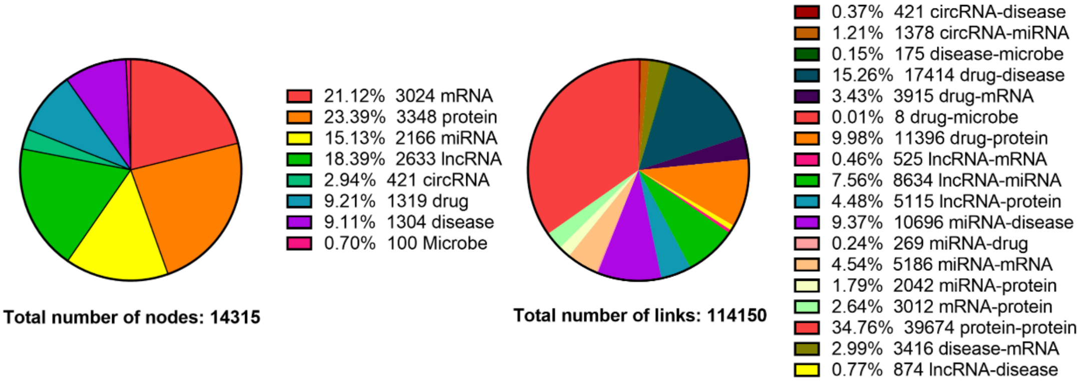

Figure 3. The details of different biomolecules and relationships

2.2 Node attribute representation: K-mer, Semantics and Fingerprint

It is obvious that the intrinsic attribute such as the sequence of protein and RNA, the semantics of disease and microbe, and the chemical structure of drug is the essential feature of each biomolecule. The details of how they are represented as vectors are as follows.

For protein, mRNA, miRNA, lncRNA and circRNA, their sequences are collected from STRING [55], NCBI [57], miRBase [19], NONCODE [18] and circBase [58] respectively. Given that proteins are composed of 20 different types of amino acids and inspired by the method of Shen *et al.* [59], we first classify them into 4 categories based on the polarity of the amino acid side chains including (Ala, Val, Leu, Ile, Met, Phe, Trp, Pro), (Gly, Ser, Thr, Cys, Asn, Gln, Tyr), (Arg, Lys, His) and (Asp, Glu). RNA including mRNA, miRNA, lncRNA and circRNA are composed of 4 kinds of nucleotides including Adenine (A), Guanine (G), Cytosine (C) and Uracil (U) with the same sequence composition, so we directly encode their original sequence without any pretreatment. Each RNA or Protein can be represented as a vector by k-mer, in which all dimensions represent the full permutation of k nucleotide (or amino acid) combination and the value of each dimension is the normalized frequency of the corresponding k-mer appearing in the sequence. In this

paper, k is set to 3 and each protein or RNA can be represented as a 64-dimension ($4^3 = 4 \times 4 \times 4$) vector.

For disease and microbe, their Medical Subject Headings (MeSH) descriptors which are comprehensive control vocabularies organized by U.S. National Library of Medicine are downloaded from https://www.nlm.nih.gov/. The top-level categories in the MeSH Tree Structure are: Anatomy [A], Organisms [B], Diseases [C] and so on. The categories corresponding to microbes and diseases are B and C, respectively. Inspired by Wang *et al.* [22], we construct the Directed Acyclic Graph (DAG) of disease and microbe to represent them through their semantics. For example, a microbe M can be represented as a graph $DAG(M) = (M, N(M), E(M))$ where $N(M)$ is the set of all nodes in M's DAG and $E(M)$ is the set of all edges in M's DAG. The semantic contribution of microbe m which is in the node set $N(M)$ to M can be defined as:

$$\begin{cases} V_M(m) = 1 & if\ m = M \\ V_M(m) = max\{\Delta * V(m')|m' \in children\ of\ m\} & if\ m \neq M \end{cases} \quad (1)$$

where Δ denotes an attenuation factor and is defined as 0.5 according to previous literature [22]. In the DAG generated by microbe M, M's contribution to itself can be regarded as the maximum and equals to 1, and the remaining diseases will contribute less and less to M as the distance increases. Therefore, the sum of the contributions of microbes which are in the set $N(M)$ to M can be calculated as follows:

$$SV(M) = \Sigma_{m \in N(M)} V_M(m) \quad (2)$$

Then the similarity between microbe i and j can be calculated by the following formula：

$$Similarity(i, j) = \frac{\Sigma_{m \in N(i) \cap N(j)}(V_i(m) + V_j(m))}{SV(i) + SV(j)} \quad (3)$$

The node attribute of microbe or disease can be represented by semantics similarity, which is converted into a 64-dimensional vector after feature extraction and transformation by the stack autoencoder. A DAG example of microbe Staphylococcus is as follows:

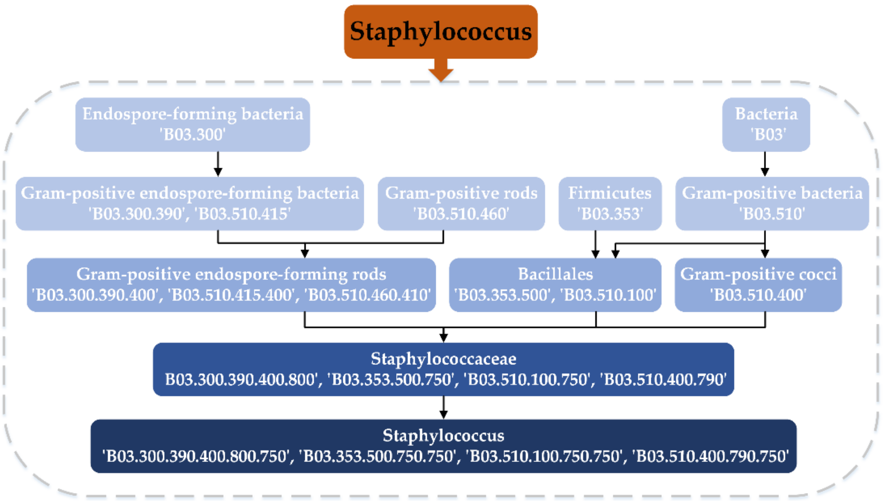

Figure 4. Construction the DAG of Staphylococcus. The father node of the current microbe can be obtained by deleting the last three digits of the descriptor. For example, for Bacillales (B03.353.500, B03.510.100), we can remove the last three digits to get Firmicutes (B03.353) and Gram-positive bacteria (B03.510).

For drug, we download their SMILES [60] from DrugBank [46] and transform the SMILES into corresponding Morgan Molecular Fingerprints [61] by python package called RDKit [62]. To unify dimensions and improve feature quality, stack autoencoder is used to convert each original molecular fingerprint into a 64-dimensional vector.

2.3 Node behavior representation: DeepWalk

Inspired by the idea of "guilt-by-association" assumption, we come up with a more general feature in complex networks, that is, the behavior feature of bioentity. Generally speaking, it is a kind of embedding representation of the known edges between nodes in the network. Despite a row or column of the adjacency matrix can directly be utilized as a representation vector for node behavior in one-hot encoding method. However, there is no concept of similarity between each dimension of such high-dimensional, sparse vectors, as it is represented as indices in a relationship. Meanwhile, the one-hot encoding method takes up a lot of storage space and is not conducive to the input of downstream tasks. Hence, how to extract the behavior information of node from the complex network such as MAN is a formidable challenge.

In this paper, a network embedding method called DeepWalk which first applies the technique of natural language processing in deep learning for node representation is adopted to undertake this task [63]. The main idea is to obtain a certain length of the walk sequence through Random Walk, an ideal mathematical state of Brownian motion that can repeatedly access the visited nodes. After obtaining enough sequences, the vectors of the nodes can be learned by the skip-gram model. The direct analog is to estimate the likelihood of observing vertex v_i given all the previous vertices visited so far in the random walk, *i.e.*

$$P_r\left(v_i | (v_1, v_2, \dots, v_{i-1})\right) \tag{4}$$

The goal is to learn a latent representation and the mapping function is:

$$\Phi: v \in V \mapsto R^{|V| \times d} \tag{5}$$

The problem then, is to estimate the likelihood:

$$P_r\left(v_i | (\Phi(v_1), \Phi(v_2), \dots, \Phi(v_{i-1}))\right) \tag{6}$$

The recent relaxation in language modeling turns the prediction problem and this yields the optimization problem:

$$\underset{\Phi}{minimize} = -logP_r(\{v_{i-w}, \dots, v_{i+w}\} \backslash v_i | \Phi(v_i)) \tag{7}$$

The main steps of the algorithm are as follows:

Algorithm 1: DeepWalk ($\boldsymbol{G}, \boldsymbol{w}, \boldsymbol{d}, \boldsymbol{\gamma}, \boldsymbol{t}$).

Input: graph $\boldsymbol{G}(\boldsymbol{V}, \boldsymbol{E})$
    window size $\boldsymbol{w}$
    embedding size $\boldsymbol{d}$
    walks per vertex $\boldsymbol{\gamma}$
    walk length $\boldsymbol{t}$
Output: matrix of vertex representations $\boldsymbol{\Phi} \in \mathbf{R}^{|\mathbf{V}| \times \mathbf{d}}$
1: Initialization: Sample $\boldsymbol{\Phi}$ from $\boldsymbol{U}^{|\boldsymbol{V}| \times \boldsymbol{d}}$
2: Build a binary Tree T from V
3: **for** i =0 to $\boldsymbol{\gamma}$ **do**

O = Shuffle (V)

for each $v_i \in O$ do

  W_{v_i} = RandomWalk ($\mathbf{G}, \mathbf{v_i}, \mathbf{t}$)

  SkipGram (Φ, W_{v_i}, w)

end for

end for

We found that the effect of parameter w and t on the results is not obvious. At the same time, a smaller value of them can significantly reduce the running time of the experiment. Larger w and t may introduce additional noise and increase calculation burden. In fact, the structure of MAN is totally different from previous benchmark datasets such as Facebook, twitter, etc. For traditional social networks, vertices of the same label are closely related. In the network of this manuscript, there are generally no edges between vertices of the same label except for protein-protein interaction network. The representation of vertices is mainly through the description of relationship with other types of vertices. In order to ensure the experimental reproducibility as much as possible, we set the parameters w and t to the commonly used values 10 and 80. After generating the sequence of vertices, python package called gensim was applied to generate the word embedding representation.

The SkipGram algorithm is as follows:

Algorithm 2: SkipGram (Φ, W_{v_i}, w)

for each $v_j \in W_{v_i}$ do

  for each $u_k \in W_{v_i}[j-w:j+w]$ do

    $J(\Phi) = -log Pr(u_k | \Phi(v_j))$

    $\Phi = \Phi - \alpha * \frac{\partial J}{\partial \Phi}$

  end for

end for

Note whenever the nodes are processed by DeepWalk, the test edges (relationships) in the network are stripped to ensure that the label information is not leaked into the test set. The visualization of DeepWalk can be seen in Figure 5.

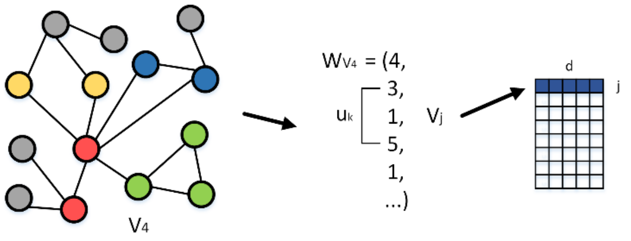

Figure 5. A visualization of DeepWalk. Vertex sequence can be obtained by random walks in the graph. Then the sequences are regarded as sentences and Vertexes as words. The SkipGram algorithm is used to obtain the embedding representation of the Vertexes.

2.4 Stack Autoencoder (SAE)

Autoencoder and its variants have been widely used in unsupervised feature learning

and classification tasks. Considering that the attribute representation vectors of drug and disease consisting of thousands of dimensions which is unfriendly to classifier training. The Stack Autoencoder (SAE) is selected to map vectors of the original space into the new space to reduce noise and make features easy to distinguish. The autoencoder consists of two parts, one is the encoder that maps the original input to the new space, and the other is the decoder that reconstructs the latent representation in the new space back to the original input. For the original input x, the output h_1 of the first hidden layer can be calculated by the following formula:

$$h_1 = f_1(W_1 x + b_1) \tag{8}$$

Where f_1 is the activation function, W_1 is the weight matrix between the input layer and the first hidden layer, and b_1 is the threshold of the first hidden layer neurons. Similarly, the output of each layer of the stack autoencoder can be calculated. The mean squared error between the output y and the original input x is:

$$L = (x, y) = \sum_i (x_i - y_i)^2 \tag{9}$$

Then the back-propagation algorithm is used to minimize the loss function to get the final model. We completed this task by using a python packge called Keras lab this step. The dimension of the hidden layer representation is 64. 'MSE' is selected as the loss function and the optimizer is 'Adam'. The epochs and batch sizes are set to 10 and 128, respectively.

2.5 Random Forest Classifier

Random Forest is a classifier that contains multiple decision trees whose output is determined by the mode of the output of each decision tree. It can process high-dimensional features efficiently even in large data volumes. In addition, its high adaptability makes it possible to accept both discrete and continuous data. In this paper, we performed the random forest classifier by a python package called sklearn and all the hyperparameters such as n_estimators and etc. are set to the default value.

Results

3.1. Relationship prediction based on the whole dataset under 5-fold cross validation

Relationship prediction is a common task in both academia and industry. Here, we will hide a set of edges of the original graph and construct the model based on the incomplete network. Then the hidden edges are utilized for test to assess the proposed method. 5-fold cross validation which is widely used evaluation strategy is applied to carry out this task. In 5-fold cross validation, the whole dataset is divided into 5 mutually exclusive subsets of roughly equal size. Each subset is used as the test set in turn to assess the effect of the classifier, and the remaining 4 subsets are utilized as training set to construct the model. In each fold, area under Receiver Operating Characteristic Curve (ROC) and Precision-Recall Curve (PR) are drawn to visualize the results, respectively. There are total 114,150 experimental valid relationships in the whole network. In each fold

cross-validation, 80% edges of the entire network are processed by Bioentity2vec and are treated as the training samples, 20% edges are treated as the test samples.

At the same time, a wide range of evaluation criteria including accuracy (Acc.), sensitivity (Sen.), specificity (Spec.), precision (Prec.) and matthews correlation coefficient (MCC) are adopted to comprehensively and fairly estimate the propose method. The details of results are shown in the Table 1 and Figure 6. Competitive performance under various evaluation criteria demonstrates the keen ability to discover potential associations. The relatively low variance implies the superior robustness and stability of MAN in different situations.

Table 1. Results of accuracy (Acc.), sensitivity (Sen.), specificity (Spec.), precision (Prec.) and MCC obtained under 5-fold cross validation on the whole network.

fold	Acc. (%)	Sen. (%)	Spec. (%)	Prec. (%)	MCC (%)	AUC (%)
0	91.66	87.49	95.83	95.45	83.61	96.49
1	91.66	87.71	95.61	95.23	83.58	96.29
2	91.33	86.9	95.76	95.35	82.99	95.86
3	91.47	87.32	95.62	95.22	83.23	95.73
4	91.37	87.18	95.56	95.16	83.04	96.03
**Average**	**91.5±0.16**	**87.32±0.31**	**95.68±0.11**	**95.28±0.12**	**83.29±0.29**	**96.08±0.31**

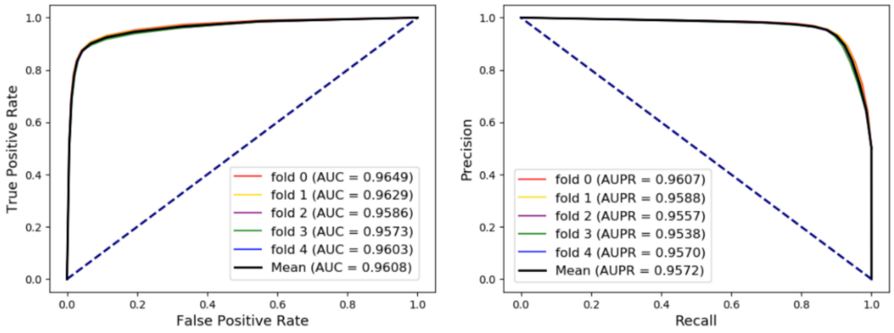

Figure 6. The ROCs, AUCs, PRs and AUPRs obtained under 5-fold cross validation on the whole network.

3.2. Feature importance comparison

Each node in MAN can be represented as vectors by 2 types of information including node attribute and node behavior. To further evaluate the effectiveness of each kind of feature, we compare the pure attribute-based method, pure behavior-based method and combination of both them based on wide range evaluation metrics, ROC, AUC, PR and AUPR. The results are as in the following table 2 and figure 7.

Based on a single type of feature, the model can achieve considerable prediction performance under 5-fold cross validation. The representation vectors combine the above two kinds of information, making it easier to construct the classifier and achieve more competitive performance.

In view of the “new sample” (cold start) problem in practical biological experiments, we do not guarantee that the degree of each node is greater than 0. When only the sequences of the biological entities are known and their associations with other

biomolecules are undiscovered, this strategy of constructing the vector by combining the node attribute and the node behavior can also predict potential relationships based on new sample and greatly improve the expansion of the model.

Table 2. Results of accuracy (Acc.), sensitivity (Sen.), specificity (Spec.), precision (Prec.) and MCC obtained by feature importance comparison experiment under 5-fold cross validation on the whole network.

Feature	Acc. (%)	Sen. (%)	Spec. (%)	Prec. (%)	MCC (%)	AUC (%)
Attribute	90.85±0.09	89.79±0.19	91.9±0.11	91.73±0.1	81.72±0.17	95.91±0.05
Behavior	88.67±0.15	82.15±0.24	95.19±0.18	94.47±0.19	78±0.29	93.28±0.13
**Both**	**91.5±0.16**	**87.32±0.31**	**95.68±0.11**	**95.28±0.12**	**83.29±0.29**	**96.08±0.31**

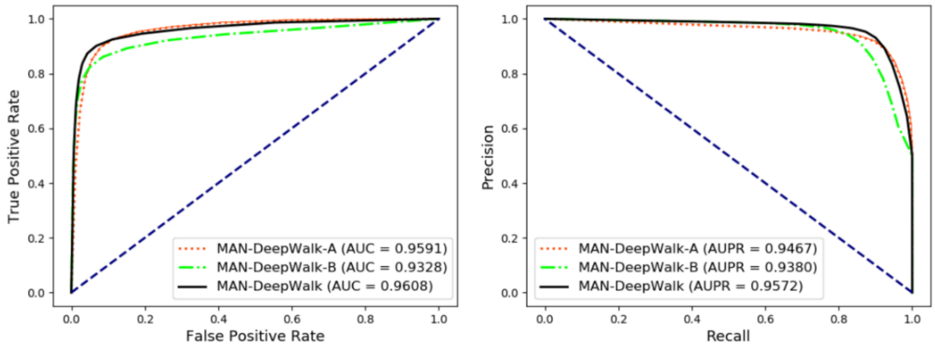

Figure 7. The ROCs, AUCs, PRs, and AUPRs of the proposed method under 5-fold cross validation on the whole dataset.

3.3 Comparison based on varying proportions of training sets

In global relationship prediction, data integrity is considered sensitive and critical. To explore the impact of different ratios of missing data on the results, we separately learn the representation vectors of each node based on varying proportions of edges in the whole graph.

Specifically, 20%, 40%, 60%, and 80% of the edges in the whole network are processed respectively to convert the nodes into vectors by their behavior feature. Meanwhile, the corresponding edges mentioned above are used as the training set to construct the model. The test set is the remaining edges that is 80%, 60%, 40%, and 20% of the whole edges in the graph, respectively. Each node is represented as a vector by only its behavior feature.

Even in the extreme case, *i.e.* 20% of the entire network is used for feature construction and model training. The remaining 80% of all edges are used for testing and evaluation. The proposed model still achieved AUC of 0.8710 and AUPR of 0.8747 which implied that the proposed method with outstanding data mining ability will greatly improve the efficiency of existing biological experiments. The details results can be seen in the Table 3 and Figure 8.

Table 3. Results of accuracy (Acc.), sensitivity (Sen.), specificity (Spec.), precision (Prec.) and MCC obtained trained and tested by different proportions of edges in the entire

network.

Ratio	Acc. (%)	Sen. (%)	Spec. (%)	Prec. (%)	MCC (%)	AUC (%)
20%	82.09	71.99	92.2	90.22	65.54	87.1
40%	85.54	77.48	93.61	92.38	72.03	90.19
60%	87.35	80.2	94.49	93.58	75.47	91.84
80%	88.64	82.35	94.92	94.19	77.89	93.17

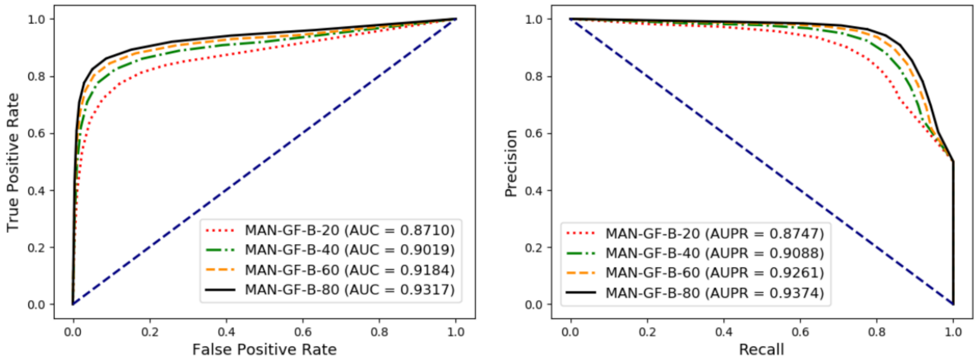

Figure 8. The ROCs, AUCs, PRs, and AUPRs of the proposed method trained and tested by different proportions of edges in the whole network.

3.4 Additional experiment based on drug-disease association prediction

Apart from relationship prediction tasks based on the whole network, we take specific object as research subject and further implement additional experiments on drug-disease association prediction to compare the proposed global model with traditional local method. There are 17,414 experimentally verified drug-disease associations that have been collected from CTD. 5-fold cross validation was performed, and the ROCs and AUCs are shown in the Figure 9.

For Figure 9 (a), it can be treated as the baseline that each node is represented as a 64-dimension vector by only its pure attributes *i.e.* Morgan fingerprints or disease semantics.

For Figure 9 (b), the node behaviors are represented based on only drug-disease associations. It can be regarded as a traditional idea inspired by the "guilt-by-association" that each node is abstracted into a 128-dimension vector by combining attributes and local behaviors. Compared to Figure 9 (a), a slightly elevated AUC confirms the results of feature importance comparison experiment, and shows that the method of measuring the local function of biomolecules improves the prediction performance to some extent.

For Figure 9 (c), it can be considered as a kind of global embedding method that proposed in this paper. In each cross validation, 80% drug-disease pairs along with other all 17 kinds of associations are dealt with Bioentity2vec. Taking the 128-dimension vectors that integrate attribute and behavior as input, Random Forest classifier is chosen for training and testing. The remarkable results compared with traditional local method indicate that the extra edges serve as an intermediary to facilitate the prediction of associations when faced with specific problems.

For Figure 9 (d), we carry out a special embedding strategy inspired by Chen *et al.* [64]. The remaining 17 kinds of relationships without drug-disease association pairs are learned

by DeepWalk to obtain the behavior representation vectors. Therefore, this process does not depend on any direct drug-disease associations. In order to eliminate the influence of the attribute feature on the prediction performance, each node representation vector was constructed by only behavior feature under the special strategy. Nevertheless, the model still achieved an average AUC of 0.7562 under 5-fold cross validation which implies that MAN does contain a wealth of biological information.

Note that in order to ensure the fairness of the experiment, negative samples of 4 experiments and each subset under 5-fold cross validation are all consistent.

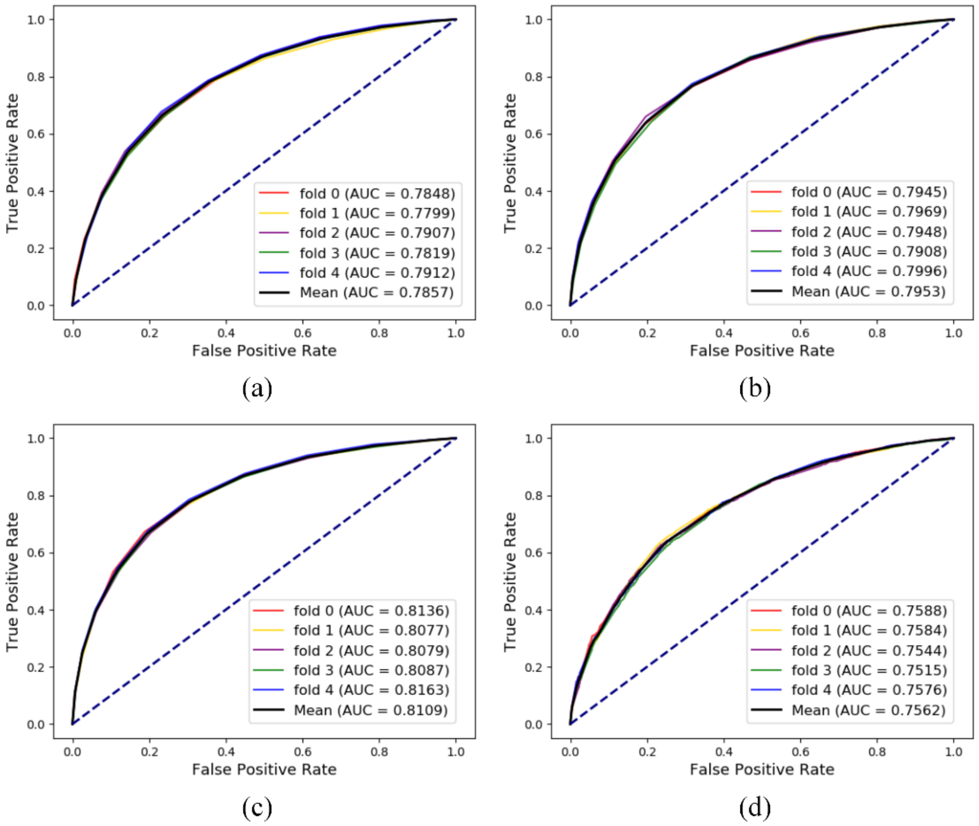

Figure 9. The ROCs, AUCs, PRs, and AUPRs of 4 additional experiments under 5-fold cross validation on dug-disease association dataset.

3.5 A case study based on drug-disease association

A case study of Ataxia was implemented to assess the performance of the proposed method in a real-world environment. As mentioned above, we have collected 17414 drug-disease associations from CTD database [56] and processed them as described in the article of Zhang *et al.* [65]. In order to verify the predicted effect of the proposed model on new disease, we removed 61 association pairs related to Ataxia, the remaining 17353 drug-disease associations were utilized as the training set to generate feature and construct the model. Ataxia is paired with each drug to form the test set. The results of top-10 can be seen in Table 4. All association pairs are verified by the CTD database. Inference Score and References are provided by CTD. The term unconfirmed is an association pair that we cannot find in the CTD. We sorted all drugs by Direct Evidence, and the results of top-10

in Direct Evidence Rank were added to Table 4.

Table 4. The proposed method was applied to Ataxia to predict the potential disease-related drugs, and 8 of top-10 predicted drugs have been confirmed according to CTD database.

Num	DrugBank ID	Evid-ence	CTD Chemical Name	Inference Score	References	Direct Evidence Rank
1	db00313	CTD	Valproic acid	32.61	22	263
2	db00252	CTD	Phenytoin	3.04	32	50
3	db00635	CTD	Prednisone	null	1	178
4	db00563	CTD	Methotrexate	6.89	8	8
5	db00544	CTD	Fluorouracil	3.12	5	46
6	db00907	CTD	Cocaine	4.94	7	18
7	db00477	CTD	Chlorpromazine	3.79	2	31
8	db01577	CTD	Metamfetamine	unconfirmed	unconfirmed	unconfirmed
9	db00661	CTD	Verapamil	null	2	205
10	db00363	CTD	unconfirmed	unconfirmed	unconfirmed	unconfirmed

The unsatisfactory prediction results can be attributed to the following points. In cross-validation, each bioentity can be represented by its attribute and behavior. In an open environment, new sample (cold start) problems are common. The new samples can only be represented by the attributes because there are not adequate known relationships. CTD and DrugBank are 2 different databases, so it is difficult to use sufficient relationships to characterize bioentities. How to unify the identifiers of biological entities and how to integrate as many databases as possible will be the focus of the next step.

Conclusion

Current bioentity relationship computational methods can efficiently predict a single-type relationship, but cannot simultaneously detect complex multi-type relationships among various bioentities. In this article, we abandoned the reductionism idea of modeling on only a single range of transcripts or translations, but collected as many kinds of biomolecules as possible to organize a comprehensive MAN. We also proposed a representation method called Bioentity2vec for generating feature vectors of different types of bioentities. After above operations, each node (Bioentity) in the network (cell) can be characterized as a 128-dimension vector and the random forest classifier is applied to perform relationship prediction task. All results showed that the proposed method achieved remarkable performance in both single-type and multi-type relationship prediction. In general, our studies represent a preliminary exploration from single to complex molecular association network. We believe that this work can bring a technological inspiration and be the basis for further discovery in a large practical need. It can be expected there will appear more variants in both proteomics and genomics in the future, from developing new theoretical methods to broader research objects.

Author Contributions: Z-H.G. and Z-H. Y. considered the algorithm, arranged the datasets, and performed the analyses. D-S. H., H-C. Y., Y-B. W. and Z-H. C. wrote the manuscript. All authors read and approved the final manuscript.

Funding: This work was supported by the grant of National Key R&D Program of China (2018YFA0902600）, and the grants of the National Science Foundation of China, Nos. 61722212, 61861146002, 61732012 & 61902342.

Conflicts of Interest: The authors declare no conflict of interest.

References

1. Barabasi A-L, Oltvai ZN: **Network biology: understanding the cell's functional organization**. *Nature reviews genetics* 2004, **5**(2):101.
2. Hertzberg RP, Pope AJ: **High-throughput screening: new technology for the 21st century**. *Current opinion in chemical biology* 2000, **4**(4):445-451.
3. Moore PB: **The three-dimensional structure of the ribosome and its components.** *Annual review of biophysics and biomolecular structure* 1998, **27**(1):35-58.
4. Mata J, Marguerat S, Bähler J: **Post-transcriptional control of gene expression: a genome-wide perspective**. *Trends in biochemical sciences* 2005, **30**(9):506-514.
5. Singh R: **RNA–protein interactions that regulate pre-mRNA splicing.** *Gene Expression, The Journal of Liver Research* 2002, **10**(1-2):79-92.
6. Tian B, Bevilacqua PC, Diegelman-Parente A, Mathews MB: **The double-stranded-RNA-binding motif: interference and much more.** *Nature reviews Molecular cell biology* 2004, **5**(12):1013.
7. You Z-H, Huang Z-A, Zhu Z, Yan G-Y, Li Z-W, Wen Z, Chen X: **PBMDA: A novel and effective path-based computational model for miRNA-disease association prediction.** *PLoS computational biology* 2017, **13**(3):e1005455.
8. Guo Z-H, You Z-H, Wang Y-B, Yi H-C, Chen Z-H: **A Learning-Based Method for LncRNA-Disease Association Identification Combing Similarity Information and**

Rotation Forest. *iScience* 2019, **19**:786-795.

9. Wang L, You Z-H, Chen X, Li Y-M, Dong Y-N, Li L-P, Zheng K: **LMTRDA: Using logistic model tree to predict MiRNA-disease associations by fusing multi-source information of sequences and similarities**. *PLoS computational biology* 2019, **15**(3):e1006865.

10. Li J-Q, You Z-H, Li X, Ming Z, Chen X: **PSPEL: in silico prediction of self-interacting proteins from amino acids sequences using ensemble learning**. *IEEE/ACM Transactions on Computational Biology and Bioinformatics (TCBB)* 2017, **14**(5):1165-1172.

11. Wang L, You Z-H, Chen X, Yan X, Liu G, Zhang W: **Rfdt: A rotation forest-based predictor for predicting drug-target interactions using drug structure and protein sequence information**. *Current Protein and Peptide Science* 2018, **19**(5):445-454.

12. Li J-Q, Rong Z-H, Chen X, Yan G-Y, You Z-H: **MCMDA: Matrix completion for MiRNA-disease association prediction**. *Oncotarget* 2017, **8**(13):21187.

13. Wang Y-B, You Z-H, Li X, Jiang T-H, Chen X, Zhou X, Wang L: **Predicting protein–protein interactions from protein sequences by a stacked sparse autoencoder deep neural network**. *Molecular BioSystems* 2017, **13**(7):1336-1344.

14. Huang Z-A, Huang Y-A, You Z-H, Zhu Z, Sun Y: **Novel link prediction for large-scale miRNA-lncRNA interaction network in a bipartite graph**. *BMC medical genomics* 2018, **11**(6):113.

15. Ashburn TT, Thor KB: **Drug repositioning: identifying and developing new uses for existing drugs**. *Nature reviews Drug discovery* 2004, **3**(8):673.

16. Chen X, Liu M-X, Cui Q-H, Yan G-Y: **Prediction of disease-related interactions between**

microRNAs and environmental factors based on a semi-supervised classifier. *PloS one* 2012, **7**(8):e43425.

17. Cui H, Zhang M, Yang Q, Li X, Liebman M, Yu Y, Xie L: The Prediction of Drug-Disease Correlation Based on Gene Expression Data. *BioMed research international* 2018, **2018**.

18. Fang S, Zhang L, Guo J, Niu Y, Wu Y, Li H, Zhao L, Li X, Teng X, Sun X: NONCODEV5: a comprehensive annotation database for long non-coding RNAs. *Nucleic acids research* 2017, **46**(D1):D308-D314.

19. Kozomara A, Birgaoanu M, Griffiths-Jones S: miRBase: from microRNA sequences to function. *Nucleic acids research* 2018, **47**(D1):D155-D162.

20. Guo Z-H, Yi H-C, You Z-H: Construction and Comprehensive Analysis of a Molecular Association Network via lncRNA–miRNA–Disease–Drug–Protein Graph. *Cells* 2019, **8**(8):866.

21. Wang Y, You Z-H, Yang S, Li X, Jiang T-H, Zhou X: A High Efficient Biological Language Model for Predicting Protein–Protein Interactions. *Cells* 2019, **8**(2):122.

22. Wang D, Wang J, Lu M, Song F, Cui Q: Inferring the human microRNA functional similarity and functional network based on microRNA-associated diseases. *Bioinformatics* 2010, **26**(13):1644-1650.

23. Ngo DL, Yamamoto N, Tran VA, Nguyen NG, Phan D, Lumbanraja FR, Kubo M, Satou K: Application of word embedding to drug repositioning. *Journal of Biomedical Science and Engineering* 2016, **9**(01):7.

24. Goyal P, Ferrara E: Graph embedding techniques, applications, and performance: A

survey. *Knowledge-Based Systems* 2018, **151**:78-94.

25. Ozcan A, Oguducu SG: **Link prediction in evolving heterogeneous networks using the NARX neural networks**. *Knowledge and Information Systems* 2018, **55**(2):333-360.

26. Ozcan A, Oguducu SG: **Multivariate Time Series Link Prediction for Evolving Heterogeneous Network**. *International Journal of Information Technology & Decision Making (IJITDM)* 2019, **18**(01):241-286.

27. Su C, Tong J, Zhu Y, Cui P, Wang F: **Network embedding in biomedical data science**. *Brief Bioinform* 2018:1-16.

28. Martínez V, Navarro C, Cano C, Fajardo W, Blanco A: **DrugNet: Network-based drug–disease prioritization by integrating heterogeneous data**. *Artificial intelligence in medicine* 2015, **63**(1):41-49.

29. Zeng X, Zhu S, Liu X, Zhou Y, Nussinov R, Cheng F: **deepDR: a network-based deep learning approach to in silico drug repositioning**. *Bioinformatics* 2019.

30. Wei X, Zhang Y, Huang Y, Fang Y: **Predicting drug–disease associations by network embedding and biomedical data integration**. *Data Technologies and Applications* 2019, **53**(2):217-229.

31. Lotfi Shahreza M, Ghadiri N, Mousavi SR, Varshosaz J, Green JR: **A review of network-based approaches to drug repositioning**. *Briefings in bioinformatics* 2017, **19**(5):878-892.

32. Hamilton WL, Ying R, Leskovec J: **Representation learning on graphs: Methods and applications**. *arXiv preprint arXiv:170905584* 2017.

33. Wold S, Esbensen K, Geladi P: **Principal component analysis**. *Chemometrics and*

intelligent laboratory systems 1987, **2**(1-3):37-52.

34. Borg I, Groenen P: **Modern multidimensional scaling: Theory and applications**. *Journal of Educational Measurement* 2003, **40**(3):277-280.

35. Tenenbaum JB, De Silva V, Langford JC: **A global geometric framework for nonlinear dimensionality reduction**. *science* 2000, **290**(5500):2319-2323.

36. Roweis ST, Saul LK: **Nonlinear dimensionality reduction by locally linear embedding**. *science* 2000, **290**(5500):2323-2326.

37. Yao D, Zhang L, Zheng M, Sun X, Lu Y, Liu P: **Circ2Disease: a manually curated database of experimentally validated circRNAs in human disease**. *Scientific reports* 2018, **8**(1):11018.

38. Zhao Z, Wang K, Wu F, Wang W, Zhang K, Hu H, Liu Y, Jiang T: **circRNA disease: a manually curated database of experimentally supported circRNA-disease associations**. *Cell death & disease* 2018, **9**(5):475-475.

39. Bao Z, Yang Z, Huang Z, Zhou Y, Cui Q, Dong D: **LncRNADisease 2.0: an updated database of long non-coding RNA-associated diseases**. *Nucleic acids research* 2018, **47**(D1):D1034-D1037.

40. Fan C, Lei X, Fang Z, Jiang Q, Wu F-X: **CircR2Disease: a manually curated database for experimentally supported circular RNAs associated with various diseases**. *Database* 2018, **2018**.

41. Bhattacharya A, Cui Y: **SomamiR 2.0: a database of cancer somatic mutations altering microRNA–ceRNA interactions**. *Nucleic acids research* 2015, **44**(D1):D1005-D1010.

42. Piñero J, Bravo À, Queralt-Rosinach N, Gutiérrez-Sacristán A, Deu-Pons J, Centeno

E, García-García J, Sanz F, Furlong LI: **DisGeNET: a comprehensive platform integrating information on human disease-associated genes and variants**. *Nucleic acids research* 2016:gkw943.

43. Ma W, Zhang L, Zeng P, Huang C, Li J, Geng B, Yang J, Kong W, Zhou X, Cui Q: **An analysis of human microbe–disease associations**. *Briefings in bioinformatics* 2016, **18**(1):85-97.

44. Hewett M, Oliver DE, Rubin DL, Easton KL, Stuart JM, Altman RB, Klein TE: **PharmGKB: the pharmacogenetics knowledge base**. *Nucleic acids research* 2002, **30**(1):163-165.

45. R Rizkallah M, Gamal-Eldin S, Saad R, K Aziz R: **The pharmacomicrobiomics portal: a database for drug-microbiome interactions**. *Current Pharmacogenomics and Personalized Medicine (Formerly Current Pharmacogenomics)* 2012, **10**(3):195-203.

46. Wishart DS, Feunang YD, Guo AC, Lo EJ, Marcu A, Grant JR, Sajed T, Johnson D, Li C, Sayeeda Z: **DrugBank 5.0: a major update to the DrugBank database for 2018**. *Nucleic acids research* 2017, **46**(D1):D1074-D1082.

47. Chen G, Wang Z, Wang D, Qiu C, Liu M, Chen X, Zhang Q, Yan G, Cui Q: **LncRNADisease: a database for long-non-coding RNA-associated diseases**. *Nucleic acids research* 2012, **41**(D1):D983-D986.

48. Miao Y-R, Liu W, Zhang Q, Guo A-Y: **lncRNASNP2: an updated database of functional SNPs and mutations in human and mouse lncRNAs**. *Nucleic acids research* 2017, **46**(D1):D276-D280.

49. Cheng L, Wang P, Tian R, Wang S, Guo Q, Luo M, Zhou W, Liu G, Jiang H, Jiang Q:

LncRNA2Target v2. 0: a comprehensive database for target genes of lncRNAs in human and mouse. *Nucleic acids research* 2018, **47**(D1):D140-D144.

50. Yuan J, Wu W, Xie C, Zhao G, Zhao Y, Chen R: NPInter v2. 0: an updated database of ncRNA interactions. *Nucleic acids research* 2013, **42**(D1):D104-D108.

51. Huang Z, Shi J, Gao Y, Cui C, Zhang S, Li J, Zhou Y, Cui Q: HMDD v3. 0: a database for experimentally supported human microRNA–disease associations. *Nucleic acids research* 2018, **47**(D1):D1013-D1017.

52. Liu X, Wang S, Meng F, Wang J, Zhang Y, Dai E, Yu X, Li X, Jiang W: SM2miR: a database of the experimentally validated small molecules' effects on microRNA expression. *Bioinformatics* 2012, **29**(3):409-411.

53. Chou C-H, Shrestha S, Yang C-D, Chang N-W, Lin Y-L, Liao K-W, Huang W-C, Sun T-H, Tu S-J, Lee W-H: miRTarBase update 2018: a resource for experimentally validated microRNA-target interactions. *Nucleic acids research* 2017, **46**(D1):D296-D302.

54. Tong Z, Cui Q, Wang J, Zhou Y: TransmiR v2. 0: an updated transcription factor-microRNA regulation database. *Nucleic acids research* 2018, **47**(D1):D253-D258.

55. Szklarczyk D, Gable AL, Lyon D, Junge A, Wyder S, Huerta-Cepas J, Simonovic M, Doncheva NT, Morris JH, Bork P: STRING v11: protein–protein association networks with increased coverage, supporting functional discovery in genome-wide experimental datasets. *Nucleic acids research* 2018, **47**(D1):D607-D613.

56. Davis AP, Grondin CJ, Johnson RJ, Sciaky D, McMorran R, Wiegers J, Wiegers TC, Mattingly CJ: The comparative toxicogenomics database: Update 2019. *Nucleic acids*

research 2018, **47**(D1):D948-D954.

57. Coordinators NR: **Database resources of the national center for biotechnology information**. *Nucleic acids research* 2017, **45**(Database issue):D12.

58. Glažar P, Papavasileiou P, Rajewsky N: **circBase: a database for circular RNAs**. *Rna* 2014, **20**(11):1666-1670.

59. Shen J, Zhang J, Luo X, Zhu W, Yu K, Chen K, Li Y, Jiang H: **Predicting protein–protein interactions based only on sequences information**. *Proceedings of the National Academy of Sciences* 2007, **104**(11):4337-4341.

60. Weininger D: **SMILES, a chemical language and information system. 1. Introduction to methodology and encoding rules**. *Journal of chemical information and computer sciences* 1988, **28**(1):31-36.

61. Rogers D, Hahn M: **Extended-connectivity fingerprints**. *Journal of chemical information and modeling* 2010, **50**(5):742-754.

62. Landrum G: **RDKit: open-source cheminformatics software**. In.; 2016.

63. Perozzi B, Al-Rfou R, Skiena S: **Deepwalk: Online learning of social representations**. In: *Proceedings of the 20th ACM SIGKDD international conference on Knowledge discovery and data mining: 2014*: ACM; 2014: 701-710.

64. Chen X: **Predicting lncRNA-disease associations and constructing lncRNA functional similarity network based on the information of miRNA**. *Scientific reports* 2015, **5**:13186.

65. Zhang W, Yue X, Lin W, Wu W, Liu R, Huang F, Liu F: **Predicting drug-disease associations by using similarity constrained matrix factorization**. *BMC bioinformatics* 2018, **19**(1):233.

Click here to access/download

Supplementary Material

Response_to_revievers_GIGA-D-19-00385R1.docx

Dear Editor,

We would like to submit the manuscript entitled " Bioentity2vec: Attribute- and Behavior-driven Representation for Multi-type Relationship Prediction between Various Bioentities", which we wish to be considered for publication in *gigascience*. It is well-known that *gigascience* pays much attention to interdisciplinary research such as computational biology, bioinformatics and Data-Driven Multicellular Systems Biology Series.

We are grateful to the editor and reviewers for putting in efforts to review the paper with the aim of improving the quality of our paper. We have addressed the concerns of the editor and reviewers in the revised manuscript.

With all best regards,

Zhu-Hong You

Professor

Xinjiang Technical Institute of Physics and Chemistry, Chinese Academy of Science

Address:No.40-1,Beijingnan Road, Urumqi,XinJiang,830011,P.R.China

Email：zhuhongyou@ms.xjb.ac.cn
