# Supplementary material for: Bioentity2vec: Attribute- and behavior-driven representation for predicting multi-type relationships between bioentities
Source: Gigascience. 2020 Jun 13;9(6):giaa032. doi: 10.1093/gigascience/giaa032 (PMC7293023; doi:10.1093/gigascience/giaa032)
Supplement: giaa032_GIGA-D-19-00385_Revision_3 [file giaa032_giga-d-19-00385_revision_3.pdf]

GigaScience

Bioentity2vec: Attribute- and Behavior-driven Representation for Multi-type Relationship Prediction between Various Bioentities

--Manuscript Draft--

**Manuscript Number:**	GIGA-D-19-00385R3
**Full Title:**	Bioentity2vec: Attribute- and Behavior-driven Representation for Multi-type Relationship Prediction between Various Bioentities
**Article Type:**	Research

Funding Information:

Key Technologies Research and Development Program (2018YFA0902600)	Dr. De-Shuang Huang
NSFC Excellent Young Scholars Program (61722212)	Dr. zhuhong you
International Cooperation and Exchange Programme (61861146002)	Dr. De-Shuang Huang
Key Programme (61732012)	Dr. De-Shuang Huang
National Natural Science Foundation of China (61902342)	Mr. Yan-Bin Wang

Abstract:

Abstract

Background : The explosive growth of genomic, chemical and pathological data provides new opportunities and challenges for humans to understand thoroughly life activities in cells. However, there exist few computational models that aggregate various bioentities to comprehensively reveal the physical and functional landscape of biological system.

Results : We construct a Molecular Association Network (MAN) which contains 18 kinds of edges (relationships) among 8 kinds of nodes (bioentities). Based on this, we propose a new bioentities representation method, named Bioentity2vec, which integrates the attribute and behavior information of bioentity. Applying random forest classifier, we achieved promising performance on 18 kinds of relationships, with AUC of 0.9608 and AUPR of 0.9572.

Conclusions : Our study show that constructing a network with rich topological and biological information is of substantially significance for understanding the biological landscape systematically at the molecular level. The competitive results show that our bioentity2vec can effectively represent biological entities and provide easy distinguished information for classification tasks. Moreover, our method can predict the relationship between single type and multiple types at the same time, which will accelerate the process of biological experimental research and industrial product development.

**Corresponding Author:**	zhuhong you<br><br>CHINA
**Corresponding Author Secondary Information:**	
**Corresponding Author's Institution:**	
**Corresponding Author's Secondary Institution:**	
**First Author:**	Zhen-Hao Guo
**First Author Secondary Information:**	
**Order of Authors:**	Zhen-Hao Guo

Powered by Editorial Manager® and ProduXion Manager® from Aries Systems Corporation

	zhuhong you
	Yan-Bin Wang
	De-Shuang Huang
	Hai-Cheng Yi
	Zhan-Heng Chen
**Order of Authors Secondary Information:**	
**Response to Reviewers:**	Authors' Response to Reviewers' Comments<br>Paper title:Bioentity2vec: Attribute- and Behavior-driven Representation for Multi-type Relationship Prediction between Various Bioentities<br>Manuscript ID:GIGA-D-19-00385R1<br>Authors:Zhen-Hao Guo; Zhu-Hong You; Yan-Bin Wang; De-Shuang Huang; Hai-Cheng Yi; Zhan-Heng Chen<br><br>We are grateful to the editor and reviewers for putting in efforts to review the paper with the aim of improving the quality of our paper. We have addressed the concerns of the editor and reviewers in the revised manuscript. In particular, the following revisions have been made.<br><br>Reviewer 1<br>I thank authors for addressing my comments. Few additional points:<br>Response: Thank to the reviewer for this positive comment.<br>Comment 1:<br>1. Fig 2, entities are still referred as biomarker. Additionally, what the dashed borders / lines mean should be specified.<br>Response: Thanks for this useful comment.<br>According to the suggestions of the reviewer, we corrected the picture and added a description of the dashed borders / lines.<br><br>Comment 2:<br>2. Answer to my previous question #4 (optimization of parameters, usage of gensim) should be included/discussed in the text.<br>Response: Thanks for this useful comment.<br>According to the comments, the response has been discussed and highlighted in blue in the revised manuscript.<br><br>Comment 3:<br>3. To demonstrate the practical utility of the predictions, Table 4 should include the name and ranking of the drug as per the known drugs in CTD in addition to the inference score and number of references (as commented in #12, the http://ctdbase.org/detail.go?type=disease&acc=MESH%3aD001259&view=chem). It would also be beneficial, what is the accuracy of their method on predicting the top 10,20,50,100 drugs in CTD (as well as a relevant discussion on these results).<br>Response: Thanks for this useful comment.<br>We have converted the IDs of the drugs in DrugBank to the CTD chemical names and added them to Table 4. We sorted all drugs by Direct Evidence, and the results of top-10 were added to Table 4.<br>Considering that many top drugs in CTD do not appear in the network we construct, the prediction of these drugs actually uses only the attributes of the drug, that is, the molecular structure of the drug. Therefore, it is difficult to reflect the advantages of our proposed method. We add these discussions in the reviewed manuscript and highlight them in blue.<br><br>Again, we appreciate all of your insightful comments. We hope that we have addressed your concerns satisfactorily. Thank you for taking you time and energy to help us improve the paper.<br><br>Reviewer 2<br>The methods are appropriate to the aims of the study, they are well described, and necessary controls are included. The conclusions are adequately supported by the data shown. The quality of language in the manuscript is clear.<br>Response: Thank you very much for helpful comments.

Powered by Editorial Manager® and ProduXion Manager® from Aries Systems Corporation

	Comment 1:<br>The statistical tests used in manuscript is not appropriate. t-test or any other related statistical test is used in order to compare the proposed model with baseline.<br>Response: Thanks for this useful suggestion.<br>As the reviewer said, t-test is an evaluation method widely used in bioinformatics. T-test or any other related statistical test is the more acceptable result. However, the proposed method is a deep learning-based representation algorithm and machine learning-based prediction model. Therefore, we prefer to use computer science evaluation methods. Our follow-up work will complement and improve it.<br><br>Again, we appreciate all of your insightful comments. We hope that we have addressed your concerns satisfactorily. Thank you for taking you time and energy to help us improve the paper.
**Additional Information:**	
**Question**	**Response**
Are you submitting this manuscript to a special series or article collection?	No
**Experimental design and statistics**<br><br>Full details of the experimental design and statistical methods used should be given in the Methods section, as detailed in our Minimum Standards Reporting Checklist. Information essential to interpreting the data presented should be made available in the figure legends.<br><br>Have you included all the information requested in your manuscript?	Yes
**Resources**<br><br>A description of all resources used, including antibodies, cell lines, animals and software tools, with enough information to allow them to be uniquely identified, should be included in the Methods section. Authors are strongly encouraged to cite Research Resource Identifiers (RRIDs) for antibodies, model organisms and tools, where possible.<br><br>Have you included the information requested as detailed in our Minimum Standards Reporting Checklist?	Yes
**Availability of data and materials**	Yes

All datasets and code on which the conclusions of the paper rely must be either included in your submission or deposited in publicly available repositories (where available and ethically appropriate), referencing such data using a unique identifier in the references and in the “Availability of Data and Materials” section of your manuscript.<br><br>Have you have met the above requirement as detailed in our Minimum Standards Reporting Checklist?	

Powered by Editorial Manager® and ProduXion Manager® from Aries Systems Corporation

Manuscript

Click here to access/download;Manuscript;Revised_manuscript_GIGA-D-

Bioentity2vec: Attribute- and behavior-driven representation for predicting multi-type relationships between bioentities

Zhen-Hao Guo[1,2,#], Zhu-Hong You[1,2,#,*], Yan-Bin Wang[3,*], De-Shuang Huang[4], Hai-Cheng Yi[1,2] and Zhan-Heng Chen [1,2]

[1] Xinjiang Technical Institute of Physics and Chemistry, Chinese Academy of Sciences, Urumqi 830011, China; [2] University of Chinese Academy of Sciences, Beijing 100049, China; [3] School of Cyber Science and Technology, Zhejiang University, Hangzhou 310000, Zhejiang, China; [4] Computer Science Department, Tongji University, Shanghai 200000, China

[#] Equal contribution

[*] Correspondence address: Zhu-Hong You. [[1] Xinjiang Technical Institute of Physics and Chemistry, Chinese Academy of Sciences, Urumqi 830011, China; [2] University of Chinese Academy of Sciences, Beijing 100049, China]; Tel. +86-991-367-2967; E-mail: zhuhongyou@ms.xjb.ac.cn.

Yan-Bin Wang. School of Cyber Science and Technology, Zhejiang University, Hangzhou 310000, Zhejiang, China; Tel. [+86-991-367-2967]; E-mail: wangyanbin15@mails.ucas.ac.cn

ORCIDs:

Zhen-Hao Guo: 0000-0002-1965-6988

Zhu-Hong You: 0000-0003-1266-2696

De-Shuang Huang: 0000-0002-6759-2691

Hai-Cheng Yi: 0000-0001-8339-396X

Yan-Bing Wang: 0000-0003-1682-5712

Zhan-Heng Chen: 0000-0002-2331-4446

Abstract

The explosive growth of genomic, chemical and pathological data provides new opportunities and challenges for humans to thoroughly understand life activities in cells. However, there exist few computational models that aggregate various bioentities to comprehensively reveal the physical and functional landscape of biological systems.

We constructed a molecular association network (MAN), which contains 18 edges (relationships) between 8 nodes (bioentities). Based on this, we propose Bioentity2vec, a new method for representing bioentities, which integrates information about the attributes and behaviors of a bioentity. Applying the random forest classifier, we achieved promising performance on 18 relationships, with an area under the curve of 0.9608 and an area under the precision-recall curve of 0.9572.

Our study shows that constructing a network with rich topological and biological information is important for systematic understanding of the biological landscape at the molecular level. Our results show that Bioentity2vec can effectively represent biological entities and provides easily distinguishable information about classification tasks. Our method is also able to simultaneously predict

relationships between single types and multiple types, which will accelerate progress in biological experimental research and industrial product development.

Keywords: Network biology; System biology; Bioentity2vec; Multi-type relationship prediction.

Introduction

In the post-genomic era, a key task is to systematically and comprehensively understand the relationships between bioentities in living cells [1]. The foundation for this mission is the rapid development of high-throughput technologies and the discovery of new transcripts or translations [2]. For example, there is increasing evidence to suggest that biomolecular networks, such as protein–protein interactions, associations between noncoding RNA (ncRNA) and disease, and interactions between drugs and their targets play significant roles in protein synthesis [3], gene expression [4], RNA processing [5] and developmental regulation [6]. Consequently, research into the relationships between bioentities will not only provide novel insights into life processes, but will also facilitate disease prevention, diagnosis, treatment and drug development.

Wet lab experiments to identify relationships between bioentities in large-scale datasets are labor-intensive and time-consuming, and have limited real-world utility. Meanwhile, the extensive amount of accumulated experimental data causes information overload,

which makes it prohibitively costly to acquire valuable knowledge. Hence, biological experiments can be effectively guided by data-based computer modeling methods to accelerate genomics and proteomics research progress [7].

The computational biology community has developed many computational methods, such as matrix factorization [8], machine learning [9] and network analysis [10] to detect previously unknown relationships between entities. Guo *et al.* proposed a computational model to predict potential associations between diseases and long noncoding RNA (lncRNA) by integrating evidence of known associations with disease semantic similarity [11]. Wang *et al.* adopted the logistic model tree methodology to integrate information from multiple sources to discover unknown associations between diseases and microRNA (miRNA) [12]. Li *et al.* used the position-specific scoring matrix (PSSM) to represent proteins, and then put these into an ensemble classifier to predict self-interacting and non-self-interacting proteins [13]. Wang *et al.* utilized rotation forest to discover unknown drug–target interactions by drug structure and protein sequence [14].

However, the incompleteness of the data constrains the credibility of predictions made by these methods, resulting in high false positive and false negative rates (FPR and FNR, respectively) [15]. In recent years, the discovery of new types of bioentities and their relationships has provided novel insights to improve this situation to some extent. Additional bioentities may be considered as bridges to synergistically facilitate our knowledge of underlying biological principles and improve prediction. For example, Chen *et al.* were able to effectively improve the prediction of miRNA–disease

associations by taking environmental factors into account [16]. Similarly, Cui *et al.* drew from gene expression data to make preliminary explorations into predicting drug–disease associations [17].

In the past few years, much molecular data has accumulated, but computational methods have failed to make significant breakthroughs, since few people regard cells as being complete units. In fact, cells comprise nodes (bioentities) and edges (relationships), much like a network (graph), to maintain normal life activities and physiological functions. Being able to establish connections between internal or external factors and gene expression would be helpful for understanding biological systems. Here, we constructed a molecular association network, based on various online databases, such as NONCODE [18] and miRbase [19], to help systematically analyze the relationships between bioentities within human cells.

Faced with such a large-scale network, the most critical challenge is how to quickly and effectively describe the nodes. In general, each bioentity can be defined by its own attributes and behaviors [20, 21]. Attribute features can be represented by RNA sequences, drug chemical structures, etc. [22–24]. The semantic description of drug or disease can also be considered as a kind of representation, which is widely used in relationship prediction tasks, such as drug reposition [24]. On the other hand, network-based methods, especially the rapid development of graph-embedding (network representation) algorithms, has provided great hope for being able to clearly describe relationships between nodes [25–32].

Graph embedding, in which nodes are represented in a network as dense vector forms,

is chosen to respond to this situation [33]. Although some existing bioinformatics models contain the idea of graph embedding, many still focus on traditional techniques, including principal component analysis (PCA) [34], multidimensional scaling (MDS) [35], Isomap [36], and local linear embeddings (LLE) [37]. In general, these methods offer satisfactory performance for small networks. However, at least quadratic time complexity restricts the application of these methods to large-scale data. Recently, deep learning has attracted research attention. Here, the representation method DeepWalk is applied.

We constructed a molecular association network (MAN) and propose a graph-embedding algorithm to represent each node as a vector (Figure 1). Specifically, 18 kinds of associations or interactions between eight kinds of biomolecules were collected from various databases to construct the network. The lower triangular part of the adjacency matrix, A, simplifies calculation and storage. Each bioentity can be represented as a vector by combining attribute and behavior features (see flowchart in Figure 2). We used random forest to predict multi-type relationships, across an entire network, obtaining an area under the receiver operating characteristic curve (AUC) of 0.9608, and an area under the precision-recall curve (AUPR) of 0.9572, using five-fold cross-validation. Furthermore, we implemented three experiments to compare feature importance, embedding strategy and proportions of training sets. Our results suggest the potential utility of MAN for revealing previously uncovered relationships. We hope that this work can provide assistance and guidance for wet experiments, and be useful for researchers seeking to understand gene regulation and disease mechanisms, and to

discover new drugs at the molecular level.

Materials and methods

2.1 Construction of the molecular association network (MAN)

To construct the MAN, 18 different experimentally verified associations or interactions were collected from various databases [38–57]. After unifying identifiers, we obtained eight types of bioentity. All relationships and bioentities were then aggregated to form the MAN. The quantity and proportion of each type of bioentity or relationships is shown in Figure 3.

2.2 Node attribute representation: k-mer, semantics and fingerprint

Protein and RNA sequences, disease and microbe semantics, and drug chemical structure are all essential features. We represented these as vectors using the following methods.

For protein, mRNA, miRNA, lncRNA and circular RNA (circRNA), sequences were collected from STRING (STRING, RRID:SCR_005223) [56], NCBI (NCBI, RRID:SCR_006472) [58], miRBase (miRbase, RRID:SCR_003152) [19], NONCODE (NONCODE, RRID:SCR_007822) [18], and circBase [59], respectively. Proteins are composed of 20 different amino acids; using the method described by Shen *et al.* [60], we first classified these into four categories based on amino acid side chain polarity: 1.

alanine (Ala), valine (Val), leucine (Leu), isoleucine (Ile), methionine (Met), phenylalanine (Phe), tryptophan (Trp), and proline (Pro); 2. Glycine (Gly), serine (Ser), threonine (Thr), cysteine (Cys), asparagine (Asn), glycine (Gln), and tyrosine (Tyr); 3. arginine (Arg), lysine (Lys), histidine (His); and 4. aspartate (Asp), and glutamic acid (Glu). RNA, including mRNA, miRNA, lncRNA and circRNA, is composed of four nucleotides: adenine (A), guanine (G), cytosine (C) and uracil (U), with the same sequence composition, so we directly encode their original sequences without pre-treatment. Each RNA molecule or protein can be represented as a vector by *k*-mer, in which all dimensions represent the full permutation of *k* nucleotide (or amino acid) combinations, and the value of each dimension is the normalized frequency of the corresponding *k*-mer appearing in the sequence. In this paper, $k = 3$, and each protein or RNA can be represented as a 64-dimension ($4^3 = 4 \times 4 \times 4$) vector.

Diseases and microbes were characterized using Medical Subject Headings (MeSH) descriptors. Top-level categories in the MeSH tree structure are: anatomy [A], organisms [B], diseases [C], and so on. The categories corresponding to microbes and diseases are B and C, respectively. As done by Wang *et al.* [23], we construct a directed acyclic graph (DAG) of diseases and microbes (see Figure 4) to represent them through their semantics. For example, a microbe *M* can be represented as a graph $DAG(M) = (M,N(M),E(M))$ where $N(M)$ is the set of all nodes in *M*'s DAG, and $E(M)$ is the set of all edges in *M*'s DAG. The semantic contribution of microbe *m*, which is in the node set $N(M)$ to *M*, can be defined as:

$$\begin{cases} V_M(m) = 1 & if\ m = M \\ V_M(m) = max\left\{\Delta * V\left(m^{'}\right) | m^{'} \in children\ of\ m\right\} & if\ m \neq M \end{cases} \quad (1)$$

where Δ denotes an attenuation factor and is defined as 0.5, according to previous literature [23]. In the DAG generated by microbe *M*, *M*'s contribution to itself can be regarded as the maximum and equals to 1; the remaining diseases will contribute less and less to *M* as the distance increases. Therefore, the sum of the contributions of microbes, which are in the set *N(M)* to *M*, can be calculated as follows:

$$SV(M) = \Sigma_{m \in N(M)} V_M(m) \quad (2)$$

The similarity between microbes *i* and *j* can then be calculated as follows:

$$Similarity(i,j) = \frac{\Sigma_{m \in N(i) \cap N(j)} (V_i(m) + V_j(m))}{SV(i) + SV(j)} \quad (3)$$

The node attribute of microbe or disease can be represented by semantics similarity, which is converted into a 64-dimensional vector after feature extraction and transformation using the stack autoencoder. A DAG example of the microbe *Staphylococcus* is as follows: for drugs, we download their Simplified Molecular Input Line Entry Specification (SMILES) [61] from DrugBank (DrugBank, RRID:SCR_002700) [47]. Then, SMILES is transformed into corresponding Morgan molecular fingerprints [62] using the Python package RDKit (RDKit, RRID:SCR_014274) [63]. To unify dimensions and improve feature quality, stack autoencoder is used to convert each original molecular fingerprint into a 64-dimensional vector.

2.3 Node behavior representation: DeepWalk

Using 'guilt-by-association' assumptions, we use a more general behavioral feature in complex networks. Generally speaking, this involves embedding representations of known edges between nodes in a network. Despite this, a row or column of the adjacency matrix can directly be utilized as a representation vector for node behavior in a one-hot encoding method. However, there is no concept of similarity between each dimension of such high-dimensional, sparse vectors, because it is represented as indices in a relationship. Meanwhile, the one-hot encoding method takes up a lot of storage space and is not conducive to the input of downstream tasks. Hence, how to extract information about behavior from nodes in complex networks such as a MAN, is challenging.

Here, we use a network embedding method called DeepWalk [64]. The main idea is to obtain a certain length of the walk sequence through Random Walk, an ideal mathematical state of Brownian motion that can repeatedly access the visited nodes. After obtaining enough sequences, the vectors of the nodes can be learned by the SkipGram model. The direct analog is to estimate the likelihood of observing vertex v_i, given all the previous vertices visited so far in the random walk, *i.e.*

$$P_r\big(v_i|(v_1, v_2, \dots, v_{i-1})\big) \tag{4}$$

The goal is to learn a latent representation and the mapping function is:

$$\Phi: v \in V \mapsto R^{|V|\times d} \tag{5}$$

The problem then, is to estimate the likelihood:

$$P_r\big(v_i|(\Phi(v_1), \Phi(v_2), \dots, \Phi(v_{i-1}))\big) \tag{6}$$

The recent relaxation in language modeling turns the prediction problem, and this yields the optimization problem:

$$\underset{\Phi}{minimize} = -logP_r(\{v_{i-w}, \ldots, v_{i+w}\} \backslash v_i | \Phi(v_i)) \quad (7)$$

The main steps of the algorithm are as follows:

Algorithm 1: DeepWalk ($\boldsymbol{G, w, d, \gamma, t}$).

Input: graph $\boldsymbol{G(V, E)}$

window size $\boldsymbol{w}$

embedding size $\boldsymbol{d}$

walks per vertex $\boldsymbol{\gamma}$

walk length $\boldsymbol{t}$

Output: matrix of vertex representations $\boldsymbol{\Phi} \in \mathrm{R}^{|\mathrm{V}| \times \mathrm{d}}$

1: Initialization: sample $\boldsymbol{\Phi}$ **from** $\boldsymbol{U}^{|V| \times d}$

2: Build a binary tree T from V

3: for $\boldsymbol{i}$ **=0 to** $\boldsymbol{\gamma}$ **do**

4: $\boldsymbol{O}$ **= Shuffle (**$\boldsymbol{V}$**)**

5: for each $\boldsymbol{v_i} \in \boldsymbol{O}$ **do**

6: $\boldsymbol{W_{v_i}}$ **= RandomWalk** $(\mathbf{G}, \mathbf{v_i}, \mathbf{t})$

7: SkipGram $(\boldsymbol{\Phi}, \boldsymbol{W_{v_i}}, \boldsymbol{w})$

8: end for

9: end for

The effects of parameters *w* and *t* on the results were not obvious. At the same time, smaller values can significantly reduce the experimental running time. Larger values of

w and *t* may introduce additional noise and increase calculation burden. In fact, the structure of the MAN is totally different from those of previous benchmark datasets such as Facebook and Twitter. For traditional social networks, vertices with the same label are closely related. In the network of this manuscript, there are generally no edges between vertices of the same label, except in a protein–protein interaction network. The representation of vertices is mainly through the description of relationship with other types of vertices. To ensure as much experimental reproducibility as possible, we set the parameters *w* and *t* to the commonly used values 10 and 80. After generating the sequence of vertices, a Python package called *gensim* was applied to generate word-embedding representation.

The SkipGram algorithm is as follows:

Algorithm 2: SkipGram $(\boldsymbol{\Phi}, \boldsymbol{W_{v_i}}, \boldsymbol{w})$

for each $\boldsymbol{v_j} \in \boldsymbol{W_{v_i}}$ **do**

for each $\boldsymbol{u_k} \in \boldsymbol{W_{v_i}}[\boldsymbol{j} - \boldsymbol{w} : \boldsymbol{j} + \boldsymbol{w}]$ **do**

$\boldsymbol{J}(\boldsymbol{\Phi}) = -\boldsymbol{logPr}(\boldsymbol{u_k} | \boldsymbol{\Phi}(\boldsymbol{v_j}))$

$\boldsymbol{\Phi} = \boldsymbol{\Phi} - \alpha * \frac{\partial J}{\partial \boldsymbol{\Phi}}$

end for

end for

Note: whenever nodes are processed by DeepWalk, the test edges (relationships) in the network are stripped to ensure that the label information does not leak into the test set. A visualization of DeepWalk can be seen in Figure 5.

2.4 Stack autoencoder (SAE)

Attribute representation vectors of drugs and diseases comprise thousands of dimensions, and this is not helpful for classifier training. Stack autoencoder (SAE) is selected to map the vectors from the original space into low space, so as to reduce noise and feature dimensions. The autoencoder consists of two parts: the encoder, which maps the original input to the new space, and the decoder, which reconstructs the latent representation to the original input. For the original input *x*, the output h_1 of the first hidden layer can be calculated by the following formula:

$$h_1 = f_1(W_1 x + b_1) \tag{8}$$

where f_1 is the activation function, W_1 is the weight matrix between the input layer and the first hidden layer, and b_1 is the threshold of the first hidden layer neurons. Similarly, the output of each layer of the stack autoencoder can be calculated. The mean squared error between the output *y* and the original input *x* is:

$$L = (x, y) = \sum_i (x_i - y_i)^2 \tag{9}$$

Then, the back-propagation algorithm is used to minimize the loss function to obtain the final model. We completed this task using the Python package *Keras*. The dimension of the hidden layer representation is 64, 'MSE' is selected as the loss function and the optimizer is 'Adam'. The epochs and batch sizes are set to 10 and 128, respectively.

2.5 Random forest classifier

Random forest is a classifier containing multiple decision trees whose output is determined by the mode of the output of each decision tree. It can efficiently process high-dimensional features, even in large data volumes. In addition, its high adaptability makes it possible to accept both discrete and continuous data. Here, we used the Python package *sklearn* to perform the random forest classifier, with default values.

Results

3.1. Relationship prediction based on the whole dataset under 5-fold cross validation

Relationship prediction is common in both academia and industry. Here, some edges in the original graph are hidden as test sets and we construct the model based on the residual network. We evaluate the proposed method through five-fold cross validation. Under this strategy, the whole dataset is divided into five mutually exclusive subsets of roughly equal size. Each subset is used as the test set in turn to assess the effect of the classifier, and the remaining four subsets are utilized as a training set to construct the model. In each fold, areas under the receiver operating characteristic curves (ROC) and precision-recall curves (PR) are drawn to visualize the results, respectively. There are 114,150 valid experimental relationships in the whole network. In each fold cross-validation, 80% of the edges of the entire network are processed by Bioentity2vec and are treated as training samples; 20% of edges are considered test samples.

Various evaluation criteria, including accuracy (Acc.), sensitivity (Sen.), specificity

(Spec.), precision (Prec.) and Matthews correlation coefficient (MCC) are adopted to measure experimental results. Results are shown in Table 1 and Figure 6, and show that our method can help to make stable and robust decisions and accurately discover potential associations.

Table 1. Results of accuracy (Acc.), sensitivity (Sen.), specificity (Spec.), precision (Prec.) and Matthews correlation coefficient (MCC) obtained under five-fold cross-validation on the whole network

fold	Acc. (%)	Sen. (%)	Spec. (%)	Prec. (%)	MCC (%)	AUC (%)
0	91.66	87.49	95.83	95.45	83.61	96.49
1	91.66	87.71	95.61	95.23	83.58	96.29
2	91.33	86.9	95.76	95.35	82.99	95.86
3	91.47	87.32	95.62	95.22	83.23	95.73
4	91.37	87.18	95.56	95.16	83.04	96.03
**Average**	**91.5±0.16**	**87.32±0.31**	**95.68±0.11**	**95.28±0.12**	**83.29±0.29**	**96.08±0.31**

3.2. Feature importance comparison

Nodes in a MAN can be represented as vectors by two types of information: node attribute and node behavior. To evaluate the effectiveness of these different kinds of feature, we compared the pure attribute-based method, pure behavior-based method and a combination of these, based on various evaluation metrics: ROC, AUC, PR and

AUPR. Results are shown in Table 2 and Figure 7, and show that the feature vector generated by combining the two kinds of information above provides more competitive performance.

Considering the “new sample” (cold start) problem in practical biological experiments, we do not guarantee that the degree of each node is greater than 0. When only the sequences of the biological entities are known and their associations with other biomolecules are unknown, this strategy of constructing the vector by combining the node attribute and the node behavior can also predict potential relationships based on new sample and greatly improve the usability of the model.

Table 2. Results of accuracy (Acc.), sensitivity (Sen.), specificity (Spec.), precision (Prec.) and matthews correlation coefficient (MCC) obtained by feature importance comparison experiments under five-fold cross-validation on the whole network

Feature	Acc. (%)	Sen. (%)	Spec. (%)	Prec. (%)	MCC (%)	AUC (%)
Attribute	90.85±0.09	89.79±0.19	91.9±0.11	91.73±0.1	81.72±0.17	95.91±0.05
Behavior	88.67±0.15	82.15±0.24	95.19±0.18	94.47±0.19	78±0.29	93.28±0.13
**Both**	**91.5±0.16**	**87.32±0.31**	**95.68±0.11**	**95.28±0.12**	**83.29±0.29**	**96.08±0.31**

3.3 Comparison based on varying proportions of training sets

Data integrity is a top priority in achieving global relationship prediction. To explore the effects of missing data on the results, we separately learned the representation vectors of each node in the whole graph. We built models using varying proportions of

edges and evaluated their performance.

Specifically, the dataset was divided into four parts: 20%, 40%, 60%, and 80% of the edges of the full graph as training samples. Correspondingly, the remaining edges of the graph, 80%, 60%, 40%% and 20%, were used as test samples. Here, each node is characterized only by its behavioral feature.

It can be seen from Table 3 and Figure 8 that, when only 20% of the edges of the entire network are used to generate node features and model construction, our method still achieves an AUC of 0.8710 and an AUPR of 0.8747. This demonstrates the excellent data-mining ability of this method.

Table 3. Results of accuracy (Acc.), sensitivity (Sen.), specificity (Spec.), precision (Prec.) and Matthews correlation coefficient (MCC) obtained trained and tested by different proportions of edges in the entire network

Ratio	Acc. (%)	Sen. (%)	Spec. (%)	Prec. (%)	MCC (%)	AUC (%)
20%	82.09	71.99	92.2	90.22	65.54	87.1
40%	85.54	77.48	93.61	92.38	72.03	90.19
60%	87.35	80.2	94.49	93.58	75.47	91.84
80%	88.64	82.35	94.92	94.19	77.89	93.17

3.4 Additional experiment based on drug–disease association prediction

Here, we take a specific example of drug–disease relationship prediction to carry out

an additional experiment to evaluate the performance of our method, and compare it with the traditional single-function method. In total, 17,414 experimentally verified drug–disease associations were collected from the Comparative Toxicogenomics Database (CTD) [57]. Five-fold cross-validation was performed; ROCs and AUCs are shown in Figure 9.

In Figure 9 (a), the baseline for each node is represented as a 64-dimension vector by only its pure attributes, *i.e.* Morgan fingerprints or disease semantics.

For Figure 9 (b), node behaviors are represented based on only drug–disease associations. Taking the idea of "guilt-by-association", each node is abstracted into a 128-dimension vector by combining attributes and single-type associations. Compared to Figure 9 (a), a slightly elevated AUC confirms the results of our feature importance comparison experiment, and shows that measuring the local function of biomolecules improves prediction performance to some extent.

Figure 9 (c), shows we can consider the method proposed in this paper as a kind of global embedding method. In each cross-validation, Bioentity2vec handles 80% drug–disease pairs with 17 kinds of relationships. Taking the 128-dimension vectors that integrate attributes and behaviors as inputs, random forest classifier is chosen for training and testing. Compared with previous methods, the results we obtained, indicate that the extra edges serve as an intermediary to facilitate the prediction of associations when faced with specific problems.

For Figure 9 (d), we carried out a special embedding strategy based on that described by Chen *et al.* [65]. The remaining 17 types of relationship without drug–disease

association pairs were learned by DeepWalk to obtain behavior representation vectors. This process does not depend on direct drug–disease associations. To eliminate the influence of the attribute feature on prediction performance, each node representation vector was constructed only by using behavior features under this special strategy. Nevertheless, the model still achieved an average AUC of 0.7562 under five-fold cross-validatio,n which implies that our MAN contains a wealth of biological information. Note: to ensure the fairness of the experiment, negative samples of four experiments and each subset under five-fold cross-validation were all consistent.

3.5 A case study based on drug–disease association

A case study of ataxia was implemented to assess the performance of the proposed method in a real-world environment. As mentioned, we collected 17,414 drug–disease associations from CTD [57], and processed these as described by Zhang *et al.* [66]. To verify the prediction ability of the proposed model for new disease, we removed 61 association pairs related to ataxia. The remaining 17,353 drug–disease associations were utilized as a training set to generate features and construct the model. Ataxia is paired with each drug to form the test set. The top 10 results can be seen in Table 4. All association pairs were verified by CTD. Inference score and references were provided by CTD. The term ‘unconfirmed’ is an association pair that we were not able to find in the CTD. We sorted all drugs by Direct Evidence Rank, and the top 10 results are shown in Table 4.

Table 4. The proposed method was applied to ataxia to predict potential disease-related drugs. Eight of the top 10 predicted drugs were confirmed in the CTD database.

Num	DrugBank ID	Evidence	CTD chemical name	Inference score	References	Direct Evidence Rank
1	db00313	CTD	Valproic acid	32.61	22	263
2	db00252	CTD	Phenytoin	3.04	32	50
3	db00635	CTD	Prednisone	null	1	178
4	db00563	CTD	Methotrexate	6.89	8	8
5	db00544	CTD	Fluorouracil	3.12	5	46
6	db00907	CTD	Cocaine	4.94	7	18
7	db00477	CTD	Chlorpromazine	3.79	2	31
8	db01577	CTD	Metamfetamine	unconfirmed	unconfirmed	unconfirmed
9	db00661	CTD	Verapamil	null	2	205
10	db00363	CTD	unconfirm	unconfir	unconfirme	unconfirmed

Such prediction results can be attributed to the following two points: 1. in an open environment, there are many problems associated with new samples (cold start). These samples can only be represented by attributes because there are not enough known relationships. 2. CTD and DrugBank are two different databases, and their differences lead to insufficient relationships to generate expressive behavior representations of abiotic entities.

Conclusion

Current biological entity relationship calculation methods only focus on a single type of relationship, and cannot simultaneously detect complex multi-type relationships between bioentities. The model proposed here may solve this issue. Specifically, in developing a comprehensive molecular association network, we propose the use of Bioentity2vec to generate representation vectors for different bioentities. Combined with the random forest classifier, promising results have been demonstrated in single- and multi-type relationship prediction. Our research represents a preliminary exploration from isolated molecules to complex molecular association networks. The concepts expressed in our research may yield novel ideas for the development of new theoretical systems, expansion of research objects, and accelerate the integration of proteomics and genomics.

Availability of supporting data

All source code and supporting data are available in the *GigaScience* GigaDB database [67] and Github [68].

Availability of supporting source code and requirements

Project name: Bioentity2vec

Project home page: https://github.com/CocoGzh/Bioentity2vec

Operating system(s): Windows

Programming language: Python 3.7

Other requirements: Anaconda3, Open-NE

License: MIT

RRID: SCR_018179

List of abbreviations

Acc: accuracy; AUC: Area under receiver operating characteristic curve; AUPR: Area under precision-recall curve; CTD: Comparative Toxicogenomics Database; DAG: Directed acyclic graph; FNR: False negative rate; FPR: False positive rate; MAN: Molecular association network; MCC: Matthews correlation coefficient; MDS: Multidimensional scaling; MeSH: Medical subject heading; PCA: Principal component analysis; PR: Precision-recall curve; Prec: Precision; PSSM: Position-specific scoring matrix; ROC: Receiver operating characteristic curve; SAE: Stack autoencoder; Sen:

Sensitivity; SMILES: Simplified molecular input line entry specification; Spec: Specificity.

Consent for publication

Not applicable.

Competing interests

The authors declare that they have no competing interests.

Funding

This work was supported by a grant from the National Key R&D Program of China (grant number 2018YFA0902600), and grants from the National Science Foundation of China (grant numbers 61722212, 61861146002, 61732012 and 61902342).

Authors' contributions

Z-H.G. and Z-H.Y. considered the algorithm, arranged the datasets, and performed the analyses. D-S.H., H-C.Y., Y-B.W. and Z-H.C. wrote the manuscript. All authors read and approved the final manuscript.

Acknowledgements

Not applicable.

References

1. Barabasi A-L and Oltvai ZN. Network biology: understanding the cell's functional organization. Nat Rev Genet 2004;52:101.

2. Hertzberg RP and Pope AJ. High-throughput screening: new technology for the 21st century. Curr Opin Chem Biol 2000;4 4:445–51.

3. Moore PB. The three-dimensional structure of the ribosome and its components. Annual review of biophysics and biomolecular structure. 1998;27 1:35-58.

4. Mata J, Marguerat S and Bähler J. Post-transcriptional control of gene expression: a genome-wide perspective. Trends in biochemical sciences. 2005;30 9:506-14.

5. Singh R. RNA–protein interactions that regulate pre-mRNA splicing. Gene Expression, The Journal of Liver Research. 2002;10 1-2:79-92.

6. Tian B, Bevilacqua PC, Diegelman-Parente A and Mathews MB. The double-stranded-RNA-binding motif: interference and much more. Nature reviews Molecular cell biology. 2004;5 12:1013.

7. You Z-H, Huang Z-A, Zhu Z, Yan G-Y, Li Z-W, Wen Z, et al. PBMDA: A novel and effective path-based computational model for miRNA-disease association prediction. PLoS computational biology. 2017;13 3:e1005455.

8. Li J-Q, Rong Z-H, Chen X, Yan G-Y and You Z-H. MCMDA: Matrix completion for MiRNA-disease association prediction. Oncotarget. 2017;8 13:21187.

9. Wang Y-B, You Z-H, Li X, Jiang T-H, Chen X, Zhou X, et al. Predicting protein–protein interactions from protein sequences by a stacked sparse autoencoder deep neural network. Molecular BioSystems. 2017;13 7:1336-44.

10. Huang Z-A, Huang Y-A, You Z-H, Zhu Z and Sun Y. Novel link prediction for large-scale miRNA-lncRNA interaction network in a bipartite graph. BMC medical genomics. 2018;11 6:113.

11. Guo Z-H, You Z-H, Wang Y-B, Yi H-C and Chen Z-H. A Learning-Based Method for LncRNA-Disease Association Identification Combing Similarity Information and Rotation Forest. iScience. 2019;19:786-95. doi:10.1016/j.isci.2019.08.030.

12. Wang L, You Z-H, Chen X, Li Y-M, Dong Y-N, Li L-P, et al. LMTRDA: Using logistic model tree to predict MiRNA-disease associations by fusing multi-source information of sequences and similarities. PLoS computational biology. 2019;15 3:e1006865.

13. Li J-Q, You Z-H, Li X, Ming Z and Chen X. PSPEL: in silico prediction of self-interacting proteins from amino acids sequences using ensemble learning. IEEE/ACM Transactions on Computational Biology and Bioinformatics (TCBB). 2017;14 5:1165-72.

14. Wang L, You Z-H, Chen X, Yan X, Liu G and Zhang W. Rfdt: A rotation forest-based predictor for predicting drug-target interactions using drug structure and protein sequence information. Current Protein and Peptide Science. 2018;19 5:445-54.

15. Ashburn TT and Thor KB. Drug repositioning: identifying and developing new uses for existing drugs. Nature reviews Drug discovery. 2004;3 8:673.

16. Chen X, Liu M-X, Cui Q-H and Yan G-Y. Prediction of disease-related interactions

between microRNAs and environmental factors based on a semi-supervised classifier. PloS one. 2012;7 8:e43425.

17. Cui H, Zhang M, Yang Q, Li X, Liebman M, Yu Y, et al. The Prediction of Drug-Disease Correlation Based on Gene Expression Data. BioMed research international. 2018;2018.

18. Fang S, Zhang L, Guo J, Niu Y, Wu Y, Li H, et al. NONCODEV5: a comprehensive annotation database for long non-coding RNAs. Nucleic acids research. 2017;46 D1:D308-D14.

19. Kozomara A, Birgaoanu M and Griffiths-Jones S. miRBase: from microRNA sequences to function. Nucleic acids research. 2018;47 D1:D155-D62.

20. Guo Z-H, Yi H-C and You Z-H. Construction and Comprehensive Analysis of a Molecular Association Network via lncRNA–miRNA–Disease–Drug–Protein Graph. Cells. 2019;8 8:866.

21. Guo Z-H, You Z-H and Yi H-C. Integrative Construction and Analysis of Molecular Association Network in Human Cells by Fusing Node Attribute and Behavior Information. Molecular Therapy-Nucleic Acids. 2020;19:498-506.

22. Wang Y, You Z-H, Yang S, Li X, Jiang T-H and Zhou X. A High Efficient Biological Language Model for Predicting Protein–Protein Interactions. Cells. 2019;8 2:122.

23. Wang D, Wang J, Lu M, Song F and Cui Q. Inferring the human microRNA functional similarity and functional network based on microRNA-associated diseases. Bioinformatics. 2010;26 13:1644-50.

24. Ngo DL, Yamamoto N, Tran VA, Nguyen NG, Phan D, Lumbanraja FR, et al. Application of word embedding to drug repositioning. Journal of Biomedical Science and Engineering. 2016;9 01:7.

25. Goyal P and Ferrara E. Graph embedding techniques, applications, and performance: A survey. Knowledge-Based Systems. 2018;151:78-94.

26. Ozcan A and Oguducu SG. Link prediction in evolving heterogeneous networks using the NARX neural networks. Knowledge and Information Systems. 2018;55 2:333-60.

27. Ozcan A and Oguducu SG. Multivariate Time Series Link Prediction for Evolving Heterogeneous Network. International Journal of Information Technology & Decision Making (IJITDM). 2019;18 01:241-86.

28. Su C, Tong J, Zhu Y, Cui P and Wang F. Network embedding in biomedical data science. Brief Bioinform. 2018:1-16.

29. Martínez V, Navarro C, Cano C, Fajardo W and Blanco A. DrugNet: Network-based drug–disease prioritization by integrating heterogeneous data. Artificial intelligence in medicine. 2015;63 1:41-9.

30. Zeng X, Zhu S, Liu X, Zhou Y, Nussinov R and Cheng F. deepDR: a network-based deep learning approach to in silico drug repositioning. Bioinformatics. 2019;35 24:5191-8.

31. Wei X, Zhang Y, Huang Y and Fang Y. Predicting drug–disease associations by network embedding and biomedical data integration. Data Technologies and Applications. 2019;53 2:217-29.

32. Lotfi Shahreza M, Ghadiri N, Mousavi SR, Varshosaz J and Green JR. A review of network-based approaches to drug repositioning. Briefings in bioinformatics. 2017;19 5:878-92.

33. Hamilton WL, Ying R and Leskovec J. Representation learning on graphs: Methods and applications. arXiv preprint arXiv:170905584. 2017.

34. Wold S, Esbensen K and Geladi P. Principal component analysis. Chemometrics and intelligent laboratory systems. 1987;2 1-3:37-52.

35. Borg I and Groenen P. Modern multidimensional scaling: Theory and applications. Journal of Educational Measurement. 2003;40 3:277-80.

36. Tenenbaum JB, De Silva V and Langford JC. A global geometric framework for nonlinear dimensionality reduction. science. 2000;290 5500:2319-23.

37. Roweis ST and Saul LK. Nonlinear dimensionality reduction by locally linear embedding. science. 2000;290 5500:2323-6.

38. Yao D, Zhang L, Zheng M, Sun X, Lu Y and Liu P. Circ2Disease: a manually curated database of experimentally validated circRNAs in human disease. Scientific reports. 2018;8 1:11018.

39. Zhao Z, Wang K, Wu F, Wang W, Zhang K, Hu H, et al. circRNA disease: a manually curated database of experimentally supported circRNA-disease associations. Cell death & disease. 2018;9 5:475-.

40. Bao Z, Yang Z, Huang Z, Zhou Y, Cui Q and Dong D. LncRNADisease 2.0: an updated database of long non-coding RNA-associated diseases. Nucleic acids research. 2018;47 D1:D1034-D7.

41. Fan C, Lei X, Fang Z, Jiang Q and Wu F-X. CircR2Disease: a manually curated database for experimentally supported circular RNAs associated with various diseases. Database. 2018; 1-6.

42. Bhattacharya A and Cui Y. SomamiR 2.0: a database of cancer somatic mutations altering microRNA–ceRNA interactions. Nucleic acids research. 2015;44 D1:D1005-D10.

43. Piñero J, Bravo À, Queralt-Rosinach N, Gutiérrez-Sacristán A, Deu-Pons J, Centeno E, et al. DisGeNET: a comprehensive platform integrating information on human disease-associated genes and variants. Nucleic acids research. 2017; 45:D833–D839..

44. Ma W, Zhang L, Zeng P, Huang C, Li J, Geng B, et al. An analysis of human microbe–disease associations. Briefings in bioinformatics. 2016;18 1:85-97.

45. Hewett M, Oliver DE, Rubin DL, Easton KL, Stuart JM, Altman RB, et al. PharmGKB: the pharmacogenetics knowledge base. Nucleic acids research. 2002;30 1:163-5.

46. R Rizkallah M, Gamal-Eldin S, Saad R and K Aziz R. The pharmacomicrobiomics portal: a database for drug-microbiome interactions. Current Pharmacogenomics and Personalized Medicine (Formerly Current Pharmacogenomics). 2012;10 3:195-203.

47. Wishart DS, Feunang YD, Guo AC, Lo EJ, Marcu A, Grant JR, et al. DrugBank 5.0: a major update to the DrugBank database for 2018. Nucleic acids research. 2017;46 D1:D1074-D82.

48. Chen G, Wang Z, Wang D, Qiu C, Liu M, Chen X, et al. LncRNADisease: a database for long-non-coding RNA-associated diseases. Nucleic acids research. 2012;41 D1:D983-D6.

49. Miao Y-R, Liu W, Zhang Q and Guo A-Y. lncRNASNP2: an updated database of functional SNPs and mutations in human and mouse lncRNAs. Nucleic acids research. 2017;46 D1:D276-D80.

50. Cheng L, Wang P, Tian R, Wang S, Guo Q, Luo M, et al. LncRNA2Target v2. 0: a comprehensive database for target genes of lncRNAs in human and mouse. Nucleic acids research. 2018;47 D1:D140-D4.

51. Yuan J, Wu W, Xie C, Zhao G, Zhao Y and Chen R. NPInter v2. 0: an updated database of ncRNA interactions. Nucleic acids research. 2013;42 D1:D104-D8.

52. Huang Z, Shi J, Gao Y, Cui C, Zhang S, Li J, et al. HMDD v3. 0: a database for experimentally supported human microRNA–disease associations. Nucleic acids research. 2018;47 D1:D1013-D7.

53. Liu X, Wang S, Meng F, Wang J, Zhang Y, Dai E, et al. SM2miR: a database of the experimentally validated small molecules’ effects on microRNA expression. Bioinformatics. 2012;29 3:409-11.

54. Chou C-H, Shrestha S, Yang C-D, Chang N-W, Lin Y-L, Liao K-W, et al. miRTarBase update 2018: a resource for experimentally validated microRNA-target interactions. Nucleic acids research. 2017;46 D1:D296-D302.

55. Tong Z, Cui Q, Wang J and Zhou Y. TransmiR v2. 0: an updated transcription factor-microRNA regulation database. Nucleic acids research. 2018;47 D1:D253-D8.

56. Szklarczyk D, Gable AL, Lyon D, Junge A, Wyder S, Huerta-Cepas J, et al. STRING v11: protein–protein association networks with increased coverage, supporting functional discovery in genome-wide experimental datasets. Nucleic acids research. 2018;47 D1:D607-D13.

57. Davis AP, Grondin CJ, Johnson RJ, Sciaky D, McMorran R, Wiegers J, et al. The comparative toxicogenomics database: Update 2019. Nucleic acids research. 2018;47 D1:D948-D54.

58. Coordinators NR. Database resources of the national center for biotechnology information. Nucleic acids research. 2017;45 Database issue:D12.

59. Glažar P, Papavasileiou P and Rajewsky N. circBase: a database for circular RNAs. Rna. 2014;20 11:1666-70.

60. Shen J, Zhang J, Luo X, Zhu W, Yu K, Chen K, et al. Predicting protein–protein interactions based only on sequences information. Proceedings of the National Academy of Sciences. 2007;104 11:4337-41.

61. Weininger D. SMILES, a chemical language and information system. 1. Introduction to methodology and encoding rules. Journal of chemical information and computer sciences. 1988;28 1:31-6.

62. Rogers D and Hahn M. Extended-connectivity fingerprints. Journal of chemical information and modeling. 2010;50 5:742-54.

63. Landrum G. RDKit: open-source cheminformatics software. 2016.

64. Perozzi B, Al-Rfou R and Skiena S. Deepwalk: Online learning of social representations. In: *Proceedings of the 20th ACM SIGKDD international*

conference on Knowledge discovery and data mining 2014, pp.701-10.

65. Chen X. Predicting lncRNA-disease associations and constructing lncRNA functional similarity network based on the information of miRNA. Scientific reports. 2015;5:13186.

66. Zhang W, Yue X, Lin W, Wu W, Liu R, Huang F, et al. Predicting drug-disease associations by using similarity constrained matrix factorization. BMC bioinformatics. 2018;19 1:233.

67. Guo Z; You Z; Huang D; Yi H; Wang Y; Chen Z. Supporting data for "Bioentity2vec: Attribute- and Behavior-driven Representation for Multi-type Relationship Prediction between Various Bioentities" *GigaScience Database*. 2020, http://dx.doi.org/10.5524/100713.

68. Github. 2020. https://github.com/CocoGzh/Bioentity2vec.

Figure legends

Figure 1. An example of visualization based on molecular association networks (MAN), in which different colors represent different types of bioentities. Each bioentity contains two kinds of information: node behavior (relationships with other nodes) and node attribute (sequences of protein or RNA, chemical structure of drug, and semantics of disease and microbe).

Figure 2. Flowchart of the proposed method. Each node in the network can be described in two ways: 1. by attribute feature, such as sequence and chemical structure, which can be learned as a 64-dimension vector by *k*-mer etc., and 2. By behavior feature, which

can be represented as a 64-dimension vector through DeepWalk. Attribute and behavior feature are distinguished by dashed and unprocessed squares. After combining attribute and behavior information, each node can be represented as a 128-dimension vector. Positive samples are experimentally verified relationships, while negative samples are the same number of unlabeled relationships that are randomly selected in matrix *A*. Taking the low-dimensional dense vectors as input, random forest is used for prediction.

Figure 3. Details about the quantity and distribution of eight kinds of biomolecules and 18 kinds of relationships.

Figure 4. Construction of the directed acyclic graph (DAG) of *Staphylococcus*. The father node of the current microbe can be obtained by deleting the last three digits of the descriptor. For example, for *Bacillales* (B03.353.500, B03.510.100), we can remove the last three digits to get *Firmicutes* (B03.353) and Gram-positive bacteria (B03.510).

Figure 5. A visualization of DeepWalk. Vertex sequences can be obtained by random walks in the graph. Then, sequences are regarded as sentences, and vertexes as words. The SkipGram algorithm is used to obtain the embedding representation of the vertexes.

Figure 6. Performance obtained by the proposed method. Based on the whole network, the model achieved an area under the receiver operating characteristic curve (AUC) of 0.9608 and an area under the precision-recall curve (AUPR) of 0.9572 under five-fold

cross-validation.

Figure 7. The receiver operating characteristic curves (ROCs), areas under the receiver operating characteristic curves (AUCs), precision-recall curves (PRs), and areas under the precision-recall curves (AUPRs) of the proposed method under five-fold cross-validation. Representations of vectors combining attribute and behavior features are better than single types of information.

Figure 8. Performance comparison achieved by the proposed method, which was trained on different proportions of edges in the molecular association network.

Figure 9. Comparison of receiver operating characteristic curves (ROCs), areas under the receiver operating characteristic curves (AUCs), precision-recall curves (PRs), and areas under the precision-recall curves (AUPRs) with four kinds of representation methods under five-fold cross-validation based on a drug–disease association dataset.

Click here to access/download;Figure;Figure 1.tif

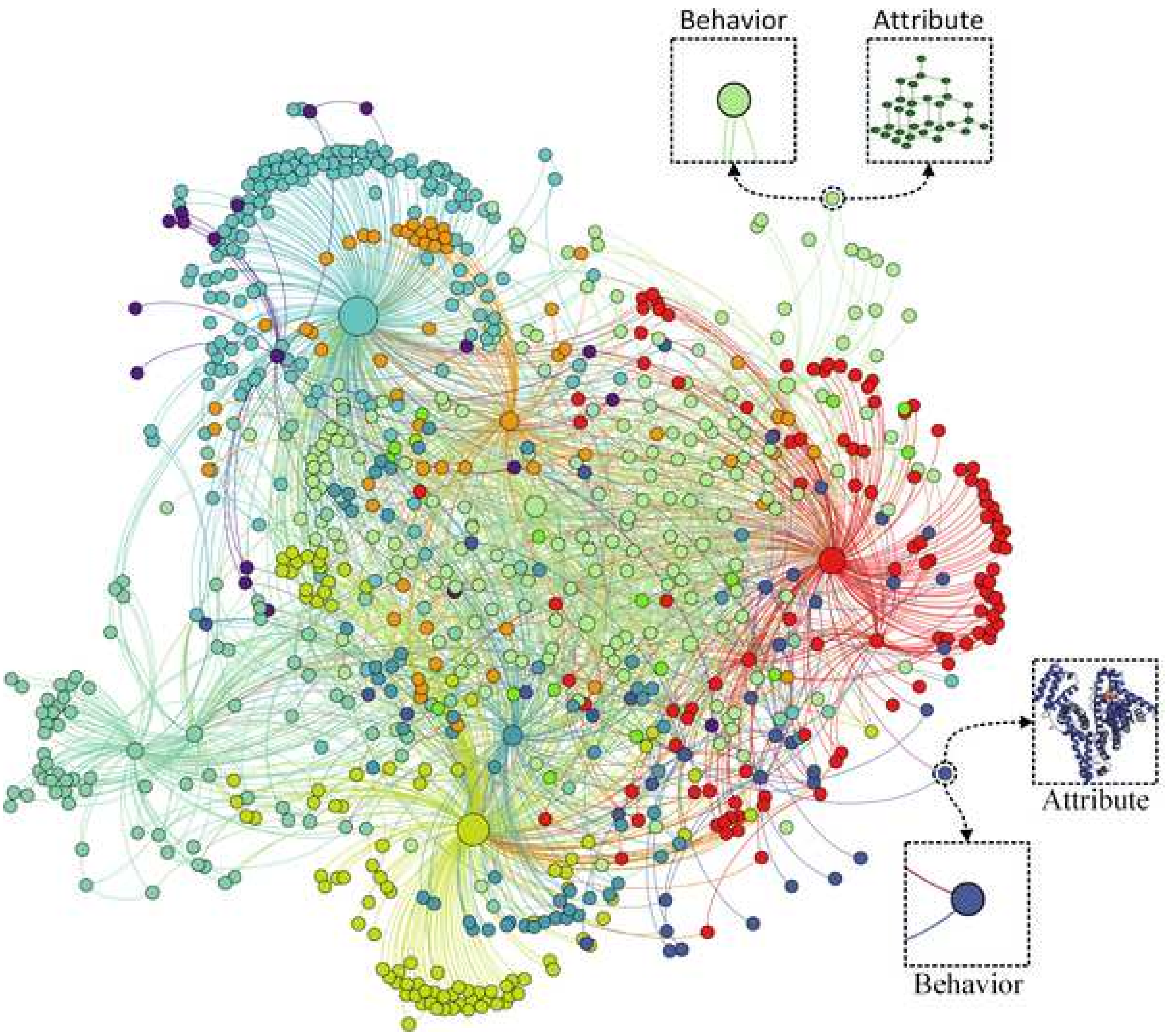

Figure2

Click here to access/download;Figure;Figure 2.tif

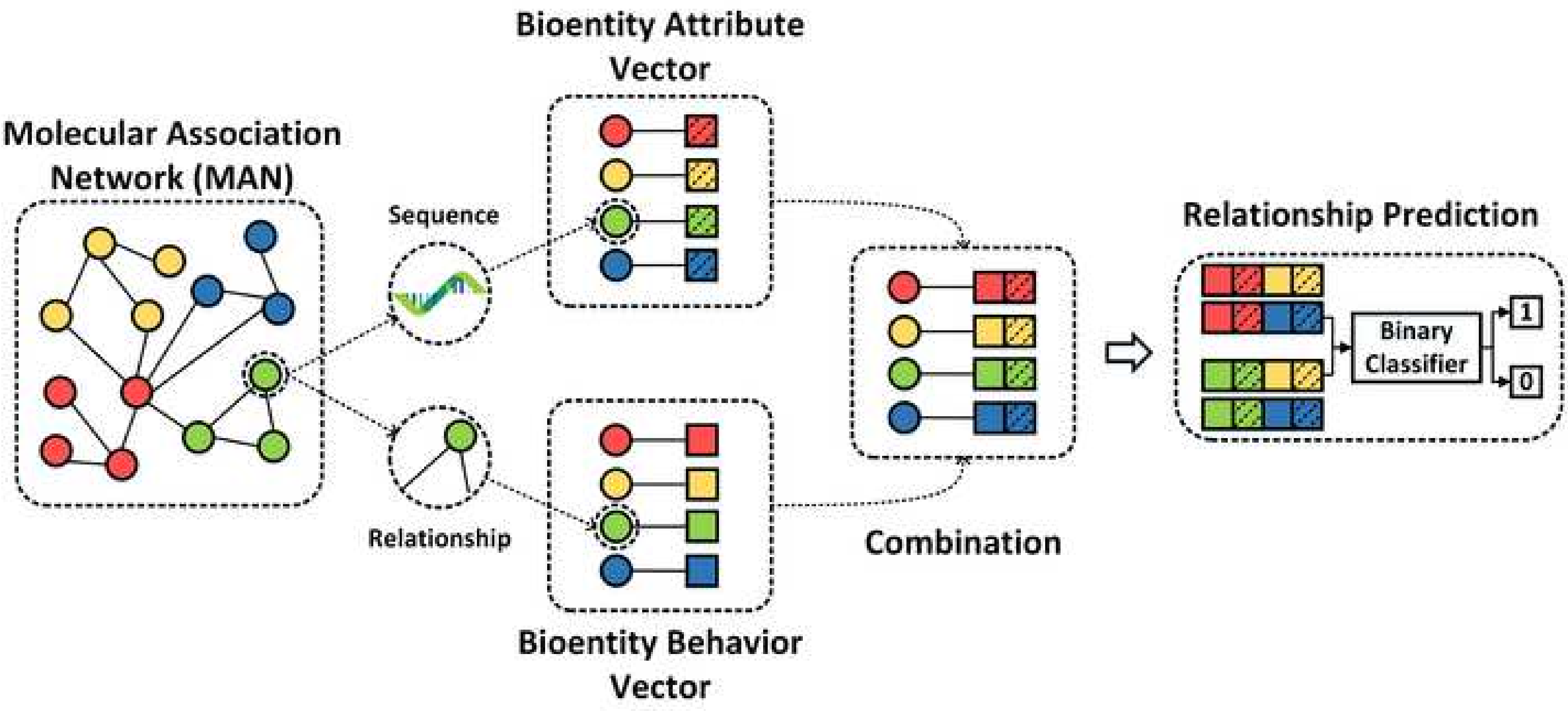

Figure3

Click here to access/download;Figure;Figure 3.tif

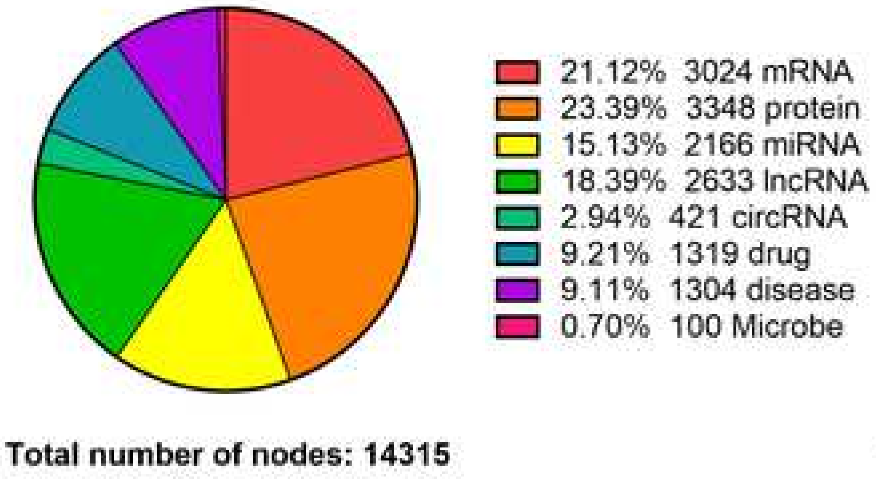

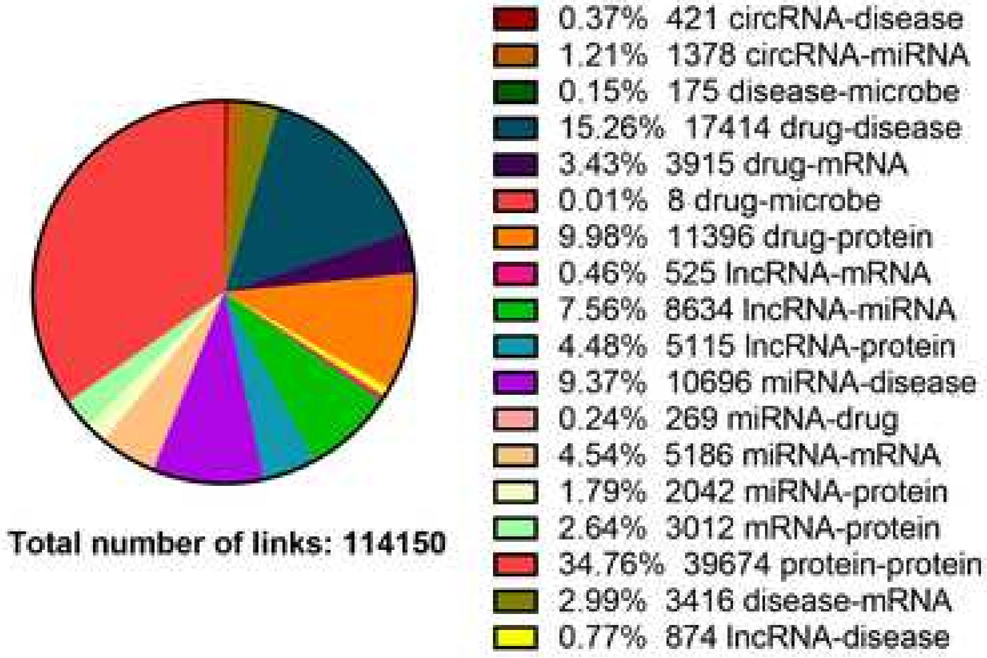

Figure4

Click here to access/download;Figure;Figure 4.tif

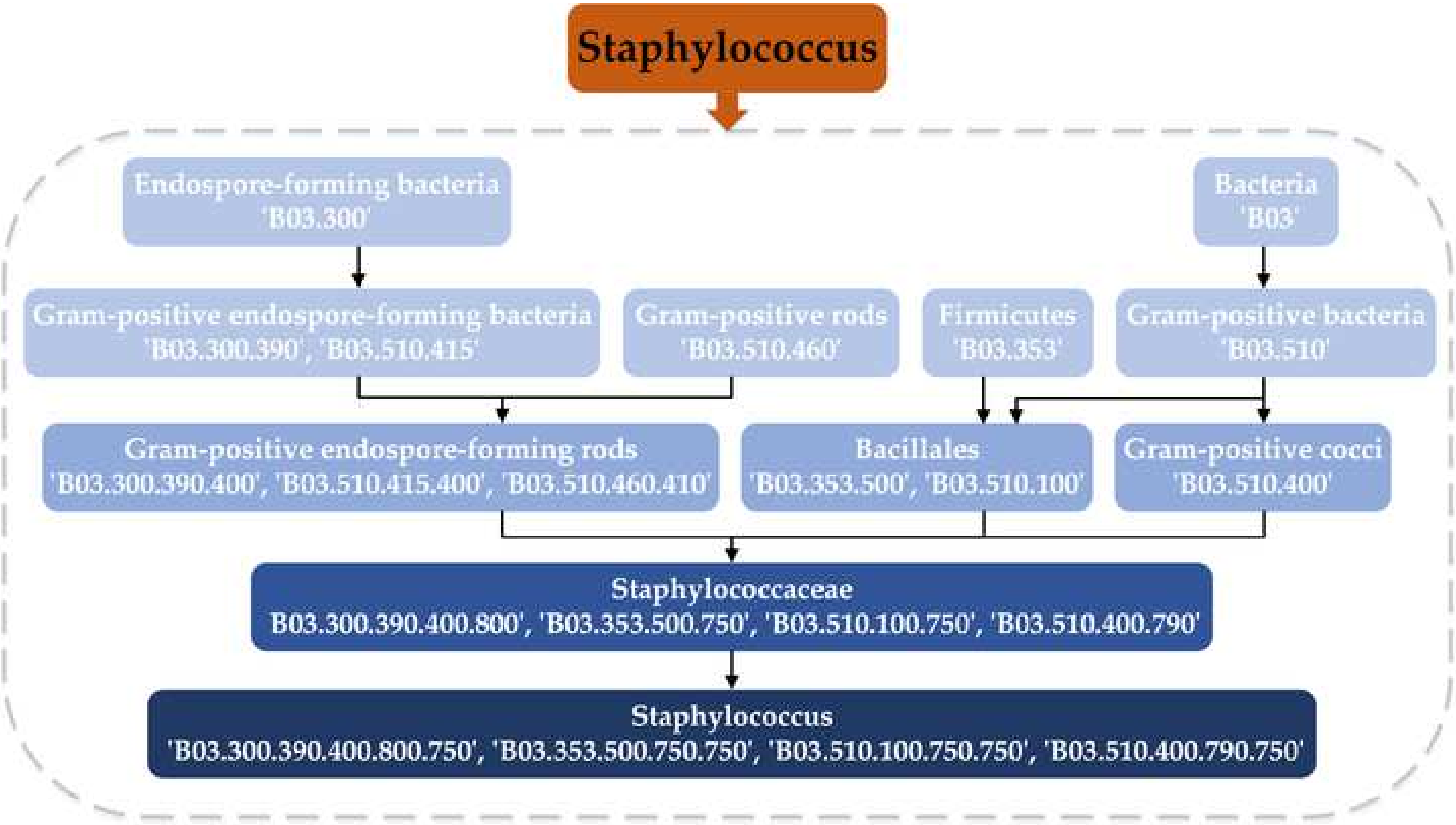

Figure5

Click here to access/download;Figure;Figure 5.tif

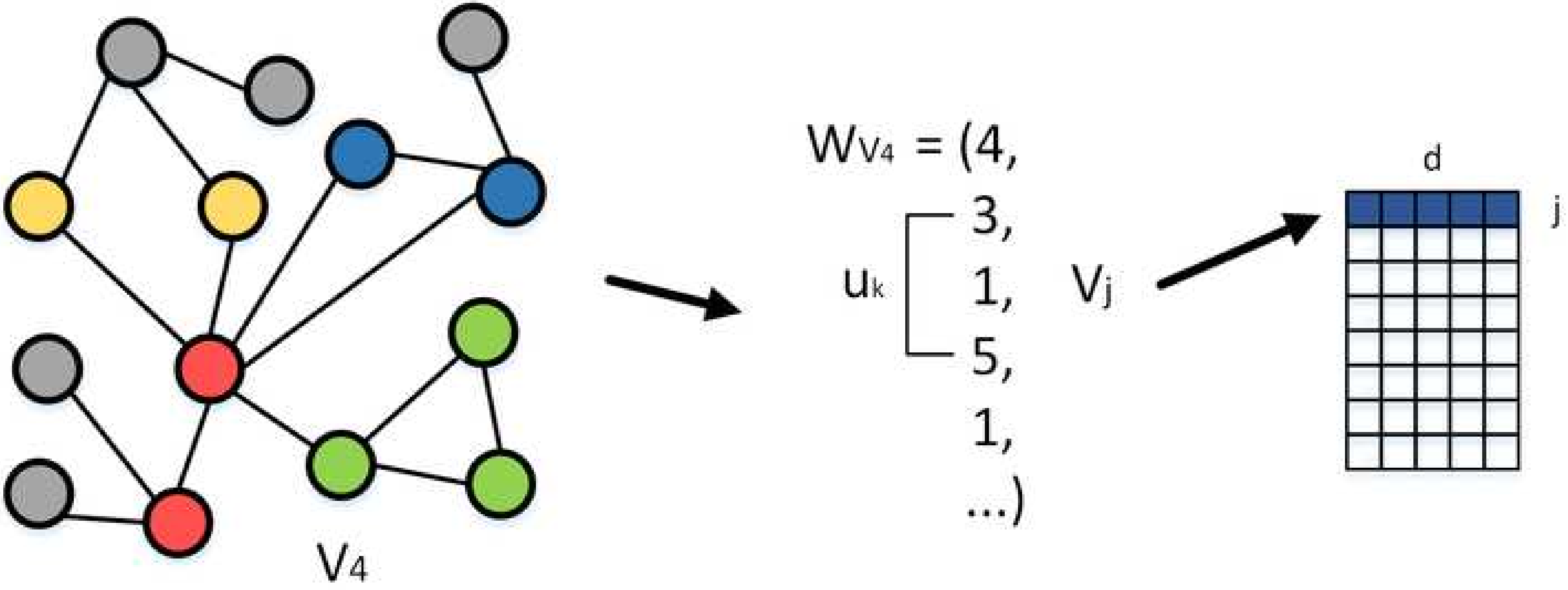

Figure6

Click here to access/download;Figure;Figure 6.tif

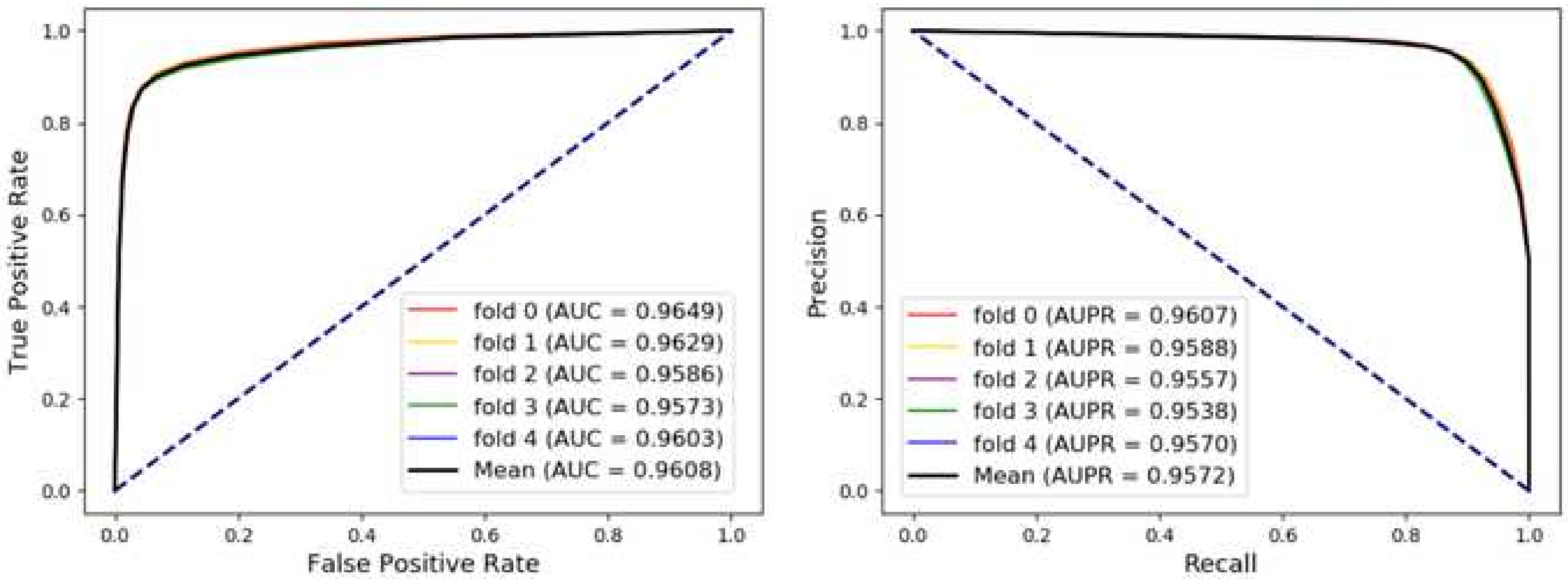

Figure7

Click here to access/download;Figure;Figure 7.tif

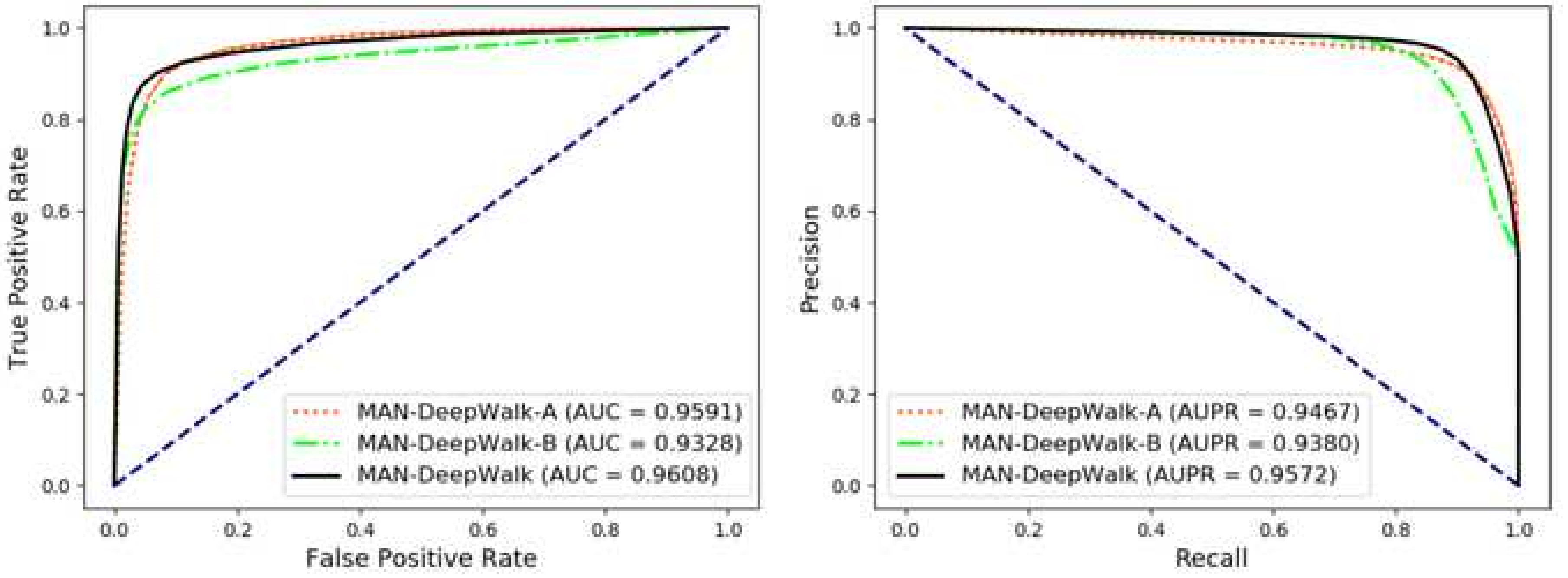

Figure8

Click here to access/download;Figure;Figure 8.tif

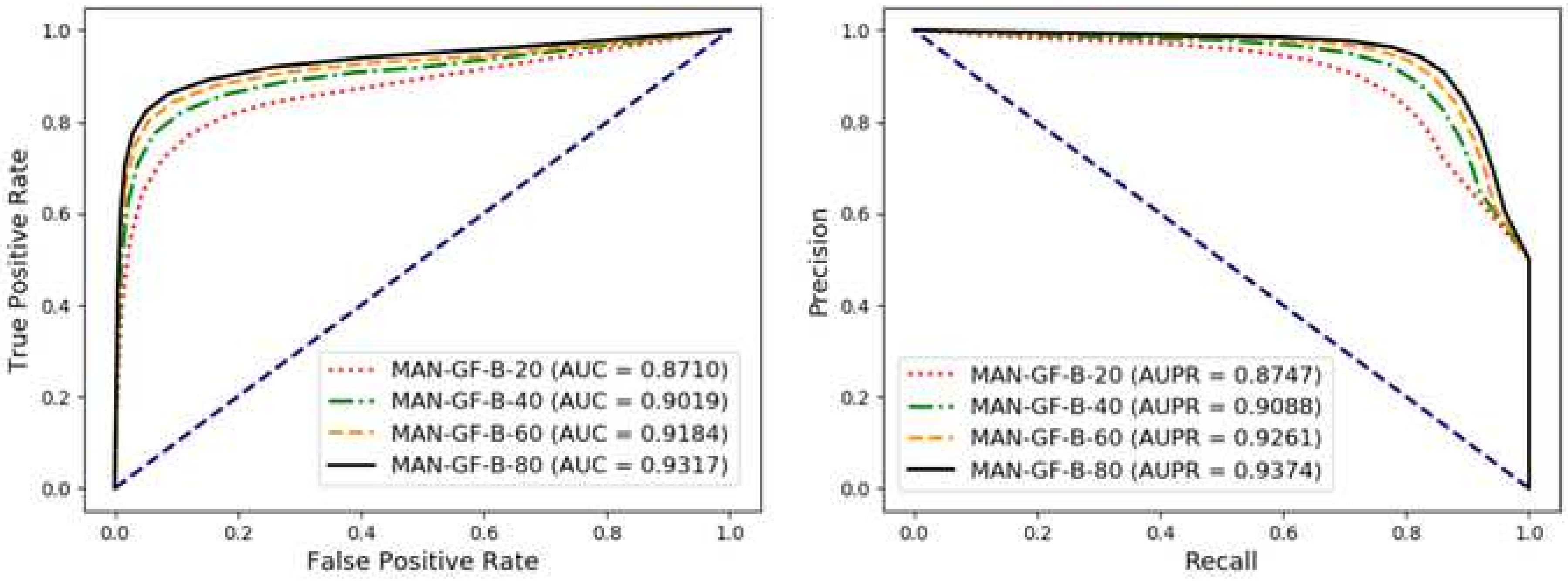

Figure9 Click here to access/download;Figure;Figure 9.tif

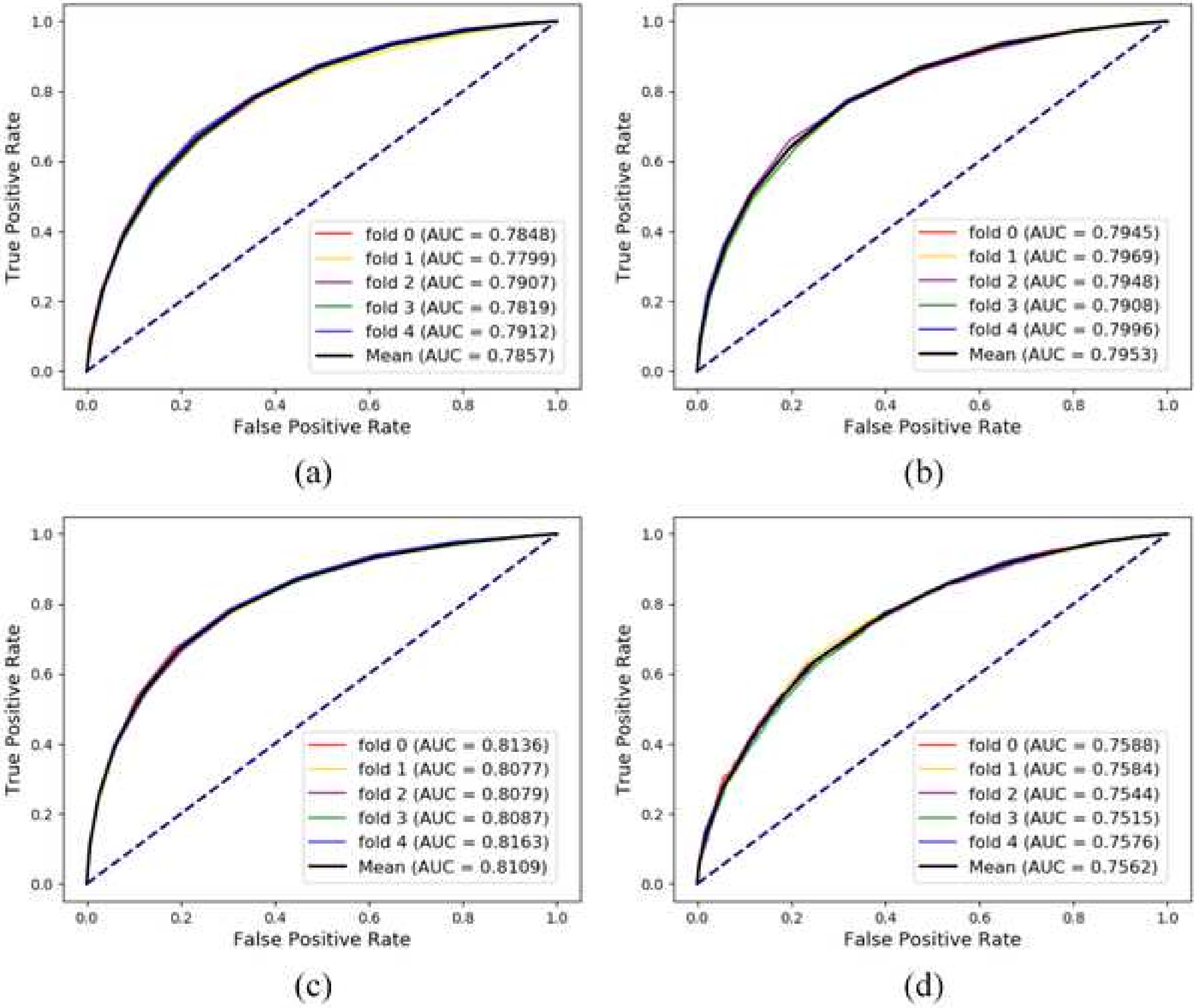

Click here to access/download
Supplementary Material
Response_to_revievers_GIGA-D-19-00385R1.docx

Personal Cover Click here to access/download;Personal Cover;cover letter.doc

Dear Editor,

We would like to submit the manuscript entitled " Bioentity2vec: Attribute- and Behavior-driven Representation for Multi-type Relationship Prediction between Various Bioentities", which we wish to be considered for publication in *gigascience*. It is well-known that *gigascience* pays much attention to interdisciplinary research such as computational biology, bioinformatics and Data-Driven Multicellular Systems Biology Series.

We are grateful to the editor and reviewers for putting in efforts to review the paper with the aim of improving the quality of our paper. We have addressed the concerns of the editor and reviewers in the revised manuscript.

The authors declare no conflict of interest.

All authors have approved the submission of the manuscript.

We confirm that the content of the manuscript has not been published, or submitted for publication elsewhere

With all best regards,

Zhu-Hong You

Professor

Xinjiang Technical Institute of Physics and Chemistry, Chinese Academy of Science

Address: No.40-1,Beijingnan Road, Urumqi,XinJiang,830011,P.R.China

Email：zhuhongyou@ms.xjb.ac.cn
