# Supplementary material for: Bioentity2vec: Attribute- and behavior-driven representation for predicting multi-type relationships between bioentities
Source: Gigascience. 2020 Jun 13;9(6):giaa032. doi: 10.1093/gigascience/giaa032 (PMC7293023; doi:10.1093/gigascience/giaa032)
Supplement: giaa032_Response_to_Reviewer_Comments_Original_Submission [file giaa032_response_to_reviewer_comments_original_submission.pdf]

Author's Response To Reviewer Comments

Close

Authors’ Response to Reviewers’ Comments
Paper title: Biomarker2vec: Attribute- and Behavior-driven Representation for Multi-type Relationship Prediction between Various Biomarkers
Manuscript ID: GIGA-D-19-00385
Authors: Zhen-Hao Guo; Zhu-Hong You; De-Shuang Huang; Hai-Cheng Yi; Yan-Bin Wang; Zhan-Heng Chen

We are grateful to the editor and reviewers for putting in efforts to review the paper with the aim of improving the quality of our paper. We have addressed the concerns of the editor and reviewers in the revised manuscript. In particular, the following revisions have been made.

Reviewer 1
Guo an colleagues present biomarker2vec, an approach to represent biomolecule as features in low dimensional representation space and provide an application of these features in the prediction of drug-disease relationships using a random forest based classifier. A molecular association network (MAN) is generated using data from various biological databases in which nodes and edges are represented as 64 dimensional vectors. Nodes are described based on the occurrences of 3-mers using a 4-letter alphabet for proteins and nucleotides; stack autoencoders constructed ontological/semantic similarity of microbiota and diseases; and a stack autoencoder using Morgan molecular fingerprints.
Overall the article addresses a hot topic in biological data representation for machine learning applications. However, the language needs to be revised substantially to meet standards of scientific writing. I also highlight several technical issues that need to be addressed below.

Response: Thank to the reviewer for this positive comment.
Comment 1:
1. Strictly speaking not all the nodes used by the authors are biomarkers (such as drugs and diseases), a more general way to refer them would be to use the word "bioentities".
Response: Thanks for this useful comment.
According to the suggestions of the reviewer, we have corrected the relevant terms and highlight them in blue in the revised manuscript.

Comment 2:
2. Eqn 7: does the window definition require certain ordering of vertices (vi-w to vi+w)? If yes, how is it defined?
Response: This is a very important but overlooked issue. Thank the reviewers for reminding us to observe this problem.
The network is initially defined as an undirected graph. Meanwhile, we do not specify specific metapaths to control the order of vertices in random walk.

Comment 3:
3. Clarify the contents of Wvi (e.g., random walk score from vi to all other nodes?)
Response: Thanks for this useful comment.
Wvi is a random walk rooted from vertex vi. Wvi contains a series of vertexes including different types of bioentities. As we have said in comment 2. We do not require random walks to follow a specific order.

Comment 4:
4. What are the values of w and t used in the experiments and how are they optimized?
Response: Thank the reviewer for this comment.
We found that the effect of parameter w and t on the results is not obvious. At the same time, a smaller value of them can significantly reduce the running time of the experiment. We try to explain this phenomenon. The structure of the network we construct is totally different from previous benchmark datasets such as Facebook, twitter, etc. In traditional social networks, vertices of the same label are

closely related. In the network of this manuscript, there are generally no edges between vertices of the same label except for protein-protein interaction network. The representation of vertices is mainly through the description of relationship with other types of vertices. Larger w and t may introduce additional noise and increase calculation burden. At the same time, in order to ensure the experimental reproducibility as much as possible, we set the parameters w and t to the commonly used values 10 and 80. After generating the sequence of vertices, python package called gensim was applied to generate the word embedding representation.

Comment 5:
5. Algorithm 2, vi in line 1 should be vj. Also the denominator on line 4 is illegible.
Response: Thank you for your suggestion.
We are sorry this is caused by the Word format. Corrected parts are highlighted in blue in the revised manuscript.

Comment 6:
6. Clarify how the negative instances are selected for the classifier building and how the data is balanced (number of positive instances vs negative instances).
Response: Thank you for your helpful comments.
According to your comments, we add the generation process of positive and negative samples to the manuscript in flowchart in introduction, and highlight them in blue in revised manuscript. The positive samples are experimentally verified relationships and the negative samples are the same number of unlabeled relationships which are randomly selected in matrix A.
Comment 7:
7. It would be useful to have prediction metrics for models using different data types independently to see which attributes are easier / harder to predict.
Response: Thanks for this useful suggestion.
We are a little confused about your comments, but we do our best to explain the core of the manuscript. One goal of the proposed method is to predict multi-type relationships simultaneously. We don't focus on the prediction of single-type relationship prediction by different attributes. Experiments prove the effectiveness of this idea. Although imperfect, we hope this is the beginning of a global perspective to address such issues.
Comment 8:
8. To achieve a more practical representation of real world setting (i.e., drugs without previous information), drug-wise disjoint cross validation could be used (Guney 2017 https://doi.org/10.1142/9789813207813_0014).
Response: Thanks for this useful suggestion.
We read the literature you provided carefully. All experiments, including additional comparison experiments, were performed under real-world conditions. In each fold under cross-validation, we proceed strictly in the following order. Firstly, we divide the entire network into a training set and a test set. Secondly, we use the training set to construct the representation vector and train the classifier. Finally, we use the test set to evaluate the performance of the model. All operations try to ensure that the labels of the test set are not leaked into the representation vector and the construction of the classifier.
For example, the number of drug-disease associations is 17414. In each fold under 5 cross-validation, approximately 3483 association pairs will be removed firstly, and the remaining 13,931 association pairs will be used to construct the representation vectors and classifier. This operation was repeated five times.
Comment 9:
9. "Superior robustness and stability" compared to which other methods? A comparison of the results across different methods would be help positioning the proposed approach within the existing literature (some of which could also be referred in the introduction):
Ngo et al, 2016, http://dx.doi.org/10.4236/jbise.2016.91002
Su et al, 2018, https://doi.org/10.1093/bib/bby117
Shahreza et al, 2018 https://doi.org/10.1093/bib/bbx017
Martinez et al, 2015 https://doi.org/10.1016/j.artmed.2014.11.003
Zeng et al, 2019 https://doi.org/10.1093/bioinformatics/btz418
Wei et al, 2019 https://doi.org/10.1108/DTA-01-2019-0004 (also uses DeepWalk)
Response: Thanks for this useful suggestion.
We have read the literatures you provided carefully and benefited a lot. However, most of the methods proposed in these articles aim at drug relocation or drug-disease association prediction. The focus of our method is to predict multi-type relationships between bioentities. In addition, no specific data sets or pubmed abstract corpus were provided for use. Hence, it is difficult for us to compare the proposed

method with them.
However, inspired by these articles and we described them in the introduction as a separate paragraph to enrich and complement the research background. The added parts are highlighted in blue in the revised manuscript.
Comment 10:
10. Pg11 "have been collected from DrugBank" It was mentioned earlier that the drug-disease associations were from CTD, clarify & explain how the drug indications are identified.
Response: Thanks for this useful suggestion.
We are very sorry that the source of the relationship pair is not explained clearly in the original manuscript. Drug-disease associations were collected from CTD. Drug-target interactions were collected from DrugBank.
Comment 11:
12. Table 4, explain the columns of the table and what they refer to clearly (what do null & unconfirmed mean? Does the inference score come from CTD?
Response: Thanks for this useful suggestion.
The top-10 predicted drugs have been confirmed according to CTD database. All association pairs are verified by the CTD database. Inference Score and References are provided by CTD. The term ‘unconfirmed’ is an association pair that we cannot find in the CTD. We will add a detailed description of the form to the revised manuscript.

Comment 12:
12. What are the rankings of known Ataxia drugs (in Drugbank or CTD direct associations)?
Response: Thanks for this useful suggestion.
The rankings of known Ataxia drugs can be obtained in CTD at URL
http://ctdbase.org/detail.go?type=disease&acc=MESH%3aD001259&view=chem

Comment 13:
13. The following sentences lack references:
- Pg4: has been widely documented
- Pg5: NCBI
- Pg6: defined as 0.5 according (to) previous literature
Response: This is a very important but overlooked issue. Thank the reviewers for reminding us to observe this problem.
We supplement each of the required references and highlight them in blue in the revised manuscript.

Comment 14:
15. Language issues (requires revision / clarification)
Pg1
- re-recognize life activities within human cells
- biology (ical) system
- biomarker attribute and behaviors (unclear what behavior refers to)
- prediction task (prediction of what?)
Pg2
- microscopic research
- the trouble of information overload
- computational tool(s)
- Although many … lacks secondary clause
- FPR and FNR (abbr. used without definition)
- Biomolecules outside the research … (ambiguity over the whole sentence)
- deeply understand
- drawn support
- Tremendous advances … lacks main verb
- computational model(s)
Pg3
- landscape of biology system
- a complete network (formally a fully complete network is defined as a graph where all the nodes are connected to all others)
- online database(s)
- inseparable connections and messages flow?
- example like MAN
Pg4
- potential of them inferring potential relationships

- (MAN) x 2- abbr definition unnecessarily repeated
- Then we develop the lower triangular part
- the behavior feature - need to define what is behavior featutre
Pg5
- pretreatment (preprocessings)
- k nucleotide (or amino acid) combination
Pg6
- Inspired by D Wang
Pg8
- is not conducive to classifier training
- Keras lab (library / package?)
- hyperparameter(s) - also specify what are these parameters and their default values
Pg9
- MCC - define abbr
- in different situation(s)
Pg10
- the more distinguished … is the easier is .. and they achieve (also, this is a very long sentence, consider splitting)
- "new sample" problem (cold start?)
- testing and evaluation, (comma)
- experimental(ly) verified
Pg12
- chapter on feature (chapter?)
Pg13
- connected to Ataxia
- single logic
- isolated idea
- exquisite discovery
Response: Thanks for this useful suggestion.
According to the comments of the reviewer, we have checked the grammar, punctuation and spelling carefully. The details of the changes are highlighted in blue in the revised manuscript.

Again, we appreciate all of your insightful comments. We hope that we have addressed your concerns satisfactorily. Thank you for taking you time and energy to help us improve the paper.

Reviewer 2
Response: Thank you very much for helpful comments.

Comment 1:
Comment 1: Several references about link prediction on dynamic network can be added [1][2].
[1] Ozcan A, Oguducu SG. Link prediction in evolving heterogeneous networks using the NARX neural networks. Knowledge and Information Systems 2018;55(2):333-360, https://doi.org/10.1007/s10115-017-1073-x.
[2] Ozcan A, Oguducu SG. Multivariate Time Series Link Prediction for Evolving Heterogeneous Network. International Journal of Information Technology & Decision Making 2019;18(1):241-286, https://doi.org/10.1142/S0219622018500530.
Response: Thanks for this useful suggestion.
The use of time information is a hot topic in network research. According to the suggestions of the reviewer, more references about link prediction on dynamic network were added in the introduction to complement the background. The changed part is highlighted in blue in the revised manuscript.

Comment 2:
Comment 2: Authors should add more information and running examples about Fig. 2 (The flowchart of proposed method).
Response: Thank you for your precious comment.
We describe Fig. 2 in more detail and more changes can be seen in the manuscript. The title of Fig. 2 has been described as follows: Figure 2. The flowchart of the proposed method. Each node in the network can be described from 2 perspectives, one is the attribute feature such as sequence and chemical structure that can be learned as a 64-dimension vector by k-mer and etc. methods, the other is the behavior feature that is the relationships which can be represented as a 64-dimension vector through DeepWalk. After combining above 2 information, each node can be represented as a 128-dimension vector. The positive samples are experimentally verified relationships and the negative

samples are the same number of unlabeled relationships which are randomly selected in A. Taking the low-dimensional dense vectors as input, random forest is used to carry out the prediction task.

Comment 3:
Comment 3: Can you visualize the DeepWalk and SkipGram algorithms? Adding detailed drawing and running examples regarding the DeepWalk and SkipGram algorithms will be better.
Response: Thanks for this useful suggestion.
According to the suggestions of the reviewer, we add a visualization of DeepWalk. The changed part is highlighted in blue in the revised manuscript.

Comment 4:
Comment 4: Authors should send and share source code of the proposed model like python etc.
that contains README file and example dataset used in manuscript? And also can you share the running examples of the proposed model outputs.
Response: Thank you for your helpful comments.
According to the suggestions of the reviewer, we have uploaded the source code and data to github. The URL is https://github.com/CocoGzh/Biomarker2vec.

Comment 5:
Comment 5: Authors should extend the model by using the time information. Also they extend the proposed model for the evolving heterogeneous network and dynamic link prediction.
Response: Thanks for this useful suggestion.
We agree with and appreciate the comments. However, considering that we collect data from static databases, it is very difficult for us to add time information. We will refer to the comments to improve and construct the new model in the future.

Again, we appreciate all of your insightful comments. We hope that we have addressed your concerns satisfactorily. Thank you for taking you time and energy to help us improve the paper.

Close
