# Supplementary material for: Bioentity2vec: Attribute- and behavior-driven representation for predicting multi-type relationships between bioentities
Source: Gigascience. 2020 Jun 13;9(6):giaa032. doi: 10.1093/gigascience/giaa032 (PMC7293023; doi:10.1093/gigascience/giaa032)
Supplement: giaa032_Response_to_Reviewer_Comments_Revision_1 [file giaa032_response_to_reviewer_comments_revision_1.pdf]

Author's Response To Reviewer Comments

Close

Authors' Response to Reviewers' Comments
Paper title: Biomarker2vec: Attribute- and Behavior-driven Representation for Multi-type Relationship Prediction between Various Biomarkers
Manuscript ID: GIGA-D-19-00385R1
Authors: Zhen-Hao Guo; Zhu-Hong You; De-Shuang Huang; Hai-Cheng Yi; Yan-Bin Wang; Zhan-Heng Chen

We are grateful to the editor and reviewers for putting in efforts to review the paper with the aim of improving the quality of our paper. We have addressed the concerns of the editor and reviewers in the revised manuscript. In particular, the following revisions have been made.

Reviewer 1
I thank authors for addressing my comments. Few additional points:
Response: Thank to the reviewer for this positive comment.
Comment 1:
1. Fig 2, entities are still referred as biomarker. Additionally, what the dashed borders / lines mean should be specified.
Response: Thanks for this useful comment.
According to the suggestions of the reviewer, we corrected the picture and added a description of the dashed borders / lines.

Comment 2:
2. Answer to my previous question #4 (optimization of parameters, usage of gensim) should be included/discussed in the text.
Response: Thanks for this useful comment.
The response has been discussed and highlighted in blue in the revised manuscript.

Comment 3:
3. To demonstrate the practical utility of the predictions, Table 4 should include the name and ranking of the drug as per the known drugs in CTD in addition to the inference score and number of references (as commented in #12, the http://ctdbase.org/detail.go?type=disease&acc=MESH%3aD001259&view=chem). It would also be beneficial, what is the accuracy of their method on predicting the top 10,20,50,100 drugs in CTD (as well as a relevant discussion on these results).
Response: Thanks for this useful comment.
We have converted the IDs of the drugs in DrugBank to the CTD chemical names and added them to Table 4. We sorted all drugs by Direct Evidence, and the results of top-10 were added to Table 4. Considering that many top drugs in CTD do not appear in the network we construct, the prediction of these drugs actually uses only the attributes of the drug, that is, the molecular structure of the drug. Therefore, it is difficult to reflect the advantages of our proposed method. We add these discussions in the reviewed manuscript and highlight them in blue.

Again, we appreciate all of your insightful comments. We hope that we have addressed your concerns satisfactorily. Thank you for taking you time and energy to help us improve the paper.

Reviewer 2
The methods are appropriate to the aims of the study, they are well described, and necessary controls are included. The conclusions are adequately supported by the data shown. The quality of language in the manuscript is clear.
Response: Thank you very much for helpful comments.

Comment 1:
The statistical tests used in manuscript is not appropriate. t-test or any other related statistical test is

used in order to compare the proposed model with baseline.
Response: Thanks for this useful suggestion.
As the reviewer said, t-test is an evaluation method widely used in bioinformatics. T-test or any other related statistical test is the more acceptable result. However, the proposed method is a deep learning-based representation algorithm and machine learning-based prediction model. Therefore, we prefer to use computer science evaluation methods. Our follow-up work will complement and improve it.

Again, we appreciate all of your insightful comments. We hope that we have addressed your concerns satisfactorily. Thank you for taking you time and energy to help us improve the paper.

Close
