# Supplementary material for: Bioentity2vec: Attribute- and behavior-driven representation for predicting multi-type relationships between bioentities
Source: Gigascience. 2020 Jun 13;9(6):giaa032. doi: 10.1093/gigascience/giaa032 (PMC7293023; doi:10.1093/gigascience/giaa032)
Supplement: giaa032_Reviewer_1_Report_Original_Submission — Emre Guney -- 12/15/2019 Reviewed [file giaa032_reviewer_1_report_original_submission.pdf]

Reviewer Report

Title: Bioentity2vec: Attribute- and Behavior-driven Representation for Multi-type Relationship Prediction between Various Bioentities

Version: Original Submission **Date:** 12/15/2019

Reviewer name: Emre Guney

Reviewer Comments to Author:

Guo an colleagues present biomarker2vec, an approach to represent biomolecule as features in low dimensional representation space and provide an application of these features in the prediction of drug-disease relationships using a random forest based classifier. A molecular association network (MAN) is generated using data from various biological databases in which nodes and edges are represented as 64 dimensional vectors. Nodes are described based on the occurrences of 3-mers using a 4-letter alphabet for proteins and nucleotides; stack autoencoders constructed ontological/semantic similarity of microbiota and diseases; and a stack autoencoder using Morgan molecular fingerprints.
Overall the article addresses a hot topic in biological data representation for machine learning applications. However, the language needs to be revised substantially to meet standards of scientific writing. I also highlight several technical issues that need to be addressed below.
1. Strictly speaking not all the nodes used by the authors are biomarkers (such as drugs and diseases), a more general way to refer them would be to use the word "bioentities".
2. Eqn 7: does the window definition require certain ordering of vertices (vi-w to vi+w)? If yes, how is it defined?
3. Clarify the contents of Wvi (e.g., random walk score from vi to all other nodes?)
4. What are the values of w and t used in the experiments and how are they optimized?
5. Algorithm 2, vi in line 1 should be vj. Also the denominator on line 4 is illegible.
6. Clarify how the negative instances are selected for the classifier building and how the data is balanced (number of positive instances vs negative instances).
7. It would be useful to have prediction metrics for models using different data types independently to see which attributes are easier / harder to predict.
8. To achieve a more practical representation of real world setting (i.e., drugs without previous information), drug-wise disjoint cross validation could be used (Guney 2017 https://doi.org/10.1142/9789813207813_0014).
9. "Superior robustness and stability" compared to which other methods? A comparison of the results across different methods would be help positioning the proposed approach within the existing literature (some of which could also be referred in the introduction):
Ngo et al, 2016, http://dx.doi.org/10.4236/jbise.2016.91002
Su et al, 2018, https://doi.org/10.1093/bib/bby117
Shahreza et al, 2018 https://doi.org/10.1093/bib/bbx017
Martinez et al, 2015 https://doi.org/10.1016/j.artmed.2014.11.003
Zeng et al, 2019 https://doi.org/10.1093/bioinformatics/btz418

Wei et al, 2019 https://doi.org/10.1108/DTA-01-2019-0004 (also uses DeepWalk)
10. Pg11 "have been collected from DrugBank" It was mentioned earlier that the drug-disease associations were from CTD, clarify & explain how the drug indications are identified.
12. Table 4, explain the columns of the table and what they refer to clearly (what do null & unconfirmed mean? Does the inference score come from CTD?
13. What are the rankings of known Ataxia drugs (in Drugbank or CTD direct associations)?
14. The following sentences lack references:
- Pg4: has been widely documented
- Pg5: NCBI
- Pg6: defined as 0.5 according (to) previous literature
15. Language issues (requires revision / clarification)
Pg1
- re-recognize life activities within human cells
- biology (ical) system
- biomarker attribute and behaviors (unclear what behavior refers to)
- prediction task (prediction of what?)
Pg2
- microscopic research
- the trouble of information overload
- computational tool(s)
- Although many … lacks secondary clause
- FPR and FNR (abbr. used without definition)
- Biomolecules outside the research … (ambiguity over the whole sentence)
- deeply understand
- drawn support
- Tremendous advances … lacks main verb
- computational model(s)
Pg3
- landscape of biology system
- a complete network (formally a fully complete network is defined as a graph where all the nodes are connected to all others)
- online database(s)
- inseparable connections and messages flow?
- example like MAN
Pg4
- potential of them inferring potential relationships
- (MAN) x 2- abbr definition unnecessarily repeated
- Then we develop the lower triangular part
- the behavior feature - need to define what is behavior featutre
Pg5
- pretreatment (preprocessings)
- k nucleotide (or amino acid) combination

Pg6
- Inspired by D Wang
Pg8
- is not conducive to classifier training
- Keras lab (library / package?)
- hyperparameter(s) - also specify what are these parameters and their default values
Pg9
- MCC - define abbr
- in different situation(s)
Pg10
- the more distinguished … is the easier is .. and they achieve (also, this is a very long sentence, consider splitting)
- "new sample" problem (cold start?)
- testing and evaluation, (comma)
- experimental(ly) verified
Pg12
- chapter on feature (chapter?)
Pg13
- connected to Ataxia
- single logic
- isolated idea
- exquisite discovery

Methods

Are the methods appropriate to the aims of the study, are they well described, and are necessary controls included? Choose an item.

Conclusions

Are the conclusions adequately supported by the data shown? Choose an item.

Reporting Standards

Does the manuscript adhere to the journal's guidelines on minimum standards of reporting? Choose an item.

Choose an item.

Statistics

Are you able to assess all statistics in the manuscript, including the appropriateness of statistical tests used? Choose an item.

Quality of Written English

Please indicate the quality of language in the manuscript: Choose an item.

Declaration of Competing Interests

Please complete a declaration of competing interests, considering the following questions:

- Have you in the past five years received reimbursements, fees, funding, or salary from an organisation that may in any way gain or lose financially from the publication of this manuscript, either now or in the future?
- Do you hold any stocks or shares in an organisation that may in any way gain or lose financially from the publication of this manuscript, either now or in the future?
- Do you hold or are you currently applying for any patents relating to the content of the manuscript?
- Have you received reimbursements, fees, funding, or salary from an organization that holds or has applied for patents relating to the content of the manuscript?
- Do you have any other financial competing interests?
- Do you have any non-financial competing interests in relation to this paper?

If you can answer no to all of the above, write 'I declare that I have no competing interests' below. If your reply is yes to any, please give details below.

I declare that I have no competing interests

I agree to the open peer review policy of the journal. I understand that my name will be included on my report to the authors and, if the manuscript is accepted for publication, my named report including any attachments I upload will be posted on the website along with the authors' responses. I agree for my report to be made available under an Open Access Creative Commons CC-BY license (http://creativecommons.org/licenses/by/4.0/). I understand that any comments which I do not wish to be included in my named report can be included as confidential comments to the editors, which will not be published.

Choose an item.

To further support our reviewers, we have joined with Publons, where you can gain additional credit to further highlight your hard work (see: https://publons.com/journal/530/gigascience). On publication of this paper, your review will be automatically added to Publons, you can then choose whether or not to claim your Publons credit. I understand this statement.

Yes Choose an item.
